# Mammalian Small Nucleolar RNAs Are Mobile Genetic Elements

**DOI:** 10.1371/journal.pgen.0020205

**Published:** 2006-12-08

**Authors:** Michel J Weber

**Affiliations:** 1 Laboratoire de Biologie Moléculaire Eucaryote, CNRS/Université de Toulouse-Paul Sabatier, Toulouse, France; 2 Institut d'Exploration Fonctionnelle des Génomes, CNRS/Université de Toulouse-Paul Sabatier, Toulouse, France; Fred Hutchinson Cancer Research Center, United States of America

## Abstract

Small nucleolar RNAs (snoRNAs) of the H/ACA box and C/D box categories guide the pseudouridylation and the 2′-O-ribose methylation of ribosomal RNAs by forming short duplexes with their target. Similarly, small Cajal body–specific RNAs (scaRNAs) guide modifications of spliceosomal RNAs. The vast majority of vertebrate sno/scaRNAs are located in introns of genes transcribed by RNA polymerase II and processed by exonucleolytic trimming after splicing. A bioinformatic search for orthologues of human sno/scaRNAs in sequenced mammalian genomes reveals the presence of species- or lineage-specific sno/scaRNA retroposons (sno/scaRTs) characterized by an A-rich tail and an ∼14-bp target site duplication that corresponds to their insertion site, as determined by interspecific genomic alignments. Three classes of snoRTs are defined based on the extent of intron and exon sequences from the snoRNA parental host gene they contain. SnoRTs frequently insert in gene introns in the sense orientation at genomic hot spots shared with other genetic mobile elements. Previously characterized human snoRNAs are encoded in retroposons whose parental copies can be identified by phylogenic analysis, showing that snoRTs can be faithfully processed. These results identify snoRNAs as a new family of mobile genetic elements. The insertion of new snoRNA copies might constitute a safeguard mechanism by which the biological activity of snoRNAs is maintained in spite of the risk of mutations in the parental copy. I furthermore propose that retroposition followed by genetic drift is a mechanism that increased snoRNA diversity during vertebrate evolution to eventually acquire new RNA-modification functions.

## Synopsis

Large parts of vertebrate genomes are made of repeated sequences that were first considered to be junk DNA, but are now recognized as important actors in genome evolution. Most are genetic mobile elements that can gain additional genomic copies by a copy-and-paste mechanism involving an RNA intermediate. One class, the L1 elements, encodes two proteins required for its integration at new sites. Others, like primate *Alu* elements, hijack the L1 machinery for their mobilization, and are thus referred to as nonautonomous. In this article, Weber describes a new class of vertebrate nonautonomous mobile elements derived from small nucleolar RNAs (snoRNAs). These nonprotein-coding RNAs are encoded in gene introns and are involved in chemical modifications of selected bases of ribosomal RNAs. The article shows that new snoRNA copies were generated in vertebrate genomes via the copy-and-paste mechanism. Many of them are species-specific, and their insertion point was precisely determined by alignment with the corresponding genomic portion from a neighbour species. The mobilization of snoRNA gene sequences might ensure the presence of a functional copy when the parental one becomes invalidated by mutations. Moreover, such copies could evolve on their own to acquire the capacity of guiding new modifications of ribosomal RNAs.

## Introduction

About 45% of the human genome is composed of transposable elements that are classified as long interspersed elements (LINEs, 21%), short interspersed elements (SINEs, 13%), retrovirus-like elements (8%), and DNA transposon fossils (3%) [1]. Transposition-competent LINEs encode ORF1, an RNA-binding protein that coats the L1 RNA [2], and ORF2, which is endowed with both endonuclease (EN) and reverse-transcriptase (RT) activities [3,4]. Both L1-encoded proteins predominantly mobilize the RNA that encodes them, a phenomenon referred to as *cis* preference [5–7]. The transposition of such repeat elements to new genomic sites occurs by a mechanism called target-primed reverse transcription, where the ORF2 EN activity creates a 3′ hydroxyl that is used as primer for the reverse transcription of L1 RNA [3,8,9]. The second strand of the L1 cDNA is then synthesized via a still unclear mechanism, following a staggered nick on the other strand of the host DNA, so that the newly inserted element is bracketed by an ∼14–base pair (bp) direct repeat called the target site duplication (TSD). In addition, L1 elements can insert at double-stranded breaks caused by DNA damage independently of ORF2 EN activity, a phenomenon exacerbated in cells deficient for the nonhomologous end-joining DNA-repair pathway [10]. Indeed, integration of L1 elements in tissue-culture cells requires components of the double-strand break–repair machinery [11]. About half of the most recently inserted L1 elements are polymorphic among human populations [12].

*Alu* elements are derived from an ancient dimerization of the 7SL component of the signal recognition particle, of which the *AluY* subclass is still active in the human genome [13,14] and can be polymorphic ([15] and references therein). A second class of nonautonomous hominoid-specific retroposons, called SVA, contains SINE-R and *Alu* elements separated by a variable nucleotide tandem repeat. Of the ∼5,000 SVA copies present in the human genome, ∼80% are absent in chimpanzees, demonstrating a recent mobilization [16–19]. Finally, a rodent-specific SINE element, ID, originating from the BC1 noncoding RNA, has recently amplified in the rat genome. One such element is polymorphic among rat laboratory strains [20]. These three classes of SINE elements are devoid of protein-coding capacity and most probably use the L1 ORF2 protein for their retroposition as *Alu* elements [14]. A similar mechanism has been proposed for the insertion of processed pseudogenes [5]. Mobile elements of the L1 and *Alu* families are the root cause of several human and mouse diseases as a result of nonhomologous recombination, gene conversion, and insertional mutation events [21].

In this article, I describe a new family of nonautonomous transposable elements derived from the small nucleolar RNAs (snoRNAs) of mammalian genomes. These short, noncoding RNAs belong to two classes, the C/D box and the H/ACA box snoRNAs, which serve as guides for the 2′-O-ribose methylation and pseudouridylation (PU), respectively, of selected bases of ribosomal RNAs by base-pairing mechanisms [22–26]. C/D box snoRNAs are characterized by the presence of consensus C (RUGAUGA) and D (CUGA) motifs juxtaposed to a short terminal stem and are associated in C/D small nucleolar ribonucleoparticles (snoRNPs) with four core proteins: fibrillarin (the methyltransferase enzyme), NOP56 (NOL5A), NOP5/NOP58, and NHP2L1. The H/ACA snoRNAs are composed of two imperfect stem loops separated by a single-stranded hinge that contains the H box (ANANNA) and a short tail containing the ACA motif. The core H/ACA snoRNP contains four proteins: DKC1 (dyskerin, the pseudouridine synthase), GAR1 (NOLA1), NHP2 (NOLA2), and NOP10 (NOLA3). In addition, a third class of guide RNAs, the Cajal body–specific RNAs (scaRNAs), is involved in the 2′-O-ribose methylation and PU of small nuclear RNAs of the spliceosome [27]. scaRNAs can be of the C/D or H/ACA type, or can comprise a H/ACA domain embedded in a C/D box structure [27–29]. The Cajal body–localization signal, called the CAB box (consensus: UGAG), is found in the loops of H/ACA scaRNAs [30] and serves to recruit specific Sm proteins [31]. Most vertebrate snoRNAs reside in introns of genes, although a small number are generated from independent polymerase II transcription units [32].

In the course of a systematic search for the orthologues of experimentally evidenced human and murine snoRNAs in 17 vertebrate genomes, it appears that most of them have several paralogues that frequently reside in gene introns in the sense orientation. Examination of the sequences surrounding these new snoRNA-gene copies established that most of them are part of retroposons, which I have called snoRNA retroposons (snoRTs) and scaRNA retroposons (scaRTs). In many cases, genomic sequence alignments showed that the associated retroposition events are species- or lineage-specific among the sequenced vertebrate genomes, permitting a precise delineation of the insertion point. As 375 human sno/scaRNAs are presently known [33], their number is comparable to that of transposition-active L1 and *Alu* elements. By analogy, retroposition of snoRNA genes might have played an important role in the modern evolution of mammalian genomes.

## Results

A systematic search for orthologues of human and murine H/ACA snoRNAs and scaRNAs in sequenced vertebrate genomes was performed using BLAT [34]. Among significant hits, a snoRNA orthologue was further defined by a BLAT search of the human host gene (HG) mRNA or protein sequence. Many orthologous snoRNA genes could thus be identified from human to fish genomes. The corresponding alignments are presented on the snoRNABase at http://www-snorna.biotoul.fr/ [33]. A case-by-case examination revealed that snoRNA paralogues can originate from duplications of the HG, particularly in pericentromeric regions, or by intragenic duplication in different introns of the same HG (unpublished data).

**Figure 1:**
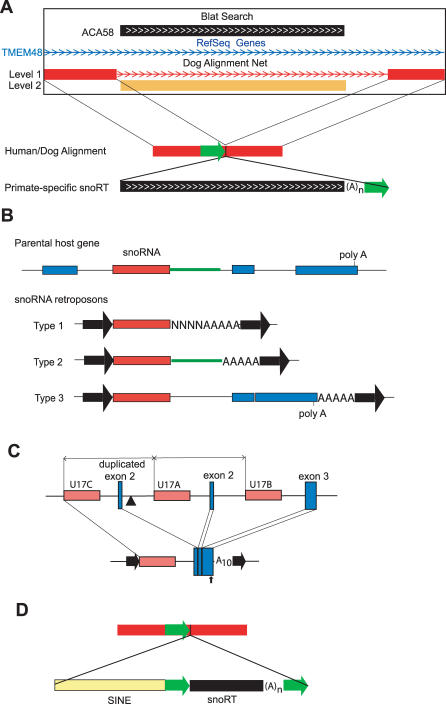
Schematic Representation of snoRTs (A) Analysis of a primate-specific snoRT. Upper panel: localization of the snoRT. A Blat search with the human ACA58 snoRNA sequence (hosted in the gene UBAPL2) localizes a close copy in the human TMEM48 gene. The Dog Alignment Net track of the UCSC Genome Browser shows two syntheny levels. Level 1 corresponds to the dog TMEM48 gene, but Level 2 corresponds to the dog ACA48 orthologue in the UBAPL2 gene. The Chimpanzee Alignment Net track shows complete syntheny with the human TMEM48 gene (unpublished data). Lower panel: characterization of the primate-specific ACA58 snoRT and determination of the insertion point. The “view alignment details of parts of net within browser window” tool of Dog Net track (Level 1) gives the alignment of the synthenic portions of the human and dog genomes (schematized on upper panel). Examination of the human sequence allows recognition of the polyA tail [(A)n] and the TSD (green arrows) and precise localization of the snoRT insertion point at one extremity of the TSD (lower panel). The detailed alignment is given in the Figure S1, section 13. (B) Description of the three types of snoRTs. The upper drawing shows the structure of the parental snoRNA HG. Blue and red boxes represent exons and the snoRNA sequence, respectively. The drawings below show the structures of Type 1–3 snoRTs. The partial snoRNA downstream intronic segment included in Type-2 retroposons is indicated by a green line. (C) Duplication of U17A in the cow U17 HG and structure of a cow snoRT. U17 sequences and exons are represented by red and blue rectangles, respectively (not to scale). The duplicated segment is indicated by horizontal arrows. A similar duplication is found in the armadillo U17 HG gene, with an additional 430-bp insertion indicated by the black arrowhead. The structure of a cow snoRT containing U17C and U17 HG exons is shown below. This retroposon (chr19:44122615–44123602) is located in an intron of the TBCD gene in the sense orientation and is composed of the U17C sequence and downstream intron in addition to the duplicated copies of exon 2 and exon 3. Black horizontal arrows indicate the TSD. The vertical arrow indicates the position of a consensus polyadenylation signal located 16 bp upstream of the genomic polyA tail. See Figure S3, section 9 for sequence. (D) Insertion of a SINE element and a snoRT at a common site. In certain cases, genomic alignments with an outlier species with no snoRT show that the snoRT (black box) and another nonautonomous mobile element such as a SINE (yellow box) are inserted at a common site, creating a triplication of the insertion site (green arrows).

Interestingly, the vast majority of the snoRNA gene paralogues displayed characteristics of retroposons, including a short A-rich tail and a 7–19-bp TSD (Figure S4). In most cases, such retroposons are species- or lineage-specific, and the alignment of genomic sequences from closely related species resulted in the determination of the precise insertion point at one extremity of the TSD (Figure 1A). Three types of snoRTs were distinguished depending on the amount of the genomic sequences retroposed with the snoRNA gene (Figure 1B). Type-1 snoRTs are composed of the snoRNA sequence, fully matured at its 5′ end, followed by 0–9 additional bases and a short A-rich tail, bracketed by a TSD. In Type-2 snoRTs, the snoRNA sequence is followed by a large part of the downstream intronic sequence from the parental HG. Type-3 snoRTs contain the snoRNA sequence, again fully matured at its 5′ end, the entire downstream intronic sequences, and the fully processed downstream exons of the parental gene (Figure 1B). These retroposons are described in detail below, including more complex snoRTs containing a snoRNA retrocopy and additional repeat elements.

### Type-1 snoRNA Retroposons

Examples of Type-1 snoRTs of various H/ACA-box snoRNAs in mammalian genomes are presented in Table 1 and Figure S1 and share the following characteristics. Their 5′ end coincides with that of the fully matured parental snoRNA, with the occasional addition of 1–10 upstream untemplated nucleotides (nts). The position corresponding to the 3′ end of the mature snoRNA is followed by 0–9 bases that, in most cases, originate from the intronic sequence located immediately downstream of the snoRNA sequence in the parental gene. They also contain a 5–30 bp-long polyA tail, often interrupted by G's and, more rarely, by T's and C's. In some cases, this tail is composed of the repetition of a motif such as (A)_3–7_G or AC (Figure S1, sections 5, 7, 11, and 20) or, in one case, of a 270-bp tail mostly composed of AAAG repeats (Figure S1, section 24). Similar compositions were reported for human L1 polyA tails [35]. Finally, most Type-1 snoRTs are bracketed by a 8–19 bp TSD (Figure S4). However, some are devoid of TSD (Figure S1, sections 16, 24, 26, and 31), and their insertion appears to have been accompanied by a small deletion (Figure S1, section 24) or insertion of short sequences of unknown origin (Figure S1, section 16). Most probably, these were inserted at DNA breaks by a DNA-repair machinery independently of the L1 EN activity [10].

**Table 1:**
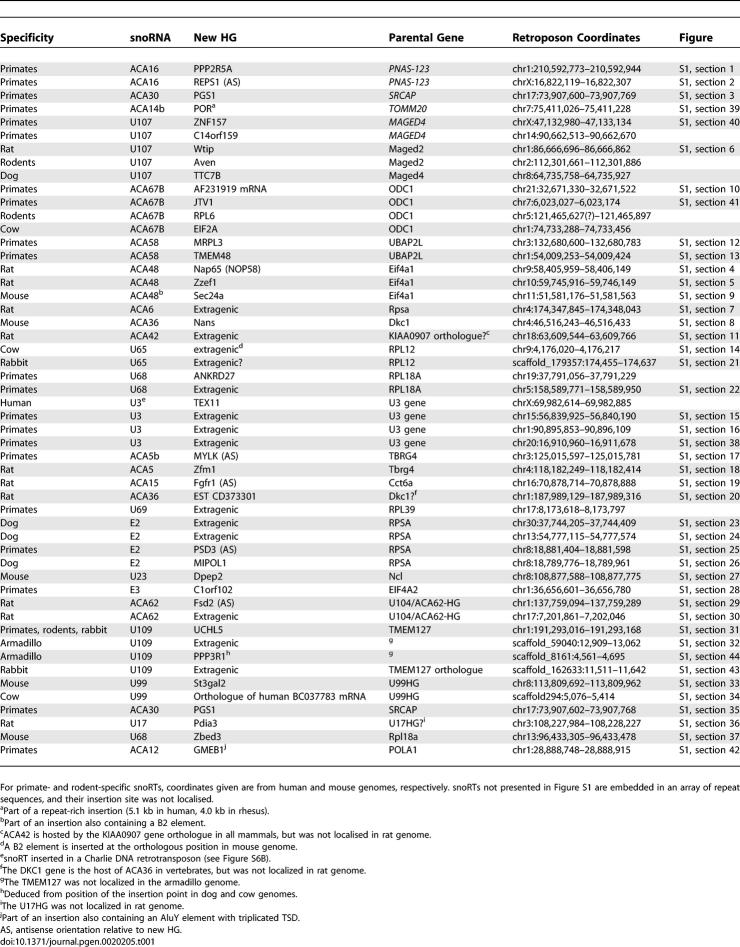
Examples of Mammalian Type 1 snoRTs

Many of these snoRTs are species- or lineage-specific and their insertion point could be precisely mapped at one extremity of the TSD by alignment of genomic sequences from a related species (Figures 1A and S1). snoRTs are frequently located in the sense orientation in introns of known genes, so that the retrocopy can be correctly processed as a snoRNA. Strikingly, a rat-specific ACA48 snoRT resides in intron 1 of the *Nap65* (human *NOP58*) gene that encodes one of the core proteins of the C/D box snoRNPs (Figure S1, section 4). In higher vertebrates, including rat, this gene is also the host of the orthologues of the two snoRNAs HBII-234 and HBII-95 in introns 3 and 9, respectively. Although the insertion of a new snoRNA gene in the rat *Nap65* gene occurred by chance, genetically linking different nonessential snoRNAs might increase the selective pressure to retain them all, thus protecting the organism against gradual loss of the individual snoRNAs.

A Type-1 retroposon of the U109 scaRNA, U109B, located in the *UCHL5* gene, is present in the primate, rodent, and rabbit, but not cow and dog, genomes (Figure S1, section 31). The hypothesis of insertions of two lineage-specific U109 scaRTs at the same genomic site cannot be excluded, but is disfavored by the examination of phylogenetic trees (unpublished data). The most parsimonious hypothesis is that a single retroposition event occurred early in mammalian evolution, after the Laurasiatheria–Euarchontoglires split (94 million years ago [mya]), but before the separation of primates, rodents, and lagomorphs (77–85 mya), and, thus, before the extinction of dinosaurs (65 mya) at the Cretaceous–Tertiary boundary [36]. Despite this extraordinarily long evolutionary time, all functional elements of U109 are conserved in U109B, including the H, ACA, and CAB boxes and the PU guiding sequences (see alignment on snoRNABase [http://www-snorna.biotoul.fr/snosync/phyl_img/U109.gif]), suggesting a strong selective pressure to maintain two functional copies of the U109 scaRNA. In addition, U109 scaRNA might still be actively mobilized at present, as suggested by several species-specific retroposons (Figure S1, sections 32, 43, and 44). This establishes that sno/scaRNAs, together with CORE-SINE and AmnSINE1 elements [37,38], are among the oldest and longest-lived nonautonomous genetic mobile elements in mammalian genomes.

### Type-2 snoRNA Retroposons

Type-2 snoRTs defined here differ from Type-1 sequences as they contain a substantial part of the downstream intronic sequence from the parental snoRNA HG. Examples are presented in Table 2 and Figure S2. In rare cases (Figure S2, section 3 and unpublished data) the entire downstream intron is included up to its 3′ splice site. In four other cases, the 3′-most 3–17 bp of the intron are not included, suggesting that the polyadenylation of the retroposed RNA species occurred at or near the intron branch point (Table 2). In two cases where only the 5′-most part of the downstream intron is included, polyadenylation most probably occurred at the level of a cryptic polyA signal (Figure S2, sections 1 and 6), as if the retroposed RNA species had been matured as a snoRNA at its 5′ end and as an mRNA at its 3′ end. Complex events can thus accompany snoRNA retroposition.

**Table 2:**
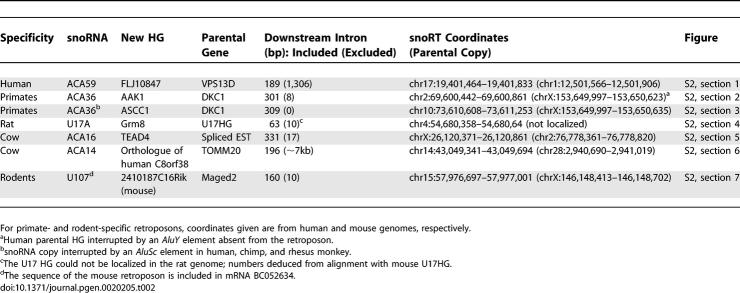
Examples of Mammalian Type 2 snoRTs

The insertion point of Type-2 snoRTs could be defined in most cases by interspecific genomic alignments and, as for Type-1 snoRTs, coincides with an extremity of the TSD. Most Type-2 snoRTs listed in Table 2 are located in an intron of their new HG in the sense orientation and can thus be processed as functional snoRNA copies. However, an *AluSc* element is inserted in an ACA36 snoRT in the three primate genomes, but is absent in the parental *DKC1* gene (Figure S2, section 3). This, and other examples discussed below, suggests that snoRTs constitute a favorable environment for the insertion of other mobile elements. Reciprocally, the human, but not chimpanzee and rhesus, *DKC1* gene contains an *AluYb8* element located 49–360 bp downstream of theACA36 gene that is absent from ACA36 Type 2 snoRTs (Figure S2, sections 2 and 3). Therefore, this *Alu* element was inserted in the human *DKC1* gene after the retroposition event.

### Type-3 snoRNA Retroposons

Type-3 snoRTs constitute an extreme example of hijacking in the process of retroposition. As in the case of Types 1 and 2 species described above, they start at the 5′ end the mature snoRNA (although truncated versions, presumably resulting from arrest of the RT, were also found), but contain in addition the entire downstream intronic sequence from the parental gene, followed by the processed 3′ exons and a polyA tail (Table 3 and Figure S3). Again, the insertion point coincides in most cases with one extremity of the TSD.

**Table 3:**
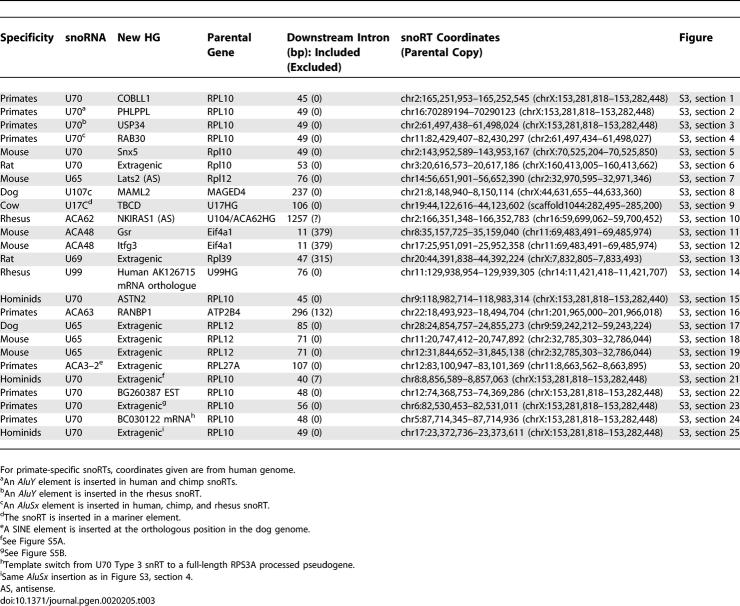
Examples of Mammalian Type 3 snoRTs

Species- or lineage-specific Type-3 snoRTs of the H/ACA box U70 snoRNA are particularly frequent. From fishes to human, the U70 gene resides in the penultimate intron of the *RPL10* gene (see alignment on the snoRNABase [http://www-snorna.biotoul.fr/snosync/phyl_img/U70.gif]). Among 42 human *RPL10* processed pseudogenes, 12 contain only *RPL10* exonic sequences, while 30 are Type-3 U70 snoRTs. The latter contain either the entire (18) or a partial (12) snoRNA sequence and the entire downstream part of the *RPL10* intron, in addition to the processed last two exons. Therefore, the mobilization of *RPL10*, together with the U70 snoRNA sequence, constitutes the major mode of retroposition for the RPL10 gene in the human genome. Moreover, twelve Type-3 U70 snoRTs reside in the sense orientation in an intron of a gene, and several of them are primate-specific (Figure S3, sections 1–4). A Type-3 U70 snoRT in the *ASTN2* gene is present in humans and chimpanzees, but not in rhesus monkeys (Figure S3, section 15). The complex structure of this insertion is discussed below.

Similar Type-3 U70 retroposons were found in the rat (two), mouse (three), elephant (three), and rabbit (eight) genomes; some of them are rat- or mouse-specific (Figure S3, sections 6 and 7). However, no U70 snoRTs were identified in the opossum and *Xenopus* genomes. One conclusion from this analysis is that U70 Type-3 retroposons have expanded during the evolution of higher vertebrates, a process that is still ongoing as demonstrated by the characterization of species-specific retroposons.

Surprisingly, a cow-specific U17 Type 3 snoRT in the *TBCD* gene contains a duplication of the second exon of its HG (Figure S3, section 9; and Figure S6A). In the human and mouse genomes, two copies of this snoRNA, U17A and U17B, are hosted in introns 1 and 2 of the U17HG noncoding gene [39]. In the cow genome, a 930-bp duplication in the *U17HG* gene extends from the 5′ end of the U17A snoRNA sequence to the 5′ end of U17B, thus creating a third U17C snoRNA copy and a duplication of exon 2 (Figure 1C). Therefore, the retroposon identified in the cow *TBCD* gene is in fact a Type-3 snoRT of the cow-specific U17C snoRNA. This example illustrates how intragenic duplication and retroposition can both create new snoRNA copies. This is further shown by the presence in the human genome of five highly similar copies of a distant U109 Type snoRT on Chr9 (>98% identity over 962–942 bp) and one on Chr4 (95.5% identity over 909 bp), as parts of larger segmental duplications. Therefore, the snoRNA family can expand by repeated birth-and-death mechanisms [40] in addition to retroposition.

Type-3 snoRTs analyzed so far contain the entire intronic sequence located downstream of the snoRNA sequence in the parental gene. However, in the case of a rat-specific U69 snoRT, only the 5′-most 41 bp of the downstream intron are included in the retroposon and ligated to the last exon of the parental *Rpl39* gene; a deletion of the 3′-most 315 bp of the intron thus accompanied retroposition (Figure S3, section 13). This truncation corresponds to a splicing event using a cryptic donor site in the intron of the parental gene. In contrast, other similar cases could not be accounted for by the use of a cryptic splicing site, underscoring the existence of complex mechanisms for the generation of Type-3 snoRTs (Figure S3, sections 11, 12, and 16).

Type 3 snoRTs bear similarities with the transduction of 3′ sequences by L1 elements, where transcription ignores the polyadenylation signal of the L1 element, but rather uses that of the next downstream-located gene. Therefore, exonic sequences mobilized by L1 elements could be integrated in a different gene, a process referred to as exon shuffling [41,42]. Processed exons from Type 3 snoRTs are delimited by a splicing acceptor site and a consensus polyadenylation site, and could thus be integrated in their HG by an alternative splicing mechanism, thus providing the corresponding protein with a different C-terminal domain. No such case was encountered so far. However, snoRTs were found to be partially included in exons from expressed sequence tags (ESTs) (Figure S3, sections 21–23; and Figure S5). In particular, a U70 snoRT on Chr6 brings the second exon of an EST, although in the opposite orientation (Figure S3, section 23, and Figure S5B). Therefore, species- or lineage-specific snoRTs can participate in building new transcription units and/or alternative exons of preexisting genes.

### Retroposons from Uncharacterized snoRNA Host Genes

**Figure 2:**
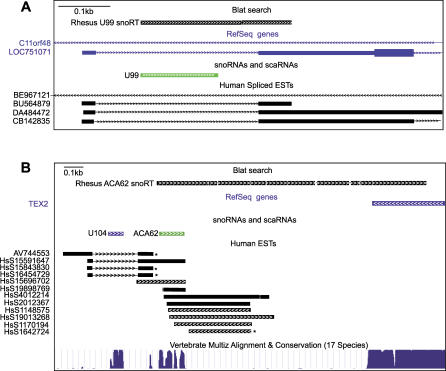
Characterization of snoRNA Host Genes by Analysis of snoRNA Retroposons (A) The human U99 HG. This figure, redrawn from a UCSC Genome Browser screen, shows results of the alignment of a rhesus-specific U99 snoRT with the human genome (Blat Search track), and the position of the human U99 snoRNA gene and of human spliced ESTs. For clarity, only 4/45 spliced ESTs in this window are shown. The human sequence orthologous to the retroposon starts at the 5′ end of U99 and ends at the 3′ end of six ESTs, like BU564879. The corresponding transcription unit, the U99HG (RefSeq gene LOC751071), resides in an intron of the C11orf44 gene in the antisense orientation. The BE967121 EST corresponds to the C11orf48 gene, but the others were erroneously included in the Hs.9061 (C11orf48) UniGene cluster. Window shown: chr11:62,189,300–62,190,050 (hg18). (B) The human U104/ACA62 HG. This UCSC screen copy shows the alignment of selected ESTs from the Hs.405444 UniGene cluster and of a rhesus-specific ACA62 snoRT with the human genome. The position of the U104 and ACA62 snoRNAs is also shown. The asterisks indicate the presence of a polyA tail in the EST sequences. The presumptive U104 ACA62 HG is larger than the UniGene cluster and overlaps the 3′ UTR of the TEX2 gene in the antisense orientation. The lower track shows the lack of conservation of the HG outside of the snoRNA sequences.

As shown next, the analysis of Type-3 snoRTs can also shed light on the structure of the parental HG. The human U99 snoRNA resides in an intron of the *C11orf48* gene in the antisense orientation, suggesting that it is produced from a transcription unit antisense to this gene [43]. The structure of a rhesus-specific U99 retroposon, which comprises the snoRNA followed by a 141-bp sequence, fully supports this hypothesis (Figure S3, section 14). Its alignment with the human genome shows that it corresponds to the 3′ region of several spliced ESTs, including BU564879, from the 5′ end of U99 to the end of the EST second exon (Figure 2A). These ESTs are thus representative of a new *U99HG* gene, but were incorrectly clustered with C11orf48 transcripts in the Hs.9061 UniGene cluster. The full-length intronless FLJ42151 mRNA appears as an unspliced transcript of this gene. The new (07-SEP-2006) RefSeq gene NM_001043229 (hypothetical protein LOC751071) corresponds to the *U99HG* gene (Figure 2A).

The existence of U99HG is further supported by the analysis of a previously described mouse U99 retroposon [43] that extends to the 3′ end of the mature snoRNA, but is preceded by 90 bp of upstream sequence from the parental copy (Figure S1, section 33). The retroposed sequence corresponds to the first 235 nts of the intronless 3.3-kb Riken 5730408K05 clone, suggesting that a similar transcript was polyadenylated and reverse-transcribed after the maturation of the 3′, but not the 5′, end of the snoRNA. Most probably, the mouse U99 snoRNA is normally processed from the intron of the AK011444 mRNA, which overlaps the Riken 5730408K05 clone in the same orientation, and the mouse orthologue (1810009A15Rik) of the human *C11orf48* gene in the antisense orientation. Therefore, the analysis of U99 retroposons in higher vertebrates supports the existence of the *U99HG* embedded in the *C11orf48* gene in the antisense orientation. As for other nonprotein-coding HG [44], the snoRNA sequence is better conserved than exonic sequences (Figure 2A). It is important to note that U99 is the only snoRNA that was found so far to be retroposed along with a substantial upstream sequence from the parental copy (Figure S1, sections 33 and 34). This might reflect a property unique to the way it was processed from its HG.

A 1436-bp rhesus-specific ACA62 retroposon located in the *NKIRAS1* gene (Figure S3, section 10) is particularly interesting, as the parental HG of this snoRNA is presently unknown. In vertebrate genomes ranging from human to opossum, the H/ACA box ACA62 snoRNA is located 177–225 bp downstream of the C/D box U104 snoRNA. The potential U104 HG, inferred from several human and mouse ESTs (UniGene cluster Hs.405444), contains two exons and a consensus polyadenylation signal, but also several intronless ESTs located downstream of the U104 HG (Figure 2B). The alignment of the rhesus ACA62 retroposon with the human genome shows that it extends further downstream of the UniGene cluster and overlaps the 3′ end of the *TEX2* gene in the opposite orientation. These observations indicate that the presumptive U104/ACA62 HG has a complex alternative splicing pattern and several polyadenylation signals. In this case, analysis of the rhesus retroposon suggests a position for the 3′ end of this new gene (Figure 2B).

### Relationships between snoRTs and Other Mobile Genetic Elements

Classical mobile genetic elements are frequently inserted in snoRTs, each insertion being characterized by its own TSD. As previously mentioned, several primate-specific U70 retroposons are interrupted by the insertion of an *Alu* element (Figure S2, section 3; and Figure S3, sections 2–4). Other examples include a full-length L1 element inserted in a mouse-specific Type-3 ACA48 retroposon in the 3′ UTR of the *Cenpc1* gene (see Figure S3, section 11). Conversely, interspecific genomic alignments show that several snoRTs were inserted inside of, and thus subsequently to, another mobile element (Figure S3, sections 15, 17, and 19), although the internal insertion could, in principle, have occurred before retroposition of a composite element. In two cases, a snoRT was inserted in a DNA transposon (Figure S3, section 9; and Figure S6). Such a series of consecutive events suggest that classical mobile elements and snoRTs can be inserted in similar favorable genomic environments.

However, other observations indicate that more precise targeting mechanisms might operate in some cases. A first argument is that a given genomic site occupied by a snoRT in one species can be occupied by different repetitive elements in other species (Figure S1, sections 34 and 37; and Figure S3, section 20). For example, the site occupied by a Type-1 U65 retroposon in the cow genome is occupied by a B2 element in the mouse genome, while the orthologous sites in human and rat genomes are devoid of repetitive sequence (Figure S1 section 14). In addition, the TSDs resulting from the insertion of a snoRT and an *Alu* or B2 element are in some cases adjacent (Figure S1, section 35), overlapping (Figure S1, sections 3 and 9; and Figure S3, section 16), or even identical, thus creating a target site triplication (Figures 1D and S1, sections 36 and 42). This feature is indicative of consecutive retroposition events at a common site, rather than a template-switching mechanism of the L1 RT that creates chimeric insertions bracketed by a single TSD [45,46]. One example of such an insertion containing a full-length RPS3A processed pseudogene and a U70 Type 3 snoRT is presented in Figure S3, section 24.

**Figure 3:**
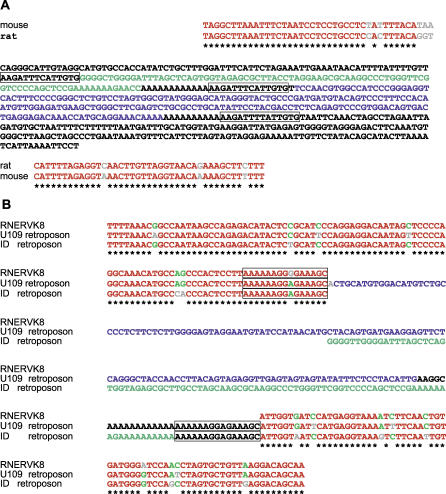
sno/scaRNA Retroposons and ID Elements Insert at Identical Sites in the Rat Genome (A) Rat-specific U17 retroposon and its corresponding insertion site in the mouse genome. This retroposon, located in an intron of the *Pdia3* gene in the sense orientation, is part of a rat-specific insertion containing a full-length ID sequence and the U17 snoRNA copy (green and blue letters, respectively), embedded in 90-bp and 113-bp sequences of unknown origin. The triplicated target site is boxed. Sequences shown are chr3:108128544–108129247 (rat) and chr2:121165874–121165955 (mouse). (B) Rat-specific U109 scaRT. This perfect copy of rat U109 is inserted in an extragenic region within an RnERVK8 repeat. Alignment with a typical RnERVK8 element precisely delineates the insertion site and the TSD (boxed). At a different genomic location, an ID element is inserted at the same position, with the same TSD. Note that the ID sequences from A and B display 90/93 nt identity. Sequences shown are: RnERVK8: chr12:18586394–18586560; U109 retroposon: chr7:118265612–118265946; and ID retroposon: chr8:18564189–18564463.

Finally, a snoRT and an ID sequence inserted at exactly the same site in two cases. In the first instance, a rat-specific insertion in the *Pdia3* gene contains an ID sequence and a U17 Type-1 retroposon in tandem with an almost-perfect triplication of the common insertion site (Figure 3A, and Figure S1, section 36). Therefore, the structure of this composite retroposon cannot be accounted for by a template-switching mechanism, but rather by two retroposition events at the same site.

A similar case was encountered with a rat-specific, perfect retrocopy of the U109 scaRNA located in an intergenic region of Chr7 (Figure 3B). Insertion occurred in a beta-retroviral RnERV sequence [47], and the alignment with an uninterrupted RnERV sequence delineated the insertion point and TSD. A BLAT search of the RnERV sequences encompassing the snoRNA insertion site in the rat genome resulted in ∼300 hits, one of which, located on Chr8, is interrupted by an ID sequence. Strikingly, the insertion sites and TSD are identical for both retroposons (Figure 3B). These two cases, as well as the target site triplications presented above, suggest that the machineries used for the retroposition of snoRNAs and other nonautonomous mobile elements can target identical genomic motifs.

### Previously Described snoRNA Genes Are Retroposons: Identification of the Parental Copy

Additional searches of vertebrate genomes for the orthologues of previously described snoRNA genes unexpectedly showed that some of them are in fact lineage-specific retroposons. The ACA63 snoRNA [48] resides in an intron of the *ATP2B4* gene. It is present in this gene only in primates, embedded in a 1027-bp insertion (Figure S3, section 16). However, a highly related snoRNA sequence, ACA63B, is present in the sense orientation in an intron of the *RANBP1* gene from human to chicken and fishes (see snoRNABase for alignment [http://www-snorna.biotoul.fr/snosync/phyl_img/ACA63.gif]). Moreover, the ACA63 retroposon in the *ATP2B4* gene includes 296 bp of the downstream intron and last exon from the *RANBP1* gene. This establishes that ACA63 is part of a Type-3 snoRT of the parental ACA63B snoRNA hosted by the *RANBP1* gene.

Similarly, the ACA67 snoRNA [48] is present in the *AF231919* mRNA gene as a Type-1 snoRT only in the human, chimpanzee, and rhesus monkey genomes (Figure S1, section 10). However, a very close paralogue, ACA67B, is found in the sense orientation in the first intron of the human *ODC1* gene (see snoRNABase for alignment [http://www-snorna.biotoul.fr/snosync/phyl_img/ACA67.gif]). The ACA67B snoRNA sequence is conserved in the *ODC1* gene in 11 vertebrate genomes and thus constitutes the parental copy of the ACA67 snoRNA gene. In addition, many ACA67B retrocopies were found in vertebrate genomes, including one in the *JTV1* gene that is primate-specific (Figure S1, section 41), suggesting that ACA67B is particularly prone to retroposition in various species. Moreover, the ACA42 snoRNA displays highly significant homology with ACA67B, and could originate from an ancient retroposition event (see snoRNABase for alignment [http://www-snorna.biotoul.fr/snosync/phyl_img/ACA67.gif]).

Other examples are shown for the U98b, ACA58, and ACA14a snoRNAs (Figure S1, sections 1, 12, and 39). In all cases, interspecific sequence searches and alignments (see snoRNABase) allow for the proper classification of retrocopies and the identification of the parental snoRNA gene, serving to elucidate the phylogeny and evolution of snoRNAs and their HG.

### snoRNA Genes Switch Host Genes by Retroposition

snoRTs located in an intron of a gene in the sense orientation give rise to new functional snoRNA genes, provided that the rRNA antisense sequences and structural elements such as the C/D or H/ACA boxes are conserved. In some cases, discussed next, mutations that almost certainly occurred after the retroposition event disable the parental snoRNA copy, resulting in a switch of the functional snoRNA to that residing in a new HG.

A first example is that of rodent U107 scaRNAs. In primates, three highly similar copies of U107 are located in the paralogous *MAGED2*, *TRO*, and *MAGED4* genes, respectively, on chrXp11.21–22. In the rat and mouse genomes, only the *Maged2* gene and the *Maged2* and *Tro* genes, respectively, are presently annotated. In these three cases, the corresponding U107 sequences are mutated in their ACA box (to ACG or ACT). The rat sequence has an additional mutation in its H box (to ACAGGC). Therefore, these U107 copies cannot be processed as functional snoRNAs. However, consensus U107 sequences are retained in snoRTs: U107 copies are present in the rat and mouse *Aven* gene (Table 1) and a rat-specific Type-1 snoRT is localized in the *Wtip* gene (Figure S1, section 6), both in the sense orientation. A similar phenomenon was observed in the dog genome, where functional U107 retrocopies reside in the *Ttc7b* (Table 1) and *Maml2* genes (Figure S3, section 8). The alignment of the U107 family members is presented at http://www-snorna.biotoul.fr/snosync/phyl_img/U107.gif.

A second example of the mutation of the parental snoRNA copy is that of the mouse ACA36 snoRNA. From human to tetraodon, ACA36 resides in the *DKC1* gene. However, the mouse snoRNA gene copy is disrupted by the insertion of two SINE elements (chrX:71,354,129–71,354,691). In this case, the ACA36 function is probably provided by a mouse-specific Type-1 retrocopy in the *Nans* gene (Figure S1, section 8). In these two examples, it thus appears that creation of a new functional snoRNA gene copy by retroposition could alleviate the selective pressure on the parental copy, where deleterious mutations caused by genetic drift or insertions of genetic mobile elements remained compatible with fitness.

## Discussion

I describe here three types of snoRTs characterized by a TSD and a short A-rich tail. They start at the 5′ end of the mature snoRNA sequence, but differ at their 3′end by the included portion of the parental gene. This additional part is reduced to 0–9 bp in Type-1 snoRTs, but includes most of the downstream intronic sequence in Type-2 snoRTs. Type-3 snoRTs include the entire downstream intron and the processed 3′ exons from the parental gene. Although four examples of Type-1 retroposons were previously reported [25,43], types 2 and 3 are characterized here for the first time. Several Type-3 snoRTs originating from ribosomal protein genes were previously annotated as processed pseudogenes, but their intronic parts (snoRNA sequence and downstream intron) were overlooked since the pseudogenes were identified by alignment of cDNA or peptide sequences with genomic sequences [49,50]. The Type-1 snoRTs I describe differ from small nuclear RNA U3 pseudogenes that arise from self-primed reverse transcription [51]. They also differ from retrogenes that contain U3 or U6 RNA sequences fused to an L1 or *Alu* element or to a processed mRNA and are produced by template switching of the L1 RT [45,46,52]. Although a snoRNA and another retroposon were frequently found adjacent to one another within a single species-specific insert, each possessed its own TSD, suggesting two successive retroposition events rather than co-integration by template switching. Several aspects of the processing and retroposition steps that are required to generate a new snoRNA copy are discussed below.

### Polyadenylation of snoRNA Precursors

For the three snoRT types described here, the 5′ end coincides with that of the mature snoRNA species, with the occurrence of a few untemplated nts, possibly resulting from a terminal transferase activity of the RT. With the sole exception of two U99 retroposons (Figure S1, sections 33 and 34), the retroposed snoRNA is thus fully matured at its 5′ end. However, the three types differ at their 3′ end. For Type 1, it corresponds to that of the mature snoRNA or of a precursor form with 1–9 additional bases. In cultured cells, maturation of the 5′ end of H/ACA snoRNAs from introns is rapid, but the trimming of the last nine 3′ nts takes about one hour [53]. This suggests that, during the last 3′ maturation step, snoRNA precursor forms are prone to a polyadenylation process. For Type-2 snoRTs, the polyadenylation site lies close to the intron branch point, suggesting that polyadenylation occurs shortly after splicing, although it might require prior release from the spliceosome, or lariat debranching. Indeed, the spliceosomal IBP160 protein couples C/D box snoRNP protein assembly to intron excision, showing intricate relationships between snoRNA maturation and splicing [54]. The generation of Type-3 snoRTs can be explained by endonucleolytic cleavage of the HG pre-mRNA followed by rapid exonucleolytic degradation up to the 5′ end of the snoRNA, while the 3′-most part of the pre-mRNA is normally spliced and polyadenylated.

Whereas the polyA tail of Type-3 retroposons is most probably synthesized by the conventional polyadenylation machinery of pre-mRNAs [55], a different complex might be operative for Type-1 and Type-2 retroposons. In the yeast, *Saccharomyces cerevisiae,* deletion of the Rrp6p nuclear exosome component results in the accumulation of polyadenylated forms of various RNAs, including rRNAs, the U4 small nuclear RNA, intergenic transcripts, and snoRNAs [56–59]. The recently identified TRAMP complex, composed of the Trf4p polyA polymerase, the Mtr4p RNA helicase, and the Air2p protein, polyadenylates RNA substrates and so stimulates the degradation of aberrant transcripts by the nuclear exosome [58,60]. However, 3′-extended forms of the U14 snoRNA accumulate in a *trf4*Δ strain, suggesting that this is also operative in the normal processing of snoRNA precursors [60]. One can therefore speculate that retroposed snoRNA species in vertebrates were polyadenylated by a TRAMP-like complex. Several observations support this hypothesis. First, the polyA-like tail of snoRTs often contains an important proportion of Gs, and the yeast TRAMP complex indeed displays significant, albeit reduced, in vitro polymerase activity with GTP rather than ATP as a substrate. In addition, the polyA-like tail of snoRTs is often composed of repetitions of short motifs, in agreement with the distributive, rather than processive, elongation mode of the TRAMP complex [60].

### Mechanisms of snoRNA Retroposition

Most snoRTs are bracketed by a TSD whose extremity coincides with the insertion site, as in the case of LINE and SINE insertions in cultured cells [61,62]. As for SINE and LINE elements, the size distribution of snoRT TSDs displays a 13–16-bp peak with a 9–12-bp shoulder (Figure S4A). In addition, the analysis of 5′ flanking nts indicates a ttAAAA consensus insertion point on the top strand (Figure S4B), corresponding to the L1 EN consensus cleavage site TTTT/aa on the lower strand [35,63]. Moreover, snoRNAs and classical retroposons were frequently found to insert at overlapping or even identical sites. This suggests that snoRTs, like *Alu*, B1, and B2 elements [14,64], use the LINE L1 machinery for their mobilization. The insertions of a snoRT and a SINE element at a common site, with target site triplication, evokes in a provocative manner the possibility that the L1 EN/RT can make the very same break twice to insert different elements. This could occur if the L1 machinery stayed in place after the first insertion, and then recruited a second RNA. Accordingly, the triplication case presented on Figure S3, section 8, where two TSDs are only separated by the AAAAAAATAAAA sequence, could be viewed as an abortive attempt to retropose a second RNA. Alternatively, the same site could have been recognized in two independent retroposition events. This hypothesis is supported by the independent integrations of a snoRT and an ID sequence at the same site, and with the same TSD, in an ERV sequence (Figure 3B), and implies that this site is somehow marked. Such hot spots might include a characteristic chromatin structure and/or, possibly, an altered conformation, such as DNA bends. Another possibility is that such a site binds specific proteins, which then bind to both TSDs after duplication, as for the integration of the yeast Ty3 retrotransposon at tRNA and other pol III genes. In that case, the Ty3 integrase interacts with the TFIIC–TFIIB complex bound at the target gene promoter [65,66]. The examination of dual retroposition sites in other genomes is required to determine whether the two events occurred simultaneously or not.

The mechanism whereby SINE elements hijack the L1 machinery is postulated to require their interaction with ribosomes [14,64]. How such a model can apply to snoRNA retroposition remains unclear as snoRNAs are strictly nuclear and their biogenesis does not involve a cytoplasmic step. This might explain why snoRNA retroposition is clearly inefficient, as assessed by the small number of snoRTs relative to SINE elements.

While certain sno/scaRNAs, such as U109, U70, and ACA67B, are nevertheless relatively successful, no snoRT could be found for others. Moreover, the number of retroposons for a given snoRNA can vary considerably among vertebrate genomes. There are about 150 ACA48-like sequences in the mouse and rat genomes but only 12 in human and rhesus monkey, nine in the cow, two in the dog and rabbit, and just one in the elephant and opossum. Subtle species-specific variations in snoRNA secondary structures or expression levels or co-mobilization with other repeat elements might explain their strikingly different retroposition levels.

### Retroposition Creates New Functional snoRNA Copies

snoRNA sequences inserted in a heterologous intron in the sense orientation are faithfully processed in cultured cells [27,53,67]. Therefore, the snoRTs that have similarly inserted in genes probably are functional copies, provided that their key structural and modification guide elements are preserved. The fact that several previously cloned snoRNAs turn out to be retroposons shows that this is indeed true. In these cases, a search for similar sequences in vertebrate genomes allowed for the identification of the parental copy. When the latter is disabled by mutations, like the mouse ACA36 snoRNA located in the *DCK1* gene, the retrocopy must take over the responsibilities of the parental gene.

Assuming that the parental copy remains functional, a snoRNA retrocopy could also diverge during evolution. In particular, mutations in the PU pockets of H/ACA snoRNAs might change their RNA target specificity and even create a new rRNA modification guiding function. As a case in point, the E2 and ACA6 snoRNAs display a high overall sequence homology. For both, the 3′ PU pocket guides the PU of 28S rRNA U3832, but their 5′ pockets guide the modifications of the U3616 and U3830 28S rRNAs for E2 and ACA6, respectively. From the alignment of vertebrate ACA6 and E2 sequences, subtle sequence variations can explain this target switch (see alignment on snoRNABaseat http://www-snorna.biotoul.fr/snosync/phyl_img/ACA6.gif). As the ACA6, but not the E2, snoRNA could be tracked back to the zebrafish and fugu genomes, one can hypothesize that E2 was generated from an ancient ACA6 snoRT. Similarly, ACA62 might be an ancient copy of ACA50 (see alignment at http://www-snorna.biotoul.fr/snosync/phyl_img/ACA50.gif).

In summary, I have described a new class of vertebrate retroposons that constitute a previously unsuspected family of mobile genetic elements. While snoRTs targeted to extragenic regions or in genes in the antisense orientation are “dead on arrival,” those located in introns in the sense orientation can be processed into functional snoRNAs and become potentially subject to new retroposition events. Over evolutionary time, such a dynamic mobilization of snoRNA copies might have two general consequences. One is the maintenance of intact genomic copies that preserve essential-modification guiding functions, and, through the generation of retrocopies, protect from the consequences of deleterious mutations arising from genetic drift and/or insertion of genetic mobile elements. A corollary of the latter is the possibility for snoRNA sequences to evolve more freely and possibly capture new RNA targets. An exhaustive and complete search for snoRNAs and their retrocopies in sequenced vertebrate genomes constitutes a daunting task that is not complete as of now. Results presented here serve to demonstrate the existence of sometimes ancient snoRNA retroposition events that can be extended to more recent mobilizations and suggest probable mechanisms for their generation. They also establish the identity of snoRTs as a new member of the vast and diverse family of mammalian mobile genetic elements. As such, these observations must be considered as part of ongoing work, the results of which will continue to be incorporated into the snoRNABase, thus providing a useful repertory of retroposition events that impact the evolution of snoRNA-mediated editing functions as well as genome evolution.

Indeed, in addition to its presumptive role in generating guides for new RNA modifications, snoRNA retroposition might provide a new mechanism for insertional mutagenesis. Furthermore, Type 3 snoRTs inserted into an intron might, as L1 elements, participate in exon shuffling [41,42], but also disrupt transcription by premature termination at the inserted polyadenylation site [68]. A major difference between snoRTs and L1 elements is that the latter carry their own sense and antisense 5′ UTR promoters, and thus drive the transcription of many human genes [69,70]. In contrast, snoRTs can only be transcribed when inserted in a gene.

Although the number of retroposons for an individual snoRNA is relatively small, the number of different snoRNAs (∼380 are presently annotated in the human genome) suggests that the number of retroposition-active snoRNAs is comparable to that of active *Alu* or L1 elements. This raises the possibility that snoRTs might be polymorphic in the human genome, a question that will be addressed when their complete list becomes available. Due to their higher sequence diversity compared with classical LINE and SINE elements, they constitute new valuable markers for the study of vertebrate genome evolution.

## Materials and Methods

The sequences of human snoRNAs were retrieved from the snoRNABase [33]. Online BLAT searches on the UCSC Genome Browser site [34,71,72] were used to identify similar sequences in 17 vertebrate genomes. For a given species, a snoRNA orthologue hit was identified by BLATing the protein sequence of the human HG, and/or by the examination of Alignment Net tracks [49,73]. Other hits were examined for the presence of a polyA tail and TSD. Alignments provided by the Alignment Net tracks of the University of California Santa Cruz Genome Browser (http://genome.ucsc.edu/ and http://hgwdev-fanhsu.cse.ucsc.edu) were used to localize the retroposon insertion sites in the genome of other species. Alternatively, genomic segments were aligned using the Yass (http://bioinfo.lif1.fr/yass/yass.php) [74,75] and MultAlin (http://www-archbac.u-psud.fr/genomics/multalin.html) [76] online programs. Repetitive elements were analyzed online with RepeatMasker (http://repeatmasker.genome.washington.edu/ and http://woody.embl-heidelberg.de/repeatmask) (A. F. A. Smit, R. Hubley, and P. Green, unpublished data). Sequences of repeat elements were retrieved from the Repbase Update (http://www.girinst.org/repbase/update/index.html) [77]. Logo representation of base frequency was created with weblogo (http://weblogo.berkeley.edu/logo.cgi).

## Supporting Information

Figure S1 Examples of Species- or Lineage-Specific Type 1 snoRTs Insertion sites were determined by interspecies genomic alignments, only parts of which are presented. Sequences of the snoRNA copy are in blue letters, with additional 3′ sequences of parental origin in red. The snoRT TSDs and the snoRNA ACA motif (always located three nts upstream of the 3′ end of the mature form) are boxed. Genomic coordinates given are for the sequences shown, not the retroposon itself, and thus encompass a larger segment than those given in Table 1.
(284 KB DOC)

Figure S2 Examples of Species- or Lineage-Specific Type 2 snoRTs The site of insertion was determined by interspecies genomic alignments, only parts of which are presented. Sequences of the snoRNA copy and of the downstream intron from the parental gene are in blue and green letters, respectively. The retroposon TSDs and the snoRNA ACA motif (always located three nts upstream of the 3′ end of the mature form) are boxed. Genomic coordinates given are for the sequences shown, not the retroposon itself, and thus encompass a larger segment than those given in Table 2.
(55 KB DOC)

Figure S3 Examples of Species-or Lineage-Specific Type 3 snoRTs The insertion site was determined by interspecies genomic alignments, only parts of which are presented. In the retroposon, sequences of the snoRNA copy and of the downstream intron from the parental gene are in red and black letters, respectively. The sequences of the processed downstream exons are in uppercase blue letters, with asterisks indicating exon–exon junctions. The retroposon TSDs and the snoRNA ACA motif are boxed. Genomic coordinates given are for the sequences shown, not the retroposon itself, and thus encompass a larger segment than those given in Table 3.
(179 KB DOC)

Figure S4 Analysis of snoRT TSDs (A) Size distribution of 83 TSDs. A zero size means that no TSD was evidenced.
(B) Analysis of the EN cutting site. The logo represents the nucleotide composition of the first seven bps of the 5′ TSD and four flanking bps.
(378 KB AI).

Figure S5 Overlaps of U70 Type 3 snoRTs and ESTs (A) U70 snoRT overlapping the first exon of the DR731413 EST. This exon shares the splicing donor site of the second exon of the CD367579 EST, and could thus be an alternative, longer form of the same exon of an unidentified transcription unit. Note that this exon encompasses the snoRT insertion site, as shown by the Dog Alignment Net track. See sequence in Figure S3, section 21.
(B) A U70 snoRT provides the second exon of the CF130057 EST, in the opposite orientation. Note that the EST second exon is entirely included in the primate-specific insert. See sequence in Figure S3, section 23.
(863 KB AI).

Figure S6 Insertions of snoRTs in DNA Transposons (A) Cow-specific U17C retroposon inserted in a *mariner* element. Blue boxes in the upper part represent orthologous segments of the dog and cow genomes. The lower part shows a scheme of the cow-specific insert, composed of an *Oamar1* DNA transposon (black box), in which the U17C Type 3 retroposon, a CHR-2A element (tRNA-glu family), and a 44-bp retroposon of unknown category (X) are inserted (Figure S3, section 9). Each of these three retroposons is bracketed by its own TSD. Arrowheads indicate the orientation of the inserts. The *mariner* element deleted of these three insertions displays highly significant homologies (E value 7e-92–1e-60) with a *mariner* element that is present in the 3′ UTR of the prion–protein gene in the cow and mule deer and has been previously described in the sheep genome [78]. A highly significant homology (E value 7e-62) also exists with a *mariner* element from the insect Chymomyza amoena [79]. The genomic segments shown are: chr19:44,121,592–44,124,353 (cow) and chrUn:11,960,202–11,960,385 (dog).
(B) Human-specific U3 retroposon inserted in a *Charlie* element. The upper drawing shows the orthologous segment in the rhesus genome, blue boxes represent sequences that align with the human genome. The lower drawing shows that, in the human genome, the rhesus *AluSx* element is replaced by a 8.4-kb segment containing a L1 element (yellow box), two *AluSx* elements (green boxes), and a *Charlie1A* element (black box) in which the U3 retroposon (red box) and two additional *AluSx* elements are inserted. The corresponding segment in the current chimpanzee genome assembly contains large gaps. The genomic segments shown are: chrX:69,748,687–69,750,394 (rhesus) and chrX:69,974,000–69,983,999 (human).
In (A) and (B), the various elements are not drawn to scale.
(167 KB AI).

Table S1 Analysis of snoRT TSDs The sequence of 5′ TSD is given in capital letters, with the four flanking nts in small letters. NS, snoRTs not shown in Tables S1–S3.
(94 KB DOC)

### Accession numbers

The Genelynx database (http://www.genelynx.org/cgi-bin/a?page=home) accession numbers for the genes and proteins discussed in this paper are *AAK1* (20549), *ASTN2* (86), DKC1 (7629), fibrillarin (5441), GAR1 (NOLA1) (13007), *JTV1* (22389), *NHP2* (NOLA2) (10902), NHP2L1 (8272), NOP5/NOP58 (10031), NOP10 (NOLA3) (12004), NOP56 (NOL5A) (24738), and UCHL5 (119798).

The snoRNABase database (http://www-snorna.biotoul.fr/index.php) accession numbers and the HUGO Gene Nomenclature Committee (http://www.gene.ucl.ac.uk/nomenclature/)-approved symbols for the snoRNAs discussed in this paper are ACA6 (SR0000304, SNORA6), ACA14a (R0000351, SNORA14A), ACA36 (SR0000374, SNORA36A), ACA42(SR0000024, SNORA42), ACA48 (SR0000228, SNORA48), ACA50 (SR0000222, SNORA50), ACA58 (SR0000309, SNORA58), ACA62 (SR0000249, SNORA76), ACA63 (SR0000037, SNORA77), ACA67 (SR0000299, SONRA80), E2 (SR0000305, SNORA62), HBII-95 (SR0000277, SNORD11), HBII-234 (SR0000276, SNORD70), U17A (SR0000003, SNORA73A), U17B (SR0000004, SNORA73B), U65 (SR0000358, SNORA65), U69 (SR0000371, SNORA69), U70 (SR0000373, SNORA70), U98b (SR0000038, SNORA16B), U99 (SR0000053, SNORA57), U104 (SR0000248, SNORD104), U107 (SR0000368, SNORA11), and U109 (SR0000324, SCARNA18).

The Genecards database (http://www.genecards.org/index.shtml) accession numbers for the genes and proteins discussed in this paper are *ATP2B4* (GC01P200327), *Maml2* (GC11M095351), *NKIRAS1* (GC03M023908), *ODC1* (GC02M010531), *RANBP1*(GC22P018479), *RPL10* (GC0XP153147), *TEX2* (GC17M059579), and *TTC7B* (GC14M090076).

The Entrez database (http://www.ncbi.nlm.nih.gov/gquery/gquery.fcgi?itool=toolbar) accession numbers for the genes, gene clusters, and proteins discussed in this paper are *Aven* (NM_028844), *Cenpc1* (NM_007683), *C11orf48* (NM_024099), hypothetical protein LOC751071 (NM_001043229), mouse *Maged2* (NM_030700), rat *Maged2* (NM_080479), *Pdia3* (NM_017319), *Rpl39* (NM_012875), SNORA76 (Hs.405444), LOC751071 (NM_001043229) and *Wtip* (NM_207212).

Coordinates given are from the following genome assemblies: human (hg18, NCBI Build 36.1, March 2006), chimpanzee (panTro1, NCBI Build 1, November 2003), rhesus (rheMac2, January 2006), dog (canFam2, May 2005), cow (bosTau2, March 2005), mouse (mm8, NCBI Build 36, February 2006), and rat (rn4, Baylor HGSC version 3.4, November 2004). 

## Abbreviations

bp: base pairs
EN: endonuclease
EST: expressed sequence tag
HG: host gene
LINE: long interspersed element
nts: nucleotides
PU: pseudouridylation
RT: reverse transcriptase
scaRNA: small Cajal body–specific RNA
scaRT: scaRNA retroposon
SINE: short interspersed element
snoRNA: small nucleolar RNA
snoRNP: small nucleolar ribonucleoparticles
snoRT: snoRNA retroposon
TSD: target site duplication
