# Correction: Mammalian Small Nucleolar RNAs Are Mobile Genetic Elements

**DOI:** 10.1371/journal.pgen.0030036

**Published:** 2007-02-23

**Authors:** Michel J Weber

In *PLoS Genetics,* volume 2, issue 12: doi:10.1371/journal.pgen.0020205

In the Materials and Methods, the URL to the Yass online program was incorrectly listed. The correct URL is http://bioinfo.lifl.fr/yass/yass.php.

