# Supplementary material for: Mammalian Small Nucleolar RNAs Are Mobile Genetic Elements
Source: PLoS Genet. 2006 Dec 8;2(12):e205. doi: 10.1371/journal.pgen.0020205 (PMC1687206; doi:10.1371/journal.pgen.0020205)
Supplement: Supplementary file 2 [file pgen.0020205.sg005.pdf]

A

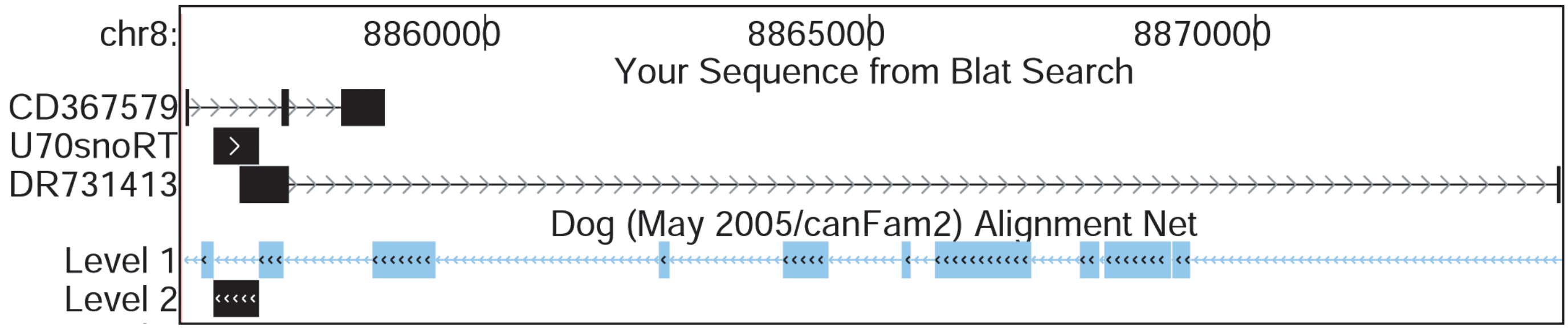

B

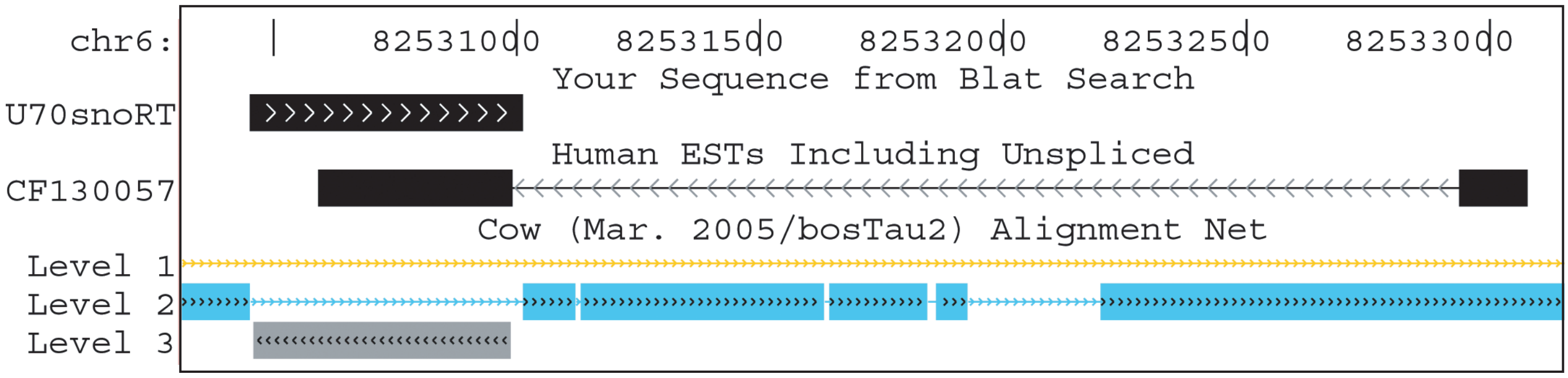

Supplementary Figure S5
