# Supplementary material for: Mammalian Small Nucleolar RNAs Are Mobile Genetic Elements
Source: PLoS Genet. 2006 Dec 8;2(12):e205. doi: 10.1371/journal.pgen.0020205 (PMC1687206; doi:10.1371/journal.pgen.0020205)
Supplement: Supplementary file 4 [file pgen.0020205.sg001.doc]

**1- U98b is a primate-specific ACA16 snoRT in the PPP2R5A gene (sense); parental gene PNAS-123**

```
dog     GTATAACAAATTATCACAAAATTTAGTGGCTTAAAACAATAAACTATTA
rhesus  GCATATCAAATGATCACAAAATTTATTGGCTTAAAATAGCAAGCATTTA
        * *** ***** ************* ********** *  ** *  ***

tggcctttatcaaagctgcagctgcttccatgtagctgctgtggtcaaaaagaagccgagagtgacagttttccgtgatggtcacagttctgtttgctgtaactgatctgcaacattttgggaaaatatagttccattgggaggaaaaataacaagcatttg

rhesus TTGCCTTAGTTGCTGTGAATCAAGAATCTGGTCATGGCTTAGGTGGGCTCTCTGCTTC AGGGT
dog    TTATCTTATT  CTGTGAAGCAGGAATCCAGGGATTACTTAGCTGGGTCCTCTGCTTTTAGGGT
       **  **** *  ******* ** *****  *  **  ***** ****  ********  *****
```

This retroposon corresponds to the U98b snoRNA. The parental copy was cloned as ACA16. The snoRNA is retroposed with 6 bp of downstream sequence from the parental gene.

rhesus : chr1:158,051,281-158,051,554

dog : chr7:13,519,347-13,519,457

```
2- Primate-specific ACA16 snoRT in the REPS2 gene (anti-sense); parental gene: PNAS-123

dog    TCTGTCCCACCACCACCCTGCGAATGAAAGCACGAAAATCTCTGTGCTGA
rhesus TCTGTCCCACCACTTCCCAGTGAATGAAGACACAGAAATCTCCATGCTGG
       *************  *** * *******  ***  *******  *****

gcggctctcatcgcagctgcagctgcttccacatagctgctgtggtcaaaaagaagcccagagtcagttcccttgaccatcaccattctgtttgctataactgatctgtaacattttgggaaaatacagttccattgaaaagaaaaagagaaaaaatctccaagctgg

rhesus ATGTTGGGTCTGCTGCATTCCTGGGTCTGACATGGAAGCTGCTGCTCCCCTAGAA
dog    GTGTTGG  CTACTGCATTCCCGGTTCTGACACAGAAGCTACTATTCCCCTAGAA
        ******  ** ********* ** *******  ****** **  **********
```

The snoRNA is retroposed with 6 bp of downstream sequence from the parental gene (underlined red letters), and two 5’ untemplated nts. Note the differences in the snoRNA sequence with that presented above.

rhesus: chrX:14,722,379-14,722,651

dog: chrX:13,150,437-13,150,539

**3- Primate-specific ACA30 snoRT in the PGS1 gene (sense); parental gene:SRCAP**

**dog	GGCAGAGCCAGGAACACAGAAGAAATGAACCGCAGAGCCAGGA TGGCCGCCTGGGTCCTTTTGATTCTGAAACCTGGGCACTGG**

**human GGTAGA CAG AGC CAGAA AAATAAACA AGAGTCAGACCTGGAAGTCCAGGTCCTTAGG	CTGATAAATA**

**** *** *** * * ***** **** ***	**** ***	*** * * ******* *	**** * ***

tgggccctttcacaggccctcccctcggcagtggggccaggattgggttgctggtgctgagagaaagcccttgattgcatccttgctctgggactgtaccagtggcagctgtcacttagtgggacagagagaacaaacaaacatgggcgctgataaatagccctgcatgggccgggcgcagtggctcacgcctgtaatcccacattttgggaggctgaggcagagggatcacctgacgttagaagttcaagaccagcctggacaacatggtgaagccccgtctctactaaaaatacaaaaattagctgggtgtggtggcacacacctgtaatcccagctactcgggaggctgaggcaggaggatcacttgaacctgggaggcggaggctgcagggagccaagatcatgccactgcactccagcctgggcgacagaatgagattgtctcaaaaaaaa

**human AAAAAGCCCTGTGTGGTTAGTTGACTTTCTGTGTCTGCATCTCCTCTTCTCAGCTACACTGTCGGCTCCTTGAGAGGAGGAGGCTG**

**dog	GCTCAGCCCTG GTGGCGGGTAGT TGGCTTTGTGAGGGTC CCTCTTCTA	TCAGCTGTCGCCTCA GCATCGAG ACTCCG**

********* ****	** * * ** *** * ** ********	*	****** ***	*	*** * * ***

This primate-specific insert contains a Type-1 ACA30 snoRT (blue, TSD boxed) and an *AluSx* element (TSD highlighted in yellow). Note the overlap of the TSDs. The snoRT was previously described by Bachellerie et al. (2002) as the HBI-26 retrogene.

human: chr17:73,907,544-73,908,168

dog: chr9:5,827,409-5,827,584

```
4- Rat-specific ACA48 snoRT in the Nap65 (human NOP68) gene (sense); parental gene: Eif4a1

mouse ATGCTGCATAAGAAAACTAGCTTATTTCAATAAGAGAAAAACTTTTTCCTCTTATTTTATGTGAATAGTTGTTA ACTTGAAAAATA
rat   ATGCTCTGTAAGAAAACTAGCTAATTTCAATAAGAGAAACATTTTCTCCCCATATTTTATGTTAATAGTTGTTTGAGTTGAAAAATA
      *****   ************** **************** * *** *** * ********** **********  * **********

tagttgtcgccccctggcaatgggcaaagagacaccagatttgtggtgttgttcttacatcagtcaggggcaaagcagatggccgtcctttgttcatcctagcttgatgcctggctcggtggccaaagctatttcatgcctagggcacataaaggaaggaaggaaggaaggaaggaagaaata

rat   GTTGTATATGTCTAAACTGCCTTTGAAGAAAACTGGTTTATTGCAGGTTCTGAATGAGAAGAAACTTCAAGAG
mouse GTTGTATATGTCTAAATTGCTTTTGAAAAAAACTGGTTTATTGCAGGTTCTGAATGAGAAGAAACTTCAAGAG
      **************** *** ****** *********************************************
```

The snoRT contains 8 5’ untemplated nts. The blue sequence in the downstream alignment corresponds to the 5’ portion of the Nap65 second exon. In the rat as in other mammalian species, the NOP58 gene is also the host for the HBII-234 and HBII-95 C/D box snoRNAs.

rat: chr9:58,405,880-58,406,222

mouse: chr1:59,634,900-59,635,058

**5- Rat-specific ACA48 snoRT in the ZZEF1 gene; parental gene: Eif4a1**

**mouse AGCACTGTTCAGTTTGAATATCTACACAGGC**

**rat	AACACTGTTGAGTTTAAAAATCTACACAGCG**

*** ******* ***** ** ************

ccccctggcagtggacaaggagacaccatgttggtggtggtgttcttatatcagtcacagacagagcagatggcaggccttgcttcatcctagcttgatgcctggcttggtggccaaagctgtttcatgcctagggcacatgaaaaaaaaaagaagaaaagaagaaagaaagaaagaaagaaagaaagaaagaaagaaagaaagaaagtaagtctacacaggc

**rat	TTCTTTCATCTGAAAGACTTAATTTGCTAGTCCTGTACCTTGCCTGATTTGAAT**

**mouse TTCTCTCATCTGTAAGACTTA TTTGCCAGCCTTGCACTTTGCCTGATTTGAAT**

****** ******* ******** ***** ** * ** ** *****************

Note the AAAG repeats in the polyA-like tail.

rat: chr10:59,745,896-59,746,203

mouse: chr11:72,656,573-72,656,656

```
6- Rat-specific U107 snoRT in the Wtip gene; parental gene Maged2

mouse TGGTCCCCTGTCCCATCCAGTATCCCCAGATCCCT     GCATAGAAAGTACTGTCCTT
rat   TGGTCCCCAGTTCCATCCAGCATCCCTGGATCCCTACATAGCATAGAAAGTACTGTTCTT
      ******** ** ******** *****  *******     **************** ***

GGGGTGTGCTCAGAGCAGAGGGCCTGAAGAATGGCTTCTCTGTTTACAACACACCCAAGAAGAATCTGGGACCATTGAAATGTGGGGCACAAGCAGTTTCCTCTCTGTGTGTGGATCCCCTACATGTgtcaaaaaaaaaaaagtcctgttctt

rat   TCAGCTCATGGTGTAAAGACC                      CCAGTGTAGAAATCACA
mouse TCAGCTCATGGTGTGAAGACTACAGAGGATCCTCTCTCACCATCCTGTGTAAAAATCACA
      ************** *****                       ** ***** ********
```

A mutation in the ACA box (to ACG) invalidates the parental copy in the rat Maged2 gene. This snoRT probably encodes a functional copy.

rat: chr1:86,666,658-86,666,908

mouse: chr7:33,835,744-33,835,858

**7- Rat-specific ACA6 snoRT (extragenic); parental gene : RPSA**

```
mouse TCTGTATCCCAAATTCTTTCTCAACAACCATTAGTCATCAGAGTTGTTATAAATGTTTGTTTAC
rat   TCTGTATCC TGATTCTTTCTCAACAGCCACTAGTTGCCAGAGTTGCTACAAATGTTTGTTTCC
      *********   ************** *** ****   ******** ** ************ *

TGCACACTATTAAAGCTCAGGGTGGAGGCCAGtcTTGGCCAGTGAACTTCTGcaTGTCaGAAGTGTGCTACATTGAACcTAtGGCAGGATTTTCGCTAACACtAGTAGAGCTTGCCTCTATGACTGGAGTTTGGTAGTGGTCACTGaCACAcACacacacacacacacacacacacacacaccccaaatgtttgtttac

rat   TTAACCATTTTAGAGTATAGAAGACCCATAAATTACTATTTATAGCTGTTGAACACTAAA TCATAATGATAATTCTTGAGAGGAG
mouse TTAACCATTTTAGAGTATAGAAGACTCACAACTTACTATTTATAGCTGTTGAACACTAAA TCATAATAATAATTCTTGAGAGGAG
      ************************* ** ** **************************** ******* *****************

Note that the polyA-like tail is composed of an AC repeat.
```

rat: chr4:174,347,760-174,348,106

mouse: chr6:137,003,042-137,003,190

```
8- Mouse-specific ACA36 snoRT in the Nans gene (sense); parental gene: Dkc1

rat    GGGTAAAAAGGATATCA AAAAAGTGAAAAGAC
mouse  GGGTAAAAAGCGAACCAGAAAAAGTGAAAAGAC
       **********   * ** ***************

ctcccaaagtgttgagttcagttcagggcagcttccctgttctgttaattaaactttgggacattggaattggctgagggaaatgactgaatagagagcattactctattcatattcttctagcctacacaaacaaataaaatacagttttaaaaagttaaaaaaaaagagtgaaaagac

mouse TAATAGTCCCAGCAGTTGGGAGGTGG
rat   TAATAGTCCCAGCAGTTGGGAGGTGG
      **************************

The parental copy in the mouse DKC1 gene is invalidated by the insertion of B1 and B2 repeat elements.
Mouse: chr4:46,516,221-46,516,459
Rat: chr5:63,080,329-63,080,386
```

**9- Mouse-specific ACA48 snoRT in the Sec24a gene (sense); parental gene Eif4a1**

**rat	TCACATAAGTAAGTAAATCTAAAAATTCAGATTTTTGTGC**

**mouse TCACATAAATAAGTAAATCTTAAAAGTTAGATTGTGGGGC**

********** *********** **** * ***** * * ****

tggtgagatggctcagtgggtaagagcacccgactgcttttccgaaggtccgaagttcaaatcccagcaaccacatggtggctcacaaccacccgtaatgagatctgactccctcttctggagtgtctgaagacagctacagtgtacttacatataataaataaataaatctaaaaaaaaaaaaaaaaaaaaagttagattgttcacccctggcaatgggcaaagagacaccttgtttgtggtgttgatcttatatcagtcaggggcagagcagatggcagtcctttgttcatcctagcttgatgcctggcttggtggccaaagctgtttcatgcctagggcacattaaaaaaattttttttagattgttgtgc

**mouse ATGAGTGTTTTGCCTGCATCTATGTAAGTGCACTATATGCATGCCTTGTGCCATGCCAAA**

**rat	ATGAATGTTTTGCCTGCATCTATGTAAGCTCACTGTATGCATGGTTTATGGCATGCCAGA**

****** *********************** **** ******** ** ** ******* ***

This mouse-specific insert is a type 1 ACA48 (TSD boxed) in which a B2 element (underlined) inserted with an overlapping TSD (highlighted in yellow).

mouse: chr11:51,581,116-51,581,589

rat: chr10:37,263,842-37,263,941

**10- ACA67 is a primate-specific snoRT in the C21orf108 gene (AF2319919 mRNA, sense); parental gene: ODC1**

```
dog    GACAGGGCTGCACTTCAATTGG TGTTT TGCAAGGCATTTAGGCCAGTATGCATCAGAACTTTAAACACATTC
rhesus GACTGGAACGTGCTACAGTTTAATGTTTCTGCTGGGAGTTTGG  CTGTACGCATTAAAACTTTTAAGATGCTT
       *** **   *  ** ** **   ***** ***  **  *** *  * *** **** * ****** ** *   *

atgcatgggtttggatttatgatgggcccgtccccctgggcctctcatagtaccccatgccagagcaaactgtagccccgaaccattgcctggcctctgtgcccgtaggctgctggcactgaagtgggttgcacaatggataaagaaaaaaagataaaacaacttttaagatactt

rhesus GATCCTGCAATTCCATGTCTAGTCATTTATCCCAGTGAAATAATTACAGATGTGAGTAGAAGTTTTGTTAGGGAATAATA
dog    AATCTGGCCTTTCCATGTCTAGTCATTTATCCTAAGGGAAGAATTAAGGATGTGAGTAGAGGTTTTCTTAGGGGATATTT
        ***  **  ********************** *  * ** *****  ************ ***** ****** *** *
```

The ACA67 snoRNA described by Schattner et al. (2006) is a Type 1 snoRT. The parental copy resides in the ODC1 gene. Red nts in the snoRT sequence are of parental origin.

rhesus: chr3:14,131,970-14,132,297

dog: chr31:30,162,842-30,162,993

**11- Rat-specific ACA42 snoRT (extragenic)**

**mouse CCAGCCTCTGCGGGAAACCTGGAGGCTGACTTTGGTGGGCTCCTTGCCCCGAGCTATAAAGAAAATCC**

**rat	CCAGCCTCTGCGGGAAACCAGGAGGCTAACTTTGGTGGGCTCCTTGCTCCAAGTTATAAGGAAAATCT**

********************* ******* ******************* ** ** ***** *********

catgcgtggatttggatttatggcaggctcatccccatgggcctctcatagtgtcccatgctagagcaaactgtggctcctaaccattgcccggcctccgtgcatgtagactgctgcactgaagtgggtcacacagtgggaaaaaaaagaaagaaagagagaggggggaggaaaggaaagaaaaagaaaagagaagaaaagaaaagagaagaaaagaaaatct

**rat	TACCCTAAGGAAATGAGAACACTTTCACTCTGGATCAAGCCAGTTATCATACACAGAGGCCTGTAATCTGCCTCTCCATCCCTGTC**

**mouse CATCTAAAGGAATTGAGAACACTTTTGCTCTGGATCAAGCCAGTTGTCATAAACAGAGGCCTGCAATCTGC TCCCCATCCCTGTC**

*** * ****** ************ ****************** ***** *********** ******* ** *************

The parental copy is presumably present in a gap of the expected intron of the rat gene orthologous to the human KIAA0907 gene (rat BC092205 mRNA).

rat: chr18:63,609,476-63,609,852

mouse: chr18:67,138,768-67,138,920

**12- ACA58 in the MRPL3 gene is a primate-specific Type-1 snoRT; parental gene: UBAPL2**

```
dog   TATGTACAAGTGACTTGAACTTTTTACAGCTTTAATAAATCAGCTAAAGAGAGAACATTAAAGCTCAATTTAGA
chimp TATGTACAAGTCACTTAAACTTCTTACAGCAT             AAAGAGAGAAAGTGAAAACTTAATTTGGA
      *********** **** ***** ******* *             **********  * *** ** ***** **

gggcatactcgtagaccttgcctgactgtgctcatgtccaggcaggggggacagtgtatgcaagaataatttggagttcctgccagctctaaccagcttcatcagtggctggataaattgcaggactctaaacgtttccctgaagaaaaaaagaaaacttaatttgga

chimp AATGGTTCTAATTGAAAAACCTCTACTTAGAGTATGGATTTT
dog   AATGGTTCTGATACAAAAATCTCTACTTGCAGTATGGATTCT
      ********* **  ***** ********  ********** *
```

The parental copy of this snoRT resides in the UBAP2L gene in 11 vertebrate species. Note that the consensus ACA box in the orthologous human and rhesus snoRTs is changed to ACG in the chimpanzee.

chimp : chr2:134,772,049-134,772,319

dog : chr23:31,632,925-31,633,040

**13- Primate-specific ACA58 type 1 snoRT in the TMEM48 gene; parental gene: UBAPL2**

```
Dog   GCATGAAATATG   TGTATTGATAATTGAGCGAAAGCAGAGAGAA
Human GAAATAGATATGACATATATTAATGATAGAACAAAAGCAAGGAGAA
      * *  * *****   * **** ** ** ** * ******  *****

gggcatacccgtagaccttgcctgactgtgctcatgtccaggcaggggggacattgtattcgagattaatttgaagttcctgccagctttatccagcttaatcagtggctggataaatagcaggactgtaacattcccctgggggaaaaaaggcaagaagaa

Human AAGGGGAGCATAGGGATTTAGATAAGAAAGCGATATA
Dog   AAGGGGA CACAGAGTTTTAGATAAGAAAGGGGGATA
      ******* ** ** * ************** *  ***
```

This snoRT is present in the human and chimpanzee TMEM48 gene. The orthologous rhesus gene could not be identified.

human: chr1:54,009,216-54,009,460

dog: chr5:58,205,884-58,205,962

**14- Cow-specific U65 type 1 snoRT; parental gene: RPL12**

```
human TGCTCTTTGGGGTGTGATGGTGAGGATCTATGTCACAGAGAGCCTCTGTCAGATTGGGTGTTTGAGGCTCTTGGTTTCTA
cow   TGCTTTACAGGGGCACCATGGGATGACCTATGTCA    GAGTATGTGTCA CCTGGGAAGTTGAGGCTCTTGGTTTCTG
rat   TGCTCTGTGGGGGCATGGTGTG TGAGTTTTGCCACTGTAGCTGTATGTCTGATCTAGGATTTGGGGACCTAGATTTCTA
mouse TGCTCTGTTGGGGCATCGAATG TGAGTTGTGTGTCTGTAGCTGTATGTCTGGCCTGGGATTTGAGGGTCTAGATTTCTA
      **** *   ***         *  **  * **            * ****       *   *** **  ** * *****
human GGCAATTTCTCTATGAAATTTTATTCTGTTTATTT          GAATTCAAGG GTCTTTTATATCTTTAAAAA TAT
cow   GGCTGTTTCTTTAAGAATTCTTGTTCTGTGGATTTTCATGTTGTAGAATTCAAGGAGTCTTTTGTAT   TTAAAA TAT
rat   TGTAAATTTCCTATAAAATTTTGTCCCATTTACTGTCATGTTTCAAGACTCAAAGGGCCTTTTCAATCTTTTTTTT AAT
mouse TATAATTTTCCTATAGAATTTTATCCTATGTACTGTCATTTTTCAAGACTCAAAGGGCCTTTCTAATCTTTTTTTTTAAT
            **   **   * * ** * *  *  * *             * **** * * ****   **   *       **
human GTAGAGTTTCTCCCTGCATTTTATTGCATTAGAAAGAAAATAGACTGATGGCATGTACTATTTTAAAGATTACTCTTAAA
cow   GCAATGACTTATCCTGTATTTCATTGTTTTAGAAAGA       CTGATGGCACGTACTATTTTAA GATTACTCCTTTA
rat   GTGATATTTTTATTTT ATCTTATTGTTATAGAAAGAAAATAGACTGGCCATATGCACTACTTCAA GATTGTTTGTTTG
mouse GTGATGTTTTTGCTTTCATTTTATTGTTATAGAAAGAAAATAGACTGGTGGTATGCACTACTTCAA GATTGTTTGTTTG
      *       *     *  ** * ****   ********       ***     * * **** ** ** ****  *  *

human AAAAAAAAACAACAAACCTCTTGTCTTTTGAGGAGGGGATGTCTCTTTAAAAACAAATTTTCT
cow   AAAAAAAAA     GTCCTTTTGTCTTT GAGAAGAAGGCCTCCCTTTAAAGACACACTTTTC
rat   T                                           TGTGAGATAGAGAATCCT
mouse T                                   TTATGTATTTTAAGATGGAGAATCCT
                                                    * * *   * * *

cow
ctagccacccgccactgcacctgaccagttttctgttggctggtgcaatccagtggtgagctaaaaataaccccagcttaggaaacagggttgttcttcatgtggatgactctgtgccgaaagcatgggaacaggctagaagaaaaagaaaaaaaaaaaaaaaaaataaagacacacttttc
mouse
ggggctggtgagatggctcagtgggtaagagcacccgactgctcttccgaaggtccagagttcaaatcccagcaaccacatggtggctcacaaccatctgtaatgagatctgatgccctcttctggtgtgtctgaagatagctacagtgtactaacatgtaataaataaattaaaaaaaaaaattaaaaaaaaaaaagaaagctacagtataaaaaaaaaaaaaaaaaaaaaaagatggagaatcct


human CGAGAGAACCCTTGTTCTACATAAATAGATGACCACTCTCAGAAGCCAAGGAAGCAGTTGTTTCAGGTTCTTTCAATTTT
cow                TCACAGAGAACCTTTATAACCACTCTTATAAAACAAGAAAGCACTTTTCCCAGGTTCTCTTGGTTTT
rat                                TTTCAT TGATTGG GTTGAGTAAGCAGTCCTTTCAGAGTCCCTTGA
mouse                          TCCTTATCAACTGACTGG GTTGGGGAAGCAGTCCTTTCCG  TCCCTGGG
                                   *  *   *            * ***** *  *  * *  **  *
human CTTTCCAATCTCAACCGTCAGTGCACGGAGCTGTCCAAGGTTTTTATTTCTTGCCCTT CCCGTTCCTGACCCCTTTAA
cow   CCTGCCAATCCCAGTGGTCAGTGTGTGGAGCTGTCCAAGGTTTT ATTTCTTGCCCTT CCTGTTC TGGCCCCTTTAA
rat   CTTCCCT GCACGTTGC CAGTGCATGGAGCTGTCCAAGGTTTTTGGT GTTGCCCTT GCTGTTCCTGACCCCTTTAA
mouse CTTCCCTTGCACGTGATTCTGTGCATGGAGCTGTCCAAGGTTTTTGGT CTTGCTCTTTACTGTTCCTGACCCCTTTAA
      * * **   * *      * ***   ******************   *  **** ***  * **** ** *********
```

The 4-way genomic alignment shows that the cow-specific U65 snoRT (small blue letters, TSD highlighted in gray) and a mouse B2 element (underlined, TSD highlighted in yellow) have inserted at nearby sites, whereas the orthologous site in the human or rat genomes is empty.

human: chr5:43,226,394-43,226,842

cow: chr9:4,175,756-4,176,360

rat: chr2:51,803,315-51,803,689

mouse: chr13_random:65,147-65,784

**15- Primate-specific U3 retropson (extragenic); parental gene: U3**

**dog	ATTGACTAATTTTACAAGTTTGGCTAAATTAATTTAC	TTCTCTTAACATGAGCTTTCACCTTTTGTAAAAGATGAAAAAGA**

**human ACTGACTAATTTTACAAGCTTGGGTAAAATTAAATTCGTAAATTAACCTCTCTTCAGTTTTCACATTTTGTAAAAGAAAAAAAAAA**

*** **************** **** **** * * * * ** * * * * ** ****** ************ ***** ***

aggaagactatactttcagggatcatttgtatagttcgttactagagaattttctctgaatgtgtagaacaccagaaaccacaaggaggaggcgcagcgttctctcctgagcgtgaagccgggtcctggtgttgcttcactgcaactgccatttgccattgatgattgttcttctcttcctttgggagagtaagaggcaaaggatgcagtctgaatggttcctattaaaaaaacaaacaaagaaacaaaaa

**human CCAGGAGGAAAAT AGCTTGGTTAATTTTGCTGTGTGGTACTGTGAAGAAATGGAACTTATAATAAAGAGTTTATAAATTATA**

**dog	CAGAGAGAAATATTAGTGTGAATGTTTTTGCTATGTGTCTTTGTGAATAAAATGGAAACACACAGCAAAGCTCTTCAAATATA**

***	*** ** ** ** ** * ******* ****	****** *** * *	* *	* ** * * ** ******

This snoRT contains a full-length U3 copy (blue).

human : chr15:56,839,843-56,840,261

dog : chr30:26,737,063-26,737,226

**16- Primate-specific U3 snoRT (extragenic); parental gene: U3**

**dog	CAGGTAAAACACTAGAAATTCCCCCTTAGGTGAAACCAGCTAGCCCAAAAGAGAAGACCTACACACACCG ACATTTGAGGATACC**

**human CAGGTAGAACATTAGAAATTTTTCTATGGGGGAAACCTACTAGCCCAAAAGAGAAGACCTACACATACTTGTACACATGCAG**

******** **** ********	* * ** ****** ************************** **	*** ** ***

aagactgacatggggagaatccagattaagagtcacctcgtcaagactatattttcagggatcatttctacagtgcactactagagaagtttctgtgaacttgtagagcaccggaaaccatgagcaggaagtgcagcgttctctcctgagcatgaagccggctcttggtgtggcttcgctgcaactgccattggccattgatgatcgttcttctcttctctgggagagtaagagagagaggacacagtctgagtggttcccatctgaaaaaaaaaaaaaaagagagagagtcgcctcatcaccttagcgtccctatagtaaatagagagtaaggcacacaagtctgattccaatcagagtgaggggtagtttttcggtttttttaa

**human GAGAGAGAG TCGCCTCATCACCTTAGCGTCCCTATAGTAAATAGAGAGTAAGGCACACAAGTCTGATTCCAATCAGAG**

**dog	CAGTGAAAAATCATTTCCTCACATTATTATGTTGATGGCAACAAGAGAGCAAGGCATACAAATCTGATCCTGATCAGAG**

**** ** * **	** **** ***	*	** * ** ****** ****** **** ****** * *********

This snoRT contains a full-length U3 copy (blue) and ~100 bp of unknown origin downstream of the polyA tail. It has no TSD.

human : chr1:90,895,771-90,896,238

dog : chr6:61,090,628-61,090,791

**17- Primate-specific ACA5b snoRT in the MYLK, anti-sense orientation; parental gene: TBRG4**

```
cow   TTTACATATGACCACC      CTAATCACTTCTA     CCCTAAATCTGAATATCAGACATGACTTCAAGGATGTCTTCTCT
human TATACATAAGGCCACCTCTACCCTAATCACTCCCATGTCTCCCTAAGTCTAGACACAAGACATGACTTCAAAGATATCTTCCCC
      * ****** * *****      ********* * *     ****** ***  * *  ************** *** ***** *

atgcagctgtgtcaaatttggtgcctgccctgcctgtgtaggcactgacccagaaggctgccacagaaacactgactcatgggccctgttcctgtgtctcgggctcagggataaatttggttacagataccaagcctctagaaaaacaaaagacaaaggtatcttcccc

human              GGCCACATTCACCCTATTCCGGCCAAGCTCAGGCTTCATGTCCCCTCTGTCCAGGAACCTGTT
cow                GGCCACGTTCACTCTGTTCTGGCCAAGCACATGCTTCACATGCCCTTGGTTTAGGAAATTGTT
                   ****** ***** ** *** ******** ** ******  * ****  **  *****  ****

This snoRNA is retroposed with 9 downstream nt of parental origin (red letters).
```

human: chr3:125,015,523-125,015,844

cow: scaffold970:32,792-32,938

```
18- Rat-specific ACA5 snoRT in the Zfm1 gene , sense orientation; parental gene: Tbrg4

mouse CTCATGCATTGCCTCCTTAT          AAATTAACATTGACTCT GTCAGGTCTACAGAACTTGACTCTTAAAAGGAGGACTGTG
rat   CTCATACTTTCCCTCCAATTTGGAATATCCAAATTAAAATTAACTCTTGCCAAGACTACAGAACTTGACTCTTAAAAGGAGGACTGTG
      ***** * ** *****   *          ******* *** ***** * ** * *********************************

tgtagctgtgtcaaattcagtgcctgtcctaatgatggcatgcactggcccagaaggctgccatagaaactgtgactcctgagacctgttcccatgccaggttcaggatgaatttagtcacacacatcaaggaaaaaaaaataaaagaggactgtgagggctaaaagaa

rat   AGGGAGAACAAGAAA  GCGACAGG GTTGAGGAAAGGACAGTGTTT    CGTGCAGAATTTAGAT
mouse AGGGAGAACAAGAGACAGGGATGGGAGTTGACGAAAGGATTGTGTTTATTTCATGCAGAATCTAGAT
      ************* *  * **  ** ***** *******  ******    * ******** *****

rat: chr4:118,182,171-118,182,487
mouse: chr6:83,949,113-83,949,256
```

**19- Rat-specific ACA15 snoRT in the Fgfr1 gene, anti-sense orientation; parental gene: Cct6a**

```
mouse CACATGCACGCACA ACGTGCAGATACTGGTCTGAGAGAGAATATCTGGCCCCTTCTTTTTGTCACTATCAAAAACTTCAGTCAGAA
rat   CACACACACACACACACACGCATGCACACA CAACCTGCAGATATCTGGTCCCTTCTCTTAGTCACTATTGAAAACTTCAGTCAA
      ****  *** **** **  ***   **    *     *   ******** ******* ** ********  *************

cagcatggctgaatactgtgttctttagtcagtaatttacatagccagacaccatgcaagagtaaagtcttcctttaagatggctgctatgctgtattttatggtatatcattaaaagtgagtacaagtagaaaaaaaaaaaaagaaaagaaaaaagttcagtcaa

rat   ACATCATTCCTAATATCTCCCAAGTCTACAGAGAGGCACAAACACTTAGACACACACACACACACACA
mouse ACATCATTCCTAACATCTCACAAGTCCGCAGACAGGCACAAGTACTTATACACAGAAACGCACACACA
      ************* ***** ******  **** ********  ***** ***** * ** ********
```

rat: chr16:70,878,639-70,878,956

mouse: chr8:27,029,318-27,029,471

**20- Rat-specific ACA36 snoRT in EST CD373301 (sense); parental gene Dkc1 (?)**

```
mouse GTCTGTGTGCTTGTTACAGAGAATTTTCT CCCCCTCAGCAATGTGGCCATGAATTTTTAATTTTGGTAAAGCTTCATTTATTTTCC
rat   GTCT      TTGATATAGATAATTTTCTTCCCCCTCAGCAATGTGACCATGAATTTTTAACTTTGATAAAGTCTCATTTATTCTCC
      ****      *** ** *** ******** **************** ************** **** *****  ********* ***

aaagtgttgagttcagttcagggcaatttccctgttctgttaattaaactttgggacattgaaattggctgagggaaataactgaatagagaatattactctattcatatacttctagcctacacaaataaaaggaaaaaaagaaaaagaaaaaaagtctcatttattttcc

rat   TTTTTCTTAATTTTTGGGTGTCTATGTATTTGCCCATTCTGCTGTGGTTTTC TTGTGGT
mouse TTTTTAAAAAATTTTGGGTGTCTATGTATTTGCCCATTCTGTTGTATTTTTTGTTGTTTT
      *****   ** ****************************** ***  ****  ****  *
```

The putative Dkc1 (dyskerin) parental gene could not be localized in the rat genome, and is inferred from other vertebrates. The snoRT host gene is orthologous to the mouse 1110007A13Rik gene **(**hypothetical protein LOC210711).

mouse: chr7:128,524,393-128,524,538

rat: chr1:187,989,070-187,989,381

**21- Rabbit-specific U65 snoRT; parental gene: RPL12**

```
human  TTGTCTTGTGATCCACTACCTATACCCATCTTAAGGTACCATTGTGACACCATGTCAACACA GGGCTTCTCT
rabbit TTGTTTGGGGATACATTTCTCAGTCCCATATTAGGGCACCATTGTGGCACCATCTCTACACAAGGGCCTCCCT
       **** * * *** ** * *  *  ***** *** ** ********* ****** ** ***** **** ** **

tagccacccaccactgcgcctgtccatgctgttggctggtgcaatccagtggtgagccgagaggaagccccggcttaggaaacagggttgttctccacgtggatgactctgtgccgaaagctcgggaacagcctaggaaaaaagaaaaaaaaaaaaggggggggggcctccct

rabbit GAGGGGAAGGCAGTTTGAAGAGGATGTCACTGGGGAACTGAAGGATAAGTATTTTGTTCTGGGCCATCCTGCTGAATGTCTCAGGA
human  GAGGAGCAGGTATTTCGAACAGGCTGCAGCTGGGGGATTGAGGAATACACTTTTTGTTCTGGGATTTCTTGCTAAATGTCTTAGGA
       **** * *** * ** *** *** **   ****** * *** * ***    ************   ** **** ******* ****

rabbit: scaffold_179357:174,369-174,700
human: chr3:157,977,893-157,978,050 in mRNA AK056401 (sense)
```

**22- Primate-specific U68 snoRT (extragenic); parental gene: RPL18A**

```
dog   AACCAAACTAATTATATGTAATCTTTTCTGGGCATAAAA CTGCCATCA
human AACCAAACTCATCATAAATAATCTTTTCTGGTCATAAAAACTGCCATCA
      ********* ** ***  ************* ******* *********

ctcatacctaaacccaagaatcactttcttatagtgatgatttaaacagatgcaaacagcgagcacatcttgtcacctttgcgggactgtggctgtgcccctcgcagtaaatttggaggttctacatccctggtgaaaaacaaaacaaaacaaaaaaaaagcctgccatca

human TTGACACCAAAACACTCATAATGACAGTACCCACTGTAA
dog   ATGACACCAAAAAGTGCATACTGACAGTTCCCACAATAG
       ***********    **** ******* *****  **
```

human: chr5:158,589,730-158,589,989

dog: chr4:54,464,106-54,464,192

**23- Dog-specific E2 snoRT (extragenic); parental gene: RPSA**

```
cow TAATGATGAGTATATTAAATTAATGTAAATTATGTAAACTTAAATAGTGAGCTGATTCCAGTGTAAATAAAGAATGAGAAAATGAAGGTG
dog TAATGATGAACATATTAAATTAATGCAAATAACATAAACTTAAATAGTGAGCTGATTTCAGTGCAAATAAAGGATAAGAAAATAAATATG
    *********  ************** **** *  *********************** ***** ******** ** ******* **  **

ctgcacattgttagagcttggagttgaggccactggctggccgatgaactcacaagtgtaggtagtgtgctacatgaggggcaagtcttttcgctaacaccacaagggtctctggcccaatgagtggagtttgatagtaattcttgctacaagtagaattaaaaaaaaaaagaaaaagaaaataaatatg

dog CTGGGTAAA TGGAATCATGTAAAATGAT   TTGACATGAATGGATGAGAAATAAAAGTGTGAATAT
cow CTGTGTAAAATGGAACTGTGTAAAATGATGATTTGATATGAAGAAATGAAATATAAAATTGTGAATAT
    *** ***** *****   ***********   **** *****   **** * ****** *********
```

dog: chr30:37,744,141-37,744,484

cow: chr10:12,993,222-12,993,379

**24- Dog-specific E2 snoRT (extragenic); parental gene: RPSA**

```
chimp AGCATTCTAAGAATAAGGAAACATACATGAAAACCCTAGATGGCAAAAGCTTTCTTTTGGAGGAACGATTTTAAAATGCTAATTCCA
dog   AGTAATCTAAGCCAAAGGAAGCAAGCATGAAGGTCCTACATAGCAAA  GTTTATTTAGAAAAAGCAAAAA AATATACCAGTTCCA
      ** * ******   ****** **  ******   **** ** *****   *** *** * *  * * *    ** ** * * *****

dog
ctgcacatgggcagagcttggagttgaggccactggctggctgatgactcacaagtgtaggttgtgtgctacatgagaggcaagttttttgctaacaccacaagggtctctggtccaatgagtagagttaatggcaagtcttgctacagttaaagaaagaagaaaaaaagaaagaaagaaaaagaaagaaagaaagaaagaaagaaagaagaaagaaagaaagaaagaaagaaagaaagaaagaaagaaagaaagaaagaaagaaaaaagaaaggaaggaaggaaggaaggaaggaaggaaggaaggaaagaaggaaggaaggaaggaaggaaggaaggaaggaaggaaggaaggaaggaaggaagaagggaaagaaaggaagaaagaaagagaaagaaaggaagaaagaaagaaaaagaccaaaaagcctattcccattctaggccttaagaatttagg

chimp
gggtgtagtgggaaagaaaaagtaacaaaatgttgtagagacagacagaaatcaaatcactcagagatttttaaaaaatattctaataagattgaagcagttgaattgaggctaattttgaactctttttgttcaatagaatataataaaagtaatactaaggctttttgcaggttgatcgcttaaaaaccctatc

dog   ACTACCATTCTCCCTCATGAATCCATTTGTCATGTAAGAAGTTGAACTGCCCTAAGAGCATGACACCGAGAGGAAGCTCCAGCTAGC
chimp ACTACAATTTTCACTCATGAATCTAGTTGTCGTATAAGAAATTCTACTACCCAGAAGGTACCATGCTGTGGGAAAGCTCAAACTAAC
      ***** *** ** ********** * ***** * ****** **  *** ***  *  * *  *  * * * * ****** * *** *
```

This snoRT is inserted between L2 and MaLR elements. It has no TSD, and, compared to chimp genome, its insertion might have been accompanied by a ~ 0.2-kb deletion. Note the 270-bp tail mostly composed of GAAA repeats (small red letters).

dog: chr13:54,777,031-54,777,661

chimp: chr3:85,724,733-85,725,102

```
25- primate-specific E2 snoRT in the PSD3 gene (anti-sense); parental gene: RPSA

cow   TTTTATGAG TTTTTCATGAGTAAAGCATTTCTTTG ATATTAAATAAACCATGCTTTTCAAAGTACAAAAGAACTCTCTTCT
human TCCTTACAGCTTATACATGAGTGAAGCATTTATTTTTATATTAAATAAATCATGCTCTTTAAAATAAAAATGAA TATATTCT
      *  *   ** ** * ******* ******** ***  ************ ****** ** *** ** *** *** * * ****
tcttgttagagctcggagttgaggccagtgactggccaatgaactcacaagtgtagacagggtgttacgcaagggacaagtttttcactaacaccacaagagtctctggcccaatgagaggagtttaatagaaattcttgctacaactaaaagaaagaaaagaacaaataaaagaatatactct

human CCTACTGTAAAATCTACTTAAGAA AAAGTAGGTTATCAGGTC TTTATTGCAGAAA
cow    ATCTTATAAAATGCATTTATGAAGAAAGTAGGTCATTATGTCCTTAATTGCATAAA
        *  * ******  * *** *** ********* ** * *** ** ****** ***
```

This E2 retrocopy is deleted of 8 bp in 5’.

human : chr8:18,881,333-18,881,653

cow : chr27:25,674,706-25,674,842

**26- Dog-specific E2 snoRT in the MIPOL1 gene, sense orientation; parental gene RPSA**

```
human TAATTTGATACACTGTAAAATTAACACAGTTAACAAACAATGTTCATTGATGTGAACAGTTTTAGTATAgaaatataatcaca
dog   TAATTTGACA   TGTACCAA AATATAGT        AATGTTTCTTGATTTATCAAAATTAAGTACA
      ******** *   ****  *  ** * ***        ******  ***** *    *  ** **** *

tgcacattattagagcttggagttgaggcctctgactggccaatgagcttgcaagtgtaggtagtgtgctactagaggggcaagtctttcagtaacaccacaggggtctctgatccaatgagtagagtttgattgtaattcttgctacaagtataaaaaataaaaataaagaaagaaaagaaaaaa

dog   TTAAAGCATTAACAACCTTTATTTTGTTCTTTAATTAATGTCTTTTTTATTTCTCCCCCCCCATTC
human TTCAAGCACAAGATACCATTATTTCTTTCTTTAATTAATGT   TCTCCCCACTCCAGAAA ATTC
      ** *****  *   *** ******  ***************   * *     ****      ****
```

This dog-specific E2 snoRT has no TSD. Compared to human genome, its insertion was accompanied by a 14bp deletion.

dog: chr8:18,789,719-18,790,027

human: chr14:36,795,014-36,795,158

**27- Mouse-specific U23 snoRT in the Dpep2 gene, sense orientation; parental gene: Ncl**

```
rat   TGCTCGGAATCTCCCTGATGATATCCTGCAGCTCCTGGTGAGAAATGTTCTTTTAGCCCTGGAACCTAAATCAAGGCTTGAAACCCCT
mouse TGCTCGGAATCTTCCTGATGACCTCCTGCAGCTTCTGGTGAGAGATTTCCT   AGCCCTGGAACCTAACTTAAGACCTGAACCCCC
      ************* ********  ********** ********* ** * **   *************** * *** * **** ***

atcttctcattgaacccctttctgtcaaccagtgatagattatggattcgcacgagaagaagagaattcacagaactggcacttatcttctgtttttgcagaagtatatttggctgttgtgtgaggcattctgaagaaaaaaaaaaaaaagaaaaagaaagacttgaatcccc

mouse GTCCCTTCATCCCCTTAACCCTCAGAGCGAGCATTCTCTGCCCCACAAAACCATAAACCATGGCCTCTCAATTCTTGAACCTGGGGTA
rat   GTCCCTTCAATCCCTTAGCCCTCAGACTGAGCATTCTCTGCCCCACGAAACCATAAACCATGGCCTCTAAATTCTTGAACCTGGGGTA
      *********  ****** ********  ****************** ********************* *******************
```

The blue sequences in the upper alignment correspond to the 3’ end of exon 7 of the Dpep2 gene. Note that the ACA box of the snoRNA copy is mutated to GCA.

mouse: chr8:108,877,500-108,877,844

rat: chr19:35,835,517-35,835,692

**28- Primate-specific E3 snoRT in the C1orf102 (NOR1) gene, sense orientation; parental gene: EIF4A2**

```
dog   GTGAGTGGGTGACCCCACAAGCCACGTGTCGGTCCCCTCCTCATCTGCTA  ACTTTGAACTCCCCAGGCCCCCCTTC
human GTGAGCCACCGTACCCA   GCCTCCCTTCC TCCTTTCCTCATCTTATATAACGTTGAACTTCAGAGACCCTCTTTTTC
       *****     *  ****   *** *   **  ***  *********  **  ** ******* *  ** *** * **

aagcaggattcagactacaatatagctgttaagtgctgtattgtcattccccctgctcaaattaaagttgtttcttaactatacccatctgctattctgtagcagccagggatgcttggtcacatacatgttgattaagaaaaaaaatatatagaccctcttcttc

human TCTCTGATCAGCCTGTAACTACAATCCCTTTTCTGACAAACAGGTGCGCAACTGAAACATATCAGTCATGGGACAAA
dog   TCTCTAATCAGCCTGTACCCAGAGCCCCTGCTCTGACACACGG   CACCACGG               GGGACAAA
      ***** *********** * * *  ****  ******* ** *   * * ** *               ********
```

human : chr1:36,656,524-36,656,842

dog : chr15:8,884,541-8,884,675

**29- Rat-specific ACA62 snoRT in the Fsd2 gene, anti-sense orientation; parental gene: U104/ACA62-HG**

**mouse CTGTGTCTGGCTGGCTCCCTTCCGAGCTCCTCAGCCAGGTTCCCCAAGCACCCAACACTGTGCTCAATGTGACTACTGG**

**rat	CTGTGTCTGGCTGTCTCCCTTCCAAGCTCCCCAGCTGGGTTTCCCAAGTACCTAACACTGTGCTCAACATGTCCACTGG**

*************** ********* ****** **** ***** ****** *** ************** ** * *******

agcatttggggagtatgcacctgtctctgtctctggccatgtgagagccatgacctctctgagggcaggaccaagtacacaaggacagtcctaaccatagaaacctttctgtgacgcgctgtctttgagcccccggtaaaccttcttgtggtgctgggggttaagcttcagtgctatagcattttgctggcgcataactggggccgcctgcctatcgcggctaaggctaaggccgcccttttaaccactagctacaggctaaaaccaaaaaaaaaaaaaaagaaacctttcggtatgagcatctatgaacagacaactttgtttaa

**rat	AACAACTGCTGCAGAGAATAATCCAATTCATTTCAGTTGGACCGTATGGAAAT**

**mouse AGCAACTGTCACAGAGAATGATCCAGATCATTTCAGTTGGACTGTACGGGAAT**

*** ******	******** ***** *************** *** ** *****

The snoRNA sequence was retrocopied with 3 + 8 bp of parental origin (red letters, compare with snoRT 30 in this Figure). The snoRT is embedded in a 98 + 32-bp sequence of unknown origin.

rat: chr1:137,758,917-137,759,374

mouse : chr7:81,415,836-81,415,967

**30- Rat-specific ACA62 snoRT (extragenic); parental gene U104/ACA62-HG**

```
mouse GTATGTTTATTTGGACTCTCTTAAGTGAGTCTTATGATAAAACCGTACCAT  GTTTTGTTCATCTTAT
rat   GTATGTTTATTTGGACTCTCTTATGTGAGTCTTATGATAAAACTGTACCCTAAGTTTTGTTCATCTTAC
      *********************** ******************* ***** *  ***************

gtgctgcctttgagcccccggtgcaccttctcgtggtgtcgtgggttaagctgtgacgctatagcactttgctgggaccgcctgcctatcgagggcggcctttttaaccgttagctacaggctaaaaattaaaattaaaattaaaaaaaaaagttttgttcatcttac

rat   GTATTCTAGTAAGAAACTGCTTGCTTCGATCTACATATCAGCCGCGAAGGACAGAATGAGAAAAGCAGTCGCCAACCACAGCCACAAA
mouse GTATTC AGTGAGAAACTTCTTGCTTAGACCTACAGATCAGCTATGAAGGACAGAACGAGAAAAGCAGTTGCCAACTGCAGCCACAAA
      ****** *** ******* ******* ** ***** ******   *********** ************ ******  **********

rat: chr17:7,201,773-7,202,097
mouse: chr13:63,461,775-63,461,928
```

**31- U109 copy in the UCHL5 gene, sense orientation; parental gene: TMEM167 (human) and 0610041E09Rik (mouse)**

```
cow   GTGAGAGACTAGATAACTCTTAAGATGGTCAGAATCTGA GAAGATTAGCTCAATATGAAGTAGTGATTTGGAGAGG
dog   GAGAGAAGCTAGAGGTGTCTTAAAATGATTGGAGTCAGATGATGAAC  CTAAAGTAATGGGTTAAAGAGGAGGAAG
human GAGAGAAGCTAGACATCTCTTAAAATGGTTGGAGTCCGA GATGATTGGCTAAACTAGTGGGTTAGGAAGGAGGAGT
mouse GAATGAGA TGACTCTTTTTGAAAATGGTAAGTTTGTG  GGTGATTGGCTTGACATTAAGCCCTGGGGTACAGAGA
      *   **   *       * * ** *** *  *  *  *  *  **    **  *      *            **
cow   AGGTAG  TCAATAATTT  TGGCTTTAAGTCCACAG
dog   TAGTTG  TAATGATTTTGCTTTATTTAAACCAGCAGGACA
human TAGATT  CAATAAGTTTGCTATATTTAAATCAACAG
mouse AAGTAGAGTCAGCAGTTAGGTAGTATTTCAATCTCAGG
        *       *  * **   *    **       ***

human TTGCATGTGGTAATGTCTGCTCTTCATTCCTTTGGGAGCAGAAATACATCCATAACACGCTAAATTAAAAAAGGAGTTCT
mouse TTGCATGTGGAAATACCTTCTCCTCATTCTATTTGGAGCAGGAATGCATCCAGAACATGCTACACTAAAAGAGGAGTTCT
      ********** ***  ** *** ******  ** ******* *** ****** **** **** * ***** *********
human CAGGGCTGCCAACCTTACAGTAGAGGTTGAGTGGTAGTATATTACTCCTACATAA GAGAAAA AAAATCAAAAG
mouse CAGGGCTGCCAACCTTACAGTAGAGGTTGAGTAGTAGTATATTTCTCCTACAGAAAGAGAAAAGAAAATTAGCAG
      ******************************** ********** ******** ** ******* ***** *  **

cow   GACAGTTAACTGGTGGGATAAAGGGGGAAAGGTGATAATGACTTTAGTC*TCTAGCTTCTAGCTTAGGAGACTGGTGAAG
dog   GCCCACTGACCGGATG     TAGGGGAGAAATG    TGAAAACGCTC CAGGGTTTCTAGCTCGAGTGACTGGTGAAGC
human GACTGCTTACTAAAAG    TTGGGGAAAAAAAGAGGCAAAAATAACTC CCTGGTTTCTAGCCTGGGTGGCTGGTGAAAC
mouse GA  GTTGACTGACTG     TAGGAAAGAAGAG           TCAA ACGTGCTTCTGGCTTGGGTGGATAAATAAAG
      *     * **     *       **  * *   *                    * **** **    * *  *    **
cow        TAAAAGAGAAGAGTAGGCGTAATGGACC
dog             TTGATGAACAAGCTTGATAGACA
human ACTCTTACAAGTGAAGAGCAGGCTTGATGGACA
mouse     TATCATTTGAAGAATAGACTTGATAGGAA
                  ** **  *  * * ** *
```

This copy of the U109 snoRNA is present in the first intron of the UCHL5 gene (sense orientation) in primate, rodent and rabbit genomes, but the orthologous site in the cow and dog genomes is empty, reflecting an ancient insertion event. The U109 copy is in blue in the human/mouse alignment. No apparent TSD. A star in the lower cow sequence indicates the position of a 177-bp insertion containing Bov-tA3 (SINE) and Bov-B (LINE) elements.

**U109 copy present**

human: chr1:191,292,907-191,293,279

chimp: chr1:172,334,755-172,335,127 (not shown)

rhesus: chr1:177,540,254-177,540,627 (not shown)

mouse: chr1:145,541,034-145,541,391

rat: chr13:57,607,393-57,607,701 (not shown)

rabbit: scaffold_115852:12,996-13,373 (not shown)

**U109 copy absent**

cow: chr16:4,770,253-4,770,645

dog: chr38:9,005,956-9,006,163

**32- Armadillo–specific U109 snoRT**

```
cow       GCGAGACTGAAAAGCTAGATTGTGGAGCTCCAAGCTGCCAGGTGTCCTGCCTTGTGGCAAAAGGTAACATTTGGAGATAAATG
armadillo GTGAAGCTTTAAGGACAGACAG  GCGTGGCCTGCTGCCACATGCCCTTCCCTGTGGGAGAAGGCGACACGTAGAGAGAAATG
          * **  **  ** *  ***  *  * *   *  *******  ** *** ** ***** * ****  ***  * **** *****
```

#### **aaaatgacagacattgcatgtggaaatgtctgctcctcattcttttaggagcaggaatatatccataacctgctacattaaaataggagttctcagggctgccaaccttacggaagaggttgagtagtggtatatttctccaacgttaaaaaa**

```
armadillo AAAAAAACAGGCAGAGGAAGGGGGTCCTGATAGGGTTCAAGTCAATTCTAAG TCCAAGGTGACTTGTTACCTCACCTTC
cow       AAAAAGACAGGCCAAGGGAGCAG TCCTGACCAGGTTCTGGCCACTTTTAAGATCCAGGGTGG TCGTTAACTTGACATC
          **** ******  *** **  * ******   *****  * ** ** **** **** ****  * **** **   * **
```

A potential TSD is boxed.

armadillo: scaffold_59040:12,824-13,155 (May 2005 assembly)

cow: chr11:17,196,432-17,196,592

**33- mouse-specific U99 snoRT in the St3gal2 gene, sense orientation; parental gene: U99HG.**

**rat	CTGAGCCGCGCACTTGATCGTGTGGAAATTCTTTAAATACATCTCCATTTCTGAAG**

**mouse	CCGAGCTGTGCACTTGATCTTGTGGACATTCTTTAAATTCATCTTCATTTCTGAAA**

*** **** * ********** ****** *********** ***** ************

tgctttcatctgcccagtccttggcatatagtaggtgctcaggaaagttgaagcagagattttgttgttggtttgtttgtttgttttttctccagatagggtttctccgtgtagccctggctgtcctagaacttgttctgtagaccaggctggcctcgaactgggagaaccatgtgcttctgcctgctggattaaaggtgtgagccaccactttccagctttgtttttatttttattttttaaaaagtaattaa**gcttttttcaagggtggttttcacgtgagtcttgattttgtctttatatgcgagggaactcctgggaactgggcctgactttgtagatcctccttttcttggcgggtaccgggcgtgcggtcccgcttcctgtaatgtacggaggtagaagaaagggctctggccctctcggcgttatgtcttcggtgcttgcggcctcctgttctcgggttctatcctcagacgccggaacacttaa**aaaaaaaaaaaagtaattaattggagatggtgcatgtagggcggtgtgtgtgtgtgtgtgtgtgtgtgtgtgtgtgtgtgtgtgttgacccatgtgtggaggtcagaggagaactttcgggagttagtttttcccttctactacatgggttctggaaatcatagttaggtggtcaggtttggttgcaagcccctttgtccaccaagccttcttgcctgtctttgctttcatttctgaaa

**mouse TGGTATCTTACCATGTAGCCCTGGTTGGCCG	TATCGTGTAGTC	CGATGCAGCTTGGAACTTTCAGTG AT**

**rat	TGGTATCTTACTGGGTAGCCCTGGTTGTCCCGGAACCTACCGTGTAGTCTTACGATGCAGCTTGGAATTTTCATTTTCAA**

*************	************* **	** *********	*************** ***** *	***

This mouse-specific insertion (overall TSD highlighted in aqua blue) contains an L2b element (nts 415-460), a B1 element (nts 147-8), the U99 snoRT (blue, TSD boxed), a GT microsatellite and a B4 element (nts 133-8). The sequence in bold letters corresponds to the 5’ end of the full-length 5730408K05 Riken cDNA clone (AK017527, 3271 bp). The snoRNA retroposon was described by Vitali et al. (2003).

**34- Cow-specific U99 snoRT and analysis of the orthologous segment in human and rabbit genomes**

**cow	AAATTATTATTTATTTATGTTGCCCTCTATATAGACACATT AAGAAACAATTTGTCTCTCATTCTCTTAAAGAGACAAAAATATA**

**human AAATTACTGTTTAT GTTATTATCTGTGCAGGAACAATCAAAAAATAATTTTTGTCTCATTCTGCTGTAGAGACAAAAAATTT**

**rabbit AAATTACTCTTTAT ATTACCTCAGATGCAGGCACCATAGAAGAAGGATTTGTCTCTCATTCTCATGTAGAGACAAAATACTG**

******** * *****	**	* ** ** * * ** **** * ********* * **********	***

**cow**

tgtttgtttgtttgtttttaaaa**actgagtgagtgtggccaagtaaacgtgggggttgggctggggatctcagggagggagacggtgcctgtggaccctccttttcttggcgggggccgggcgcgcagtcctgatctgcataaggtacagatgagggatgggctctggccccctcggagtcatgtcttcagtaacagaggccacaagtctactggttctatcctcaagcgctgggacacagag**ggaaaaaaagatgggggtgggggcggagaaaaaaaaagaaaaagagaaaggaaaaaagaaaaaggaagaaaaaaagaaaaaaaaatcacagtagctg

**human**

cgttctttctttttttttgaaatggagttttgcttgttgcccagactggagtgcagtgtcgagatctcagctcactgcaacctccgccttgcgggttcaagagattctcctgcctcagcctgtcgagtagctgggatataggtgcccacaaccaagcccagataattttctggtaatttttagtacagacggggtttcaccatgttgacgacgctggtctcaaacccctgacctcaaatgaactgcccacctctggttcccaaagtgctgggattataggcatgagccaccacgcctggctgacaaaaatatttttaaaaattctcctaatagctgttttgttttgtttgtttgttttttattttagGATCTTGCTCTGTCACCCAGGCTGGAGTACAGTAGCACCATCACTGCTCACTGTAGCCTCGACCTGCTGGGCTCAAGTGATCCTCCCATCTCAGCCTCCTGAGTAGGGGACTACACACAGGCACCACCATGATCCGgtaatttttaaattttttgtagagactgggtcttttttttttttttttttttttgagatgaagtctctctctgtcgtccaggctggagtgcagcggcgcgatctcggctcacagtaacctctgcctcccagattcattcaagtgattctacttccttagcctcccaagtagcagggactataggcgcccgccaccacgcccggctaatttttgtttcttttttggttgttgttgttgttcgagacagagttttgctctgtctcccaagctggagtgcaatggcacgatatcagctcactgcaacctctgcctcccgggttcaagtgattctcgtgcctcagccttccaagtagctgggattataggtgcgcgcgaccacgtccagctaatttttgtatttttagcagagacagagtttcaccacgttggccaggctggtttcgaactcctgagctcacacgatctgcctgctttgccctcccaaagtgctgggattatagatgtgagccactgctccctgccctttttttttttttttttttttcagagtctcactctgttgcccaggctggagtgcactggtgcaatctcggctcactgcaacctatgcctcccgggttcaagtgattctcctgcctcagcctccggagtagatgggattacaggtgcccgccaccacacccagctaatttttgtattttcagtagagacagggtttcaccatgttggccaggctggtctcaaagtcctgacctcaggtgatccaccacctcagcctcccaaagtgctgggattacagatgtgagcccagcccctgactgctgt

**rabbit**

ttaaaattctccaaatatctgacatgtatcagggtcttcaaaaacctcctggaaaaatgcctattaggaaaaaactatggatggatttcaaaaaaagttttgcaccaaaataaagtcatcatttgacttcattttccatgcactttgaaagg

#### cow TTGAGATGTGTGCTAGCTTGATGAACTTGTGCTGAATATGGAAATACTCTTCCAGGCCAAGGAAATCTCAAAGAAAATGATCACCTT

#### human TTGAGATATATGTTAGTTTCCTGAATTTGTGCTGAATATGGAGATGCTCTTCTAGGCCAAAGAAGTATTAAAGAAAATGATTACCTT

#### rabbit TACCCTCTTAGGTTAGCAAAGTGGACTTGTGCTAAATATAGAAATGCCCTTCCAGGCCAAGGAAACATCAAGGAAAACGGTCACCTT

####	*	* * ***	** * ******* ***** ** ** * **** ******* ***	* ** ***** * * *****

The cow 339-bp insertion contains the U99 copy (red) and 73 bp of upstream sequence from the parental U99 copy (bold letters). It has no TSD. At the corresponding site of the human genome, there is a 1.3-kb insertion comprising 5 *Alu* elements (underlined), one of which provides several ESTs (like DA171963) with an exon (capital blue letters). The corresponding region of the rabbit genome contains a 152-bp sequence unrelated to the cow and human insertions.

#### cow: scaffold_294:4,989-5,500

#### human: chr7:77,127,539-77,129,035

#### rabbit: scaffold_194000:58,593-58,913

**35- Primate-specific ACA30 snoRT in the PGS1 gene (sense); parental gene: SRCAP**

```
dog    GTGTTTTCCCTACTTCATACACGAG     AGACTAGAGAGGATGAGTGACTTCATTGGGCAGAGCCAG
rhesus GTATTTTCCTCACTGTGTACATGTGGAAACAGATCAGAGAGGATGAGTCACTTTGTCAGGTAGA  CAG
       ** ******  ***   **** * *     ***  ************* ****  *  ** ***  ***

dog    GAACACAGAAGAAATGAACCGCAGAGCCAGGATGGCCGCCTGGGTCCTTTTGATTCTGAAA
rhesus AGCCAGAAAAATAAACAAGAGTCAGACCTGGAAGTCCA    GGTCCTTGGGTTGATAAAT
          ** * **  **  **  *     ** *** * **     *******  * *  * **

gtgggccctttcacaggccctcccctcggcagtgggaccaggattgggttgctggtgctgagagaaagcccttgattgcatccttgctctgggacggttccagtggcagctgtcacttagtgggacagagaaaacaaacaaacatgggcactgataaatagccctgcatgggccgggcgtagtggctcacgcctgtaatcccacactttgggaggctgaggcagagggatcacctgacgttagaagttcaagaccagcccggccaacatggtgaagccccatctctactaaaagtacaaaaattagctgggtgtggtggtgcacgcctgtaaccccagctactcaggaggctgaggcaggaggatcacttcaacctgggaggcggaggctgtagggagccaagatcatgccactatactccagcctgggcgacagaatgaggctgtctcaggacaaataagcaaacaaa

rhesus                 AGCCCTGCGTGGT   TAGCTGACTTTCTGTGTCTGCATCTCCT
dog    CCTGGGCACTGGGCTCAGCCCTG GTGGCGGGTAGTTGGCTTTGTGAGGGTCCCTCTTCT
                       ******* ****    *** ** **** ** *  * * *** **
rhesus CTTCTCAGCTAGATTGTCAGCTCCTTGAGAGGAGGAGCCTGTGGGTCACTCGCTGT
dog    AT   CAGCTGTCGCCTCAGCATC       GAGACTCC GTGGCTTCCTCGCTGT
        *   *****      *****  *       ***   ** **** *  ********
```

This primate-specific insertion is composed of a Type 1 ACA30 snoRT (snoRNA sequence in red, TSD boxed) immediately followed by a complete *AluSx* element (underlined, TSD highlighted in green). The snoRT was previously described by Bachellerie et al. (2002) as the HBI-26 retrogene.

rhesus: chr16:73,690,165-73,690,854

dog: chr9:5,827,409-5,827,637

human: chr17:73,907,553-73,908,168 (not shown).

**36- Rat-specific U17 snoRT in the Pdia3 gene, sense orientation; parental gene: not localized**

**mouse TAGGCTTAAATTTCTAATCCTCCTGCCTCTATTTTACATAA**

**rat	TAGGCTTAAATTTCTAATCCTCCTGCCTCCACTTTACAGGT**

******************************* * ********

cagggcattgtaggcatgtgccaccatatctgctttggatttcattctagaaattgaaataacattttattttgttaagatttcattgtgggggctggggatttagctcagtggtagagcgcttacctaggaagcgcaaggccctgggttcggtccccagctccgaaaaaaagaaccaaaaaaaaaaaaaagatttcattgtgttccaacgtggccatcccgggaggtcactttccccgggctctgtcctagtggcgtatgggagcatagggtactgcccgatgatgtacagtccctttccacaatgttggagatgaagctgggcttcgagtctgcgcctgcatattcctacgacctctcagagtcccgtggacagtgactgaggagacaaaccatgcaggaaacaaaaaaaaaaaaaaaagattttattgtgtaattcaaactagcctagaattagatgtgctaattttcttttttaatgatttgcatggtatgaaggattatgagagtggggtagggagacttcaaatgtgggcttaagctagccctgaataaatgtttcattcttagtagtaggagaaaaattgttctatacagcatacttaaaatcattaaaattcct

**rat	CATTTTAGAGGTCAACTTGTTAGGTAACAGAAAGCTTCTTT**

**mouse CATTTTAGAGGTAAACTTGTTAGGTAACAAAAAGCTTTTTT**

************** **************** ******* *****

This rat-specific insert contains a full-length ID sequence (green) and the U17 retrocopy (red). The two retroposons have inserted at the same site, creating an almost perfect triplication of the insertion site (boxed). The retroposons are embedded in a sequence of unknown origin, and the overall insert has no TSD.

rat chr3:108,227,754-108,228,457

mouse chr2:121165874-121165955

**37- Mouse-specific U68 snoRT in the Zbed3 gene, sense orientation; parental gene: Rpl18a**

```
rat   CAGATAGATCCTACACTATTGATTT CTGAGGA      GCCCCTACACCC           CATACTGATGAGTGAGTA
mouse CAGA  GGTCATACACTATTGATTTTCTGAGGAACGCACGCACACACACACACACACACACACACCCCGATGGGTATAAC
      ****  * ** ************** *******      ** *  **** *           **  * **** **
rat   TCCATT          CCCACCAGCCTTAGAAAGGGGCACCTTTTTCTCCATTGTTGGTGACGGTTGTTGGTGACGGTATTC
mouse TACCTTAATTTAGGTTCCCACCAGCCTTAGAAAAGGATCCCTGTTTCTCCATG  TGAAGACAAATGGCCACCAACAAACTA
      * * **          ***************** **   *** *********   **  ***   **      *    * *

mouse
atcatttactggccatttgcatctccagcttattaattggacgctttttgtcactgtttggagttcgttatggatcccaggtcttcatctcccgttggatgtgtaacggacagaggctttctctcatcctgtaggtggtctctcctcactgatgtgggtttgcctggccatgctgaagccttttagcttccagcatcagctctgacatcccttcctgtgcaattgacctttctgcagtcccagcttagacgtctcttaatgttttctctggcagcttcagggttcccaggattctactaagatcttggctgtgctgtgaattgagcttgtgtgcactgtgagagactgtccagtttcattcttctgtatgtggatatgcagttttcctagcaccacttgtgaaagaggctctctttcagtgtaacatgtctggagcgtgtgggtttgctcggtcagtcctctattctgtccactgctctacagacctgtctgcggtactactgcgctgtcttgtttctatggctttgtggtataggccagtgcttccagtattgctcttgtttgctttgcgttgaattgactgtgaaagggtctcttgtggcacccaacccaagaactgccgctgcctgtagcagctgtttaaatagtggtgcacacagcaaaggggctttatcgcctttgggggggctgtgcagtgccccccagagtgaatcagtaggttctacagtcctggggaaaaacaaaacaaagagtcttttgtgtccatatgcaagttgggtgtgttctctagttcttggaattggttgcattgactctgtagatgacttttggtggcatagccattctcactgcctaagccatgaccacaggctctacttatcctccagtgtctacttcaaattttttcaatattttagtttttatattaataggtcattcaccttaggctttgggggttttgctttgtgtttatttttgttttgctattgagaatgggattttttttttcccttgacttttttcttggcaattttgttattagtgtgtagaaaagctgctgatgtggggtactaagtttgtttcctgttactttctgaaagttgttctctggtggcagcctaagggtcttctcaggatct

rat
ttttttttttttttttttggttcttttttttggagctggggaccgaacccagggccttgtgcttcctaggcaagcgctctaccactgagctaaatccccagcccc

mouse GCAGATATGGATAATTTCACCACTCTCCCTGGTTGTACCCCATTCACCTC CCCTTCTCC CCACACTAGCTA
rat   ACGGTGATGG TATTCTTTACAATAGCCATTTTGGTTTAGGTAAGACTCCGCCTTTCTCC CCACACTAGCTA
       * *  **** ** * *   ** *  ** *  * **         **  * ** ****** ************
mouse AGACATTGAGTACTCTACTGAACAAGAGCAGGGAGAATGAGCACCCTG TCATCTTTATCTTAGAGAAAATGCTT
rat   TGGCGTTGAATACTCTACTGAACAAGGGCAGATAGAATGGGCACCCTG TCTT        TAGAGAAAATGCTT
       * * **** **************** ****  ****** ******** ** *        **************
```

This mouse-specific type 1 snoRT is inserted in a L1 element in the reverse orientation (underlined). The orthologous region of rat Zbed3 gene contains an ID sequence (green letters).

mouse: chr13:96,432,563-96,434,003

rat: chr2:25,698,999-25,699,361

**38- Primate-specific U3 snoRT (extragenic); parental gene: U3**

**cow	AAGTCTATTTATATCCTCATGGAACATAAATTTGACTGGGATCAATCCTCCTAAAACAAAATTTCCATTAAGAAATGGA**

**human AAGTCTATTTACATTCTCACAGAATGTAAATTTGACTGGGGCCAGTCAGATTAAAAAAACTTTT ATTAAG**

************* ****** *** ************** ** **	***** ** *** ********

gcagcgtccccaacctttttggtaccagggaccagtttcgtggaagacaatttttccacggagtgggggtgggggaaaggttttgggatgaaactgttccacctcgggtcatcaggcattagattttcataagaatcaggcaacctagatccctcgcatgtgcagttcacaatagggttcctgcttctatgaaaatctaatgcccagctgatctgacaggaagtggagctgaggcggtaatgctggcttgccccctgtttctgtggggcttactgtgtggactgtttcctcgggttggggacccctgcattaagaaatgtaaaatgaagattatattttcagggatcatttctatagtttgtcactagggaagttcctctgaatgtgtagagcaccagaaacatgaggaagaggcacagggttctctcctgagtgtgaagctggctcttggcgctgctttcctgcaactgctatttgccattcgtgattgtggagagtcagagggagaggatgatgcagtctgagtggtttccgctatagaaagaaacagagaaaggcaggaagggagggaggaagaagggagggagggaaggaaggaaggagggagggagggaaggaaggaagcagggagtaagggaaggaaggagggaaggaggaaagaaaggagggaggaaggaaaggaagaagggaggaaagaaaggaagaagggaggtagggaagtaagaagtggggagggaggaagaagggaggaaggaagggaggaaggaaggaaaagggggggcaaggaaggaaggaaggaaagaaggaagggagggagggaggagggaaagaagaaaagaagggaggaaggaaggaagagagggagggagggagtagggaaagaaggagggaaggaagggagggaggaagggagaaaagcaggagggaagggagggaggagggaaggaatgaaggaagagagggaggaaggaaggaaggagagaaggagggaaggagggagggagagagggagagaggggaaacaaaagaaaagaaaagaaaaaag

**human AGAGGGACGTAAGGAT TGAATTTTCTCCT AACATCAACCATGTCTCTGAGCTCTAGATCAGGAGAATCCAGATCTC**

**cow	AGCAGGAAGTAATGACCTGAATTTTCTCCTCCTATCAGCAACTGTGACTCTGAGCTGTAGGCCAGGAGAATTCAGATCTC**

**** *** **** ** *************	* ** **** ** ********* *** ********* **********

This primate-specific insertion contains a MER1B element (underlined, nts 37-339) and a full-length U3 copy (blue), followed by a ~500-bp polyA/G-rich tail. It contains no TSD.

human : chr20:16,910,884-16,912,074

cow: chr13:22,953,496-22,953,654

**39- ACA14a is a primate-specific ACA14b snoRT in the POR gene, sense orientation; parental gene TOMM20**

```
dog    CAGCTCAGGTGTTAGTCCCTGGATGCCCCTTCTTTG    TGTAGAACCATCTTGTCTTCCCTTTCCTCAGAACTCAATTTTTCC
rhesus CAGCCCGGATGTTGGCCTCTGGGCGCCCCTTCCTTGACTGTTTCAGACTGTCATGGCTGCTTTTTCCTTCCAGCTCTATTTCCCC
       **** * * **** * * ****  ******** ***    * *   **  ** ** ** *  ******   * *** ****  **

>dog
ctgaggtgttatacctactgggacacgcttgcgaa

>rhesus
tgcacttggcctccccatgcagccatgactgctgggataggtctgccctttctcatagcaggagagcaccttggggcaaggttggcgtgtccgctccttcctgaaccagcacagttcctggcatgcaacaggtgctcctcagctgtttgctgcatgcgtggacataagagtgacagaggcatgtccttcgacatgacgtttttgtacctgaacattgtttagaggcttttccagactgtaggctctgtggaattaggagcctgcctgcctcgccctccctggcatccttagtgctttgggccccacgggccagcggagccctggtgcgagttccaggcctgctttggtgggagtgctcagcgcctttagccacacagagcagtagccttgcaaggctttcggagcacatctccagcacatctaagaatcaggaagcaactcagtttctggaatggatttcacaaagtctgtgaataagagggccccacaggccctactcaacagccttctttcatttattattattattattttaaatacagaagatgctagtcttcaaagaacctctttactctttccccccccctttttttttttcgagacagtctcattctattgcccaggctggagtgcaatggcatgatcttggctcactgcaaccttcacctcctaggtgcaagtgattctcctgtctcagcctccagagtagctagaattacaggggtgcgccaccacacttggctaatttttgtattttagtagagactggatttcaccaatgttggccaggctggtctcgagctcctgacctcaagtgatccacctgcctcggcctcccaaagtgttgggattacaggcgcgagccactgcaccag***tttttaaaaaatttgctgggtgaggtgttacatacctgtactctcagctacttgggaggctgaggtggcaggatggcttgaggccaggagtatgagggtgcagtgaactatgattatgctactgctctccacccagtgtgacagagtgagaccgtgtctcttaaaacaagcaaaccaaaaagatgcaggaacctaagatcctgcagcctgggttccctctgggtgcaagcaacttctcctgcctcttgaggccctgagtatgttgtacaatgacacgatggttgagtagactacaaaaggaaccctgcagggactcgagacaacatggacatggttcacatattccggaagtatttctctagctgtgtagaattttagagctgaggatgatccttgattggtgactagctccatattgctttgtggagaagaaacctgaggcccgggaggtcaagtaacctgcccagattcccacaaccagagcctgcggcatctagactgctcagtcagtacagagctccagtaactaggtgcacttcagttatctggagtaggcgctaataacttatttcctgtcagaggccaggtaataaatattttttaggctttgcatgccaagaagtaaaatctaggatattatatataacaagagaggcaacaagtgtccacattttttgttgatgaaattcaaataaggctggttgcagtggttcacacctgtaatcccagcactttgggaggccaaggtgggagaattgcttgagcccaggtgtttgaggccagcctgggcaacttagaccctgtctctgtattttaaaatatatatttttaaaagaaattcaagtaataacaaataacagttgagtgtaagtttttaatacaggtttgttaataagaagaatggaatgatttttgtagggataacagttttgggccaggtgccaatggctcacacctctaatcccagcactcttggaggctgaggtgggaggatcgcttgaatccaggagtttgagaccagcctgggcaacatggctaaaccctgtctctacaaaaaaatattaaaaaattagccaggcatggtaggtagtatgcacctgtagtcccaactactgtggagactgaggtgggaggatcacttgagcccagaaagttgaggctggagtgagctgcgattgtgccactgcactgcagtctgggtgacaaagcaagacccagtctcaaaaaaacagaaaacggaaaacaattttgtttcattggggttctcagttagtgttcccaaacatcaaaatctgttgcaaatacccacttgtcagtgctcatctgtaatgagattgtataggtgacatctttgaaaatagcttttcacacaaataggtactgccaagttatattaggcagcataaattttgatggagcatattcatcattggaagacagcattcatagaattttttttagattcccctcttgatatttgcctttttgcattattgcattgcagatcagccacttccaattgaaagttagatgagagctgctcagttgtacagttaaatggattttaaatataggaatttccttcatacttagatcaagatctgaaaaatgctgctagggctagaactgtggtttgagctcaaaaaaatgtgcaaagttttctcccttgttttaaccttgacagtctaggaattgtatgacgcagattgacattacttgtgattaatacaaggttaatttcattgaaatgactttaccacaatataaattttgcatataactgctgttttgccttataattttagattggcctcattaagaaacatcaagtctgcagcaaaagccaatttccaaagtcatttagtatttggtaatagtggttgagggcagttctttcttttttctttttctttttttttttgagatggagtctcgctctgtcgcccaggctggagtgcagtggtgtgatctcagctcattacaacctcagcctcccagtttcaagtgattctcctgcctcagcctcctgagtagctgggactacaggcacgtgacaccacagccagctaattttttgtatttttagtagagatggggtttcaccgtgttaaccaggattgtcttgatctcctcaccttgtgatctgcccgcctcggcctcccaaagtgctgggattacagcaccgtgcccggccctttttttttttttttttaaagactaagaggacagtacttttcagttattcttgttcagagaagttttagtattcactctgagctcaaaatagtcacaccagactttaccactgctaagctgctgagctgctgtatggtagaacagttaggatattcagcttctatttttgacaaaaatctttaagaccaacttaggttaagtctgtgagagcaaatactgttgacattcccttgtttgatcacacataatagattcaaattttttgcgtggtatctgctggtgaataatatagtgaacaaccataggctccaactaagtctttttctgccccccacattcattataacacatcttagtggattccacctcagatagtactaaacttttctcaacttctttgaaaatgctcttatttttagttgtttcatgctaattcttcaaacttggcattgactccccaagtaaataacagctgaaaagtattggtaatgtcttgcgattcatcaagagccaaggaattgcattcttaaaccctcttggtggctttcctctaaatgcttccaagatatgagcgaatgctatagaaattgcagggaagtccaaagggctgcgcttctcctgtggctcagtcttatttcatacctgcgacatcttttaagggaaatttgcaagcgggagaattatcaacttaaaaaaaatagccaaggaaacctcgactcttcaaggaagcaaggaaaaccactctccttggaagtgacgtcagaggaggcccagtgcaagtcaggacctcaccaccactgggcggaaccggggaaccccaccccatgtgtcagttcagaccatctggggagcagtaatgaagtactcttccctctccaagctagggtttgtcagcggaggcccaggagggagactgaatcttgcc

rhesus TGACAGCAATGAATTGATACCTTCCTTCCCT ATCAGCAGGGTGACAGAGAATATTTA[]TAGGCTTAAATAAGATTCAGGATCTTA
dog    TGACAGCAGTGAGTTGGCACCCTCCATCCCCCACCTTCATGGTGACTGATAAAAAGAG  AGGATGTTAATAAGATTCAGAGTATAA
       ******** *** ***  *** *** ****  * *  ** ****** ** ** *        *   * ************  * * *
```

This primate-specific ACA14 snoRT (blue letters, TSD boxed; green letters are of parental origin) is part of a complex insert containing multiple repeat elements. Asterisks in the rhesus insert indicate the position of a further 1104bp-long insertion in the human genome. Brackets in the lower alignment indicate the position of an *AluSc* element in the rhesus, but not dog, genome.

rhesus : chr3:50,331,609-50,336,108

dog: chr6:10,282,889-10,283,089

human: chr7:75,406,201-75,411,823 (not shown)

**40- Primate-specific U107 snoRT in the ZNF157 gene, sense orientation; parental gene MAGED4**

```
dog   ATTACAATGCCTTCATAGGGAAATCAATACTTCCTCTATAGTAAAGATTTCATGATACTTGAGTGTC
human ATTACAATGCTTCTACAGAGAAATCAGTAATTCCTTTATAGTGGAGAGTTCCTGATACTT
      ********** *  * ** ******* ** ***** ******  *** *** ********       aaatttttttattactttattattattattttcttagagacagggtcttgcttcatctcccaggctggagtgcagtggcacaatcatagctcattgcagcctcgaactactgcactcaagtgatcctcccacctaagcctcctgagtagcagtgactacaggtgcttgtcatcacgcccagctaatttttttgatttttagtagagatgaggtctatgttgccctggctggtcctgaactcctgagctcaagtcatcctcccgcctcagcctcccaaagtgctgggattccaggcctgagccactgtgcctggctatgtgtttttttgttttttttttttgagtcagagtcttccacctgagtctcccgaatagctgggaccacaggcgcacaccaacatgcctggctaattttttaatttttatttttgtagagacagagtctcattatgttgtccaggctagtctcgaactcctggcctcaagcactcctcctgcctcagcctcctgagttggtgggattacaggtgtgagccactgcatgcagcagttcctaatacttgagtgtcaagaaatcctctggggtgtgctcagagcagggggcctgaagaatgcttcttctgtttacaacacacccaataggaatctggggtcattgtgacggggggcacaaaacttgtgacctctctatgaacaaatgccccacatgtgaaaa

human AAGAAATCTTTTGTTAAATCTTCATACGGATGATGGAAACAATA
dog   AAAAAATCTTTTG    ATCTTCATATGGATGATGGAAATAAGA
      ** **********    ********* ************ ** *
```

This U107 snoRT (TSD boxed) is part of a primate-specific insert that also contains two *AluJo* elements (underlined).

human: chrX:47,132,352-47,133,170

dog: chrX:40,919,450-40,919,556

**41- Primate-specific ACA67B snoRT in the JTV1 gene (sense orientation); parental gene: ODC1**

```
dog   TTTGAATTTTGCCTCCATTGTATTACAGGAGTTTGTTCGTTATTAAGAGTGAACATATAAAATAA   AGTCTGCATCTAAAAAAG
human TTTG      GCCGTGAGTGT TCACATCAGTGCATTCATTATTACGAGTGAAAGCGTAGAATAAGAAAGTCTTTATCTAAAAAGG
      ****      ***   * *** * ***  ***   *** ****** *******    ** *****   *****  ********* *

ctgtcatgcatgggtttggattatgacaggcccatccccctggacctctcatagtgccccatgccagagcaaactgtggccccgaaccattgcctggcttctgtacccgtgggccactggcactgaagagggttacacagtgggaata

human AACAAGGCTGTCAGTCTGAGCACTTCAGTTAACGCCTAGTATTTTAGCATCTCCCCATCCCTCATCATCAATGATATCTT
dog   AAAAAGGCAGTATGTTTTAGCCCTTTAGTTAATACTTAGTTTTTTAGATGTTGCCTGCTTGTCATCATCAATGACATTTT
      ** ***** **  ** * *** *** ******  * **** ******    * **      ************* ** **
```

This ACA67B copy has no TSD.

human: chr7:6,022,948-6,023,254

dog: chr6:14,454,649-14,454,811

**42- Primate-specific ACA12 snoRT in the GMEB1 gene, sense orientation; parental gene: POLA.**

**mouse TAAAGTTATGAAGGAG**

**human TAAAGTTATGAAAGGA**

************** ***

gctgggtgcggtggctcatgcctgtaataccagcactttgggaggccgaggcaggcggatcacgaggtcaggagattgagaccatcctagctaacacagtgaaaccccatctctactgaaaatacaaaaaaaatttagtggggcgtggtggcgggctcctgtagtcccagctactcgggaggctgaggcaggagaatggcgtgagcctgggaggcggagcttgcagtgagccgagatcgcaccactgcacttcagcctgggtgacagagcgagactctatgtctcaaaaaaataaataaataaaaagttatgaaaggagtggacatttatttttattcagttttttctcaaggtgaaggtaactgtttgtagatgtcctagagaaatattgtagctttctgttcaccctttgcaactaaaaagcatggactgttccactactgagatttaaaaaataaagttatgaaggga

**human AAAGTCTGGTGTAATACAGTAGCAAGTGGTGGTATAAATAAATAAGTAGCAAAAATTACTTTCCTTTGAGGTGAGGAGAGTTGTGGAT**

**mouse AGGGTCCA TGGAACATAGTAGCAAAGGG	AATCAAT GTAAGAAGGGTTCATTGACTT GGGGTGAGGTGAGCTGT	T**

*** ***	** ** * ******** **	*** *** *** **	** ** *** * ******* *** ***	***

This primate-specific snoRT includes an *AluY* element (highlighted in yellow) and an ACA12 Type 1 snoRT (blue letters, ACA box mutated to AGA) with a triplication of the common the insertion sequence (boxed).

human: chr1:28,888,429-28,889,003

mouse: chr4:131,518,335-131,518,424

```
43- Rabbit-specific U109 snoRT (extragenic); parental gene: TMEM167 orthologue

mouse  ATTACTAACTTGCTTTATTTATTCTAATATAAACTTTACTGCTTCTTTCTGGGG TAATCCCCAGATGAAATCAGATGCTTTT
rabbit ATTGCTATATT CTTTATATATAGTTTGCATAACTGTAATGCTTTTTTCTAGTTCTTCTGTCCACATAAAATCCTGTGCTTTT
       *** ***  ** ****** ***  *      **** ** ***** ***** *   *  *  *** ** *****   *******

tcatgcatgtggaaatgccttctcattccttttggagcaggactgcatccataacatgctacattaataaaggagttctcagggctgccaaccttacagtagaggttgagtggtagtatatttctcctacacaagagaaaaaaaaatcctgtgctttt

rabbit CATGTAATTTTATTTATTGGATGTAGTGTTAGATTAAGGGATGGTATGTGGTTCCTCCAATAGACAAATAATCCTATTAATTGTTGG
mouse  TTCACTATGCTGTATAATGATTGGAGCAATAGTTGCAGGTATGGTGTATGTTGCCCCCAATACCCAAATAAACTTATTAAATGTTGG
             **  * * ** **  ** **   *** *  *** ***** * ** * ** ******  ******* * ****** ******

rabbit: scaffold_162633:11,400-11,726
mouse: chr13:85,996,184-85,996,352
```

**44- Armadillo-specific U109 snoRT in the PPP3R1 gene (sense)**

```
dog       TAAGATTCTT    TATTTTATTAGTAC   CTTGTATAATATAAATTGAATATTGTAGGCATTTTCAACCATTAA
cow       TAAGATTAT    CTGTTATAATACTT TGTATAGTATAGTTTAAATTAAATAT GTAGGAACTTTGAACTACTAG
armadillo TAAGATTTTTGTTCTATTTTAATACTTCTATATTATGTAATGTAAATTGAATAT GCAGTCATTTTCGGCTATTAA
          ******* *     * ** ** ** *      *  * ** * ****** ***** * **  * ***   * * **
```

#### aaaagagagtcctttgcatgtggaaatgtctgttcctaattcttttaggaggaggaatcatatccataacatgatacattaaaataggagttctcaggggctgccaattacagaagaggttgagtggtagtatatttctcctacattaaaaggggggggggcaaaat

```
armadillo  AAAGTTG  TCTTCCCAAAGCTATCCCCCCCAAAA TGGGAAAATAACATGAGTTGCAACTTGGCAGAGCAT
cow       AAAAGATAGTTCCTCCCAGAGGTATCCTTACAAAAAGTGGAAAAATAGCATGAGTCTCAACTTGGCAAAACAT
dog       AAATGATAGTTTTTCC AAAGGTATCCTTACAAGA   GGAAAAATAACATG  TTTTAACTTGGCAAAACAT
           ** * *   *  *** * ** *****   * * *   ** ****** ****  *   ********* * ***

armadillo : scaffold_8161:4,473-4,783
dog : chr10:70,414,114-70,414,249
cow: chr11:48,136,014-48,136,156
```
