# Supplementary material for: Mammalian Small Nucleolar RNAs Are Mobile Genetic Elements
Source: PLoS Genet. 2006 Dec 8;2(12):e205. doi: 10.1371/journal.pgen.0020205 (PMC1687206; doi:10.1371/journal.pgen.0020205)
Supplement: Supplementary file 5 [file pgen.0020205.sg002.doc]

**Supplementary Figure S2: examples of species-or lineage-specific Type 2 snoRTs.**

The site of insertion was determined by inter-species genomic alignments, only parts of which are presented. Sequences of the snoRNA copy and of the downstream intron from the parental gene are in blue and green letters, respectively. The retroposon TSDs and the snoRNA ACA motif (always located 3 nts upstream of the 3’ end of the mature form) are boxed. Genomic coordinates given are for the sequences shown, not the retroposon itself, and thus encompass a larger segment than those given on Table 2.

**1- Human-specific ACA59 retroposon in the FLJ10847 gene; parental gene: VPS13D**

```
chimp AAAAAATATCTTCATATACTTGTCTGATTATTTCTTTAAGATTCGTAAAAGTG
human AAAAAATATCTTCATATACTTGTCTGATTATTTCTTTAAGATTCATAAAAGTG
      ******************************************** ********

ggcccagggtatgttcacggggcgatgctgccctcccagctggcccatgggtgaccctgggaacattaactgcctcacaacgt
ttgtgcctcagttacccgtagatgtagtgagggtaacaatacttactctcgttggtgataaggaacagctaacacaacacaca
gggtttttctgctcccaaaatgggattcttcagtgcaccaaatcgaagaaagcactggagctcacgctggtttctccattgtt
tctccttaacagtggaaactgtaacttttgttctagtagcatctagcatgctcaataattatttgtaaataaaagttatactt
tccctccaaaaaaaaaaaaaaaaagattcataaaagtg

human AAACAAAGGTACGATTTTATGTCTTTTGAATATATTTCCATATGCTTTCCAATA
chimp AAACAAAGGTATGATTTTATGTCTTTTGAATATATTTCCATATGCTTTCCAATA
      *********** ******************************************
```

This snoRT is composed of the ACA59 sequence and 189 bp of downstream intronic sequence from the parental gene. Polyadenylation probably occurred at a cryptic polyA signal (bold).

human: chr17:19,401,411-19,401,887

chimp: chr19:19,729,561-19,729,667

**2- Primate-specific ACA36 snoRT in the AAK1 gene; parental gene:DKC1**

**dog	CCTTCCTTTTTATTTCATATTCTTCTCTTGGCCTTTAAAGAAGGAAGACTCTCC**

**chimp CCTCCCTCTTTATTTCATATATTTCCCTTTGCCTTTAAAGAAATAAGACTCTTC**

***** *** ************ *** *** ************ ******** ***

caaagtgttgagttcagtccagggcagcttccctgttctgttaattaaactttgggacattgaaatgggctaggggagatgat

tgggtagaaagcattattttattcatttgcctcccagcctacaaaaatgcctgcttgggtctaatacatcaacagttaaagat

gcctggaag[*]agacaggactaaagaggctgaagagcttggactagcagacaacttactttcaattttaggaagtttggggt

ctgatgggctgagatacaaagaaactgagttattttcatacatgcctgaaaaaaaaatagaaacaagactcctc

**chimp T CTTTTGACATTCACAGAAGAATATTGTTTCTTTAATGCTTTGTCT**

**dog	TCCTTTTGACA	CAGAGGCAT TGTTTCTTTAATGCTCTGTCT**

*** *********	**** * ** *************** *******

This snoRT contains the snoRNA copy and the downstream intron, minus the 3’-most eight bp. The asterisk marks the position of a human-specific *AluYb8* insertion in the parental DKC1 gene that occurred after the retroposition event.

chimp: chr12:71,896,067-71,896,486

dog: chr10:71,377,770-71,377,864

human: chr2:69,600,442-69,600,861 (not shown).

**3- Primate-specific ACA36 snoRT in the ASCC1 gene, anti-sense; parental gene: DKC1**

**dog	CAGAAGCATTATTCATAATAGCCCACAAATGGAAACAAGTCAAATGTTC**

**human CAGCAGCTTTATTCATAATATCCCATAAGTGGAAACAATCCAAATGTTC**

***** *** ************ **** ** ********* ***********

cagtggtgaaactcaactatttttaaaaaaacataaaaatactaatgacagaaaagaaaaatactttaaaagaaccggccgggtacggtggctcacacctgtaatctcggcattttaggaggccaaggcaggtggatcacttgaggtcaggagtttgaaaccagcctggccaacatggtgaaaccccatctctactaaaaatatacaaaaattagccaggcatgttggcacatgcctataattccagctaaccaggaggctgaggcataagaactgcttgaacccagggagcagaggttgcagtaagccaaggttgtgccactgcactccagcctgggcaacagagcgagaccccgtcttaaaaagaaaaaaagaaaaaaaaaagaaccaaatggaacttcaagagataaaaaaataaaagagctacccaggcacggtggatcacacctgtaatcccagcactttggtaggcctaggcaggcagatcacttgaggccaggagttcaggaccagtcttacaaatatggtgaaaccttgtctctactaaaaatacaaaaattagccaagtgtggtggtgtgtgcctgtaatcccagctactcaggtggctgaggcacaagaatcacttgaactcaggaggcggaggttgcagtgagccaagatcgcaccactgcattccagcctgagcaacagaatgagactctgtctcaaaaaatatataaaaatcaaaaatcaaaattgataattccatcaaatgggattaacaaaagattagacacttcaaaaaaaaaaatcagtgaacttgaagacacatctatagaaactatacaacctgaagcagcctgggtgaggggatcagtcatacccaaacctcagcattataaaatatacccatgtaacaagcctatacatgtacctcctgaatctaaaatagaagctgattccaaagaattgagttcagttcaaggcagcttccctgttctgttaacgaaactttgggacattgaaatgggctacgggagatgattggttcaaaagcattattctattcatttgcctttttttttggagacagagtctcactctgtcaccaggctagagtgcggtggcacgatcttggctcactgcaacctccacctccctggttcaagcaattctcctgcctcagcttcccgagtagctgggactacaggcacgcaccaccacgcccagctaatttttgtatttttagtggagacagggttttaccacgttggccaggatggtctcgatctcttgaccttgtgatccacctgcctcagcctcccaaagtgctgggattacaggtgtgagccaccgcgcccggcctatttgcctctcatcttacaaaaatgcctgcttgagtctaatatatcaacagttaaagatgcctagaag[*]agatgggactgaagaggctgaagacttactgagcttggactaacagacaagttacattcagttttaggaagtctggtctgatgaactaagataaaaagaaactgaattattttcatacatgg**ttgcttttcag**aaataaaaacaaaatagaagctgaaatttaaaaaacaaacagattttaacattataaaattggaaaaacaaaaattttgaacaataaaggaatatttaatttttttctttttttttcatgaaccatacatgagacttaaggaatatttaactatttttggaaaactgaagcagagaaaaaaaaacgtctggacagaatctcagtaacttgtgcaccaatatcaagtgattttttttttctgagatggtctcactctgttgcccaggctggagtgcagtggcaccatctcagctcattgcaaccttcacctcccgggttcaagcgattcttgtgcctcagcctcccgagtacctgggattataaacgtgtgccaccacacccagctaatttttgtatttttagtagagatggggtttcaccatgttggccagggtggtctcaaactcctgacctcaagcgatccactcacctcagcctcccaaagtgctgggattacaggtgtgagccaccgcacccggcctccagtattctgatacacatgtaatcagggttccagaaggagatgagaaaaaaaggaaagaaaagacattcgaggaaataatgactgaaaattatacaaaactggagtgaaacagcaagaaaggagactaggaagttctaggccctcattcccccatggacatatcaaataacaacatacagaccaaagtagcttaggaagaaccctaaaaaccagttaagaagttgcaggaaccaaacatacacctaaccatgaaaaggtcacacttcaaagtggtagaaaactttgtgccattttcattcatccttgccccactcaatctgtcccaccttgtcccaccactgggaagaagctacccaatttccagctcttccctcaggatggagcaaaaatagaactagcttcaatgttctggcttgtgtggggctgcctgagggactggttgctgtctcacctgacttggagggctaatgggaatggtggcatagtctggatatcaggatggaggccattgaaagcagtggcaggtgccacggcagggttgtttaacaacaacaaaaaaaggctagatactatatgggataaatatatataaggaaaaaagtctaaagaggactgtattatttttcaattaagtcaataatataaaattcagaaagggtatgaaaatgttcctgctaaaagaggttgaagagacataaaaacttaagctgggtatggtggctcacatctataattccaacattttgggaggctgaggccagaggatcacttgaggccaggagcttgagaccaacctggtcaacatagcaacaccctgtctctaaaagaaaaaaaaaaaaagagagagagagacaaaaaactaaatgcaatgcctgaccttaccctggatcttgtactgaggggggaaaatataaaggacagtatcagatcatatgccattaaagcttatactttcttatggtatattccttatactatctttatacaaatttttctacattgaaattatttccaataaaaaaaaaaggctgccagataagaatgacaaaaaatttcttttttttttttttttgagacagagtctcactctgttgcccaggctggagtgcagtggtgcaatctcagctcactgcaacctccacctcctaagttcaagcgatcctccctgcctccgcctcctgagtagctgggattacgggcacccgccaccatacagggctaatttttgtatttttgtagagatggggtttccccatgttggccagccttgtctggaactcccgacctcaggtgatccacccacctcagcctcccaaagtactgggatgacaggtgtgagccactgagcccaggcaacaacaaatttcttattagaaactatacctggccaggtatagtggttcacacctgccatcccagcactttgggaggctgaagcagttggattgcttgagcccagagaagtttgagaccagcctgggcaacacagcaaaggcccatctctacaaaaattagctgggcatgatggtacagacttatagtaccagctactcgggaggctgaggcaggaagattgcttgagccccagaggtggaggttgcagtaagctgagattgcaccactgcacttcagcctgagtatctgagtgagaccatgtctcaaaaaaaatgtttttcaataaaaaataaaataaagaaactataaatgccagaagacagtagaattatatgttcacagtcctagaagaataaactacttagaattctataatcagtaaaaatctttcaaaaaagaaggtgaattaaaaaatttttcaaacaaataaaagctgagagaaattatttcccacagatctgaactataagaaatgttaggctgggcacagtggctcatgcctgtaatcccagcactttgggaggctgaggagggcagatcgcctgaggtcaggagttggagaccagcctggccaacatggtgaaaccccatctctactaaaaatataaaaattagccaagtgtggtggcatgcacctgcagtaccagctactcgggaggctgagacaggagaatcgcttgaaccggggaggcggaggttgcagtgagccgagactgcaccactgcactccagcctgggtaacagagccagactccatctcaaaaaataaataaataaataaaaccctcaatccaaaaaaaaaagcggtacaaagaagagatgaacagaaacaaacaaaaaagaacaaagaagaccaaaaaaaaaataaagaagagacagaaagaaacaaaaaataagatggtagatttaaactcacccatagctagaattacattaaacgtaaatgttctatctactccagttaaaaggtagagatgtcagactgcaatttaaaaataagaaacaatacctaactatatgctgtctaaaaagaaacctgttttgaatataaagatacaaatatgttaaaagtaaaaggatagaaatagatatatagtgtaaacactaatcaaaagaaggttagaatggttatattaacatcaaaaaagtagaccagaacaaggaacattatcaggaatagagaaagatatttcataactataaaaggatcttatcaagaaggtgtggaaaaaactggtagaactgaaaagagaaatagatgaatccacagttacaggtggagatttcaacactcctctctccgcaactaaaagaacaagtagatacaaaatcagtatggatacagaggttgcagtgagccaagatcacggcacttcagcctgggtgacacagcaagactctatctcaaaaaaaaaaaaaaaatttataacacaatagtaagaaacaaaaaaaattttaaataagcaatatcacgcactttttaaaaaaaggttaaaatggtaaattttatgttatgtatatttcaccataaaaagaaaagctaaaataccatcaacaacaacaaaaatagacaaaatatttgaacaggtacttcacaaaggcagatacacaaatggccaatgagcacatgaaaagatgcttcgcagtatcagccatgagggaaatgtatataaaaaccacaatgagctaccatgatgagatgcctactagagagggttaaattaaaataagatagacaccactaagtgttggtaagaatgtggagcaacctaaactctcacacatcatttgtagcagtgtaaaagggcacaaccaccttgggaaaagatctgacagtttcctattaaactaaacatacacttgcacataatccaacaattacactattaagtatttacccaagaggtataaaaacatatactgaacacaaagatttctgcacaaatttataatagccaaagactagaaaccatccaaacatctatcagagttgaatagataaagaaatctccagctataaacaatccaaacatct

**human ATCAGGGTTGAATGGATAAA	GTGGTACATTCATGCAAAGAAATACCACTCAACAATAAAAAG**

**dog	ATCATGGAGGAATATGTAAACAAAATGTGGTATATCCATACAATGGAATATTATTCAGCCATAGAAAG**

****** ** ****	****	****** ** *** *** * **** * *** * *** ******

This ACA36 snoRT (blue letters, TSD boxed) is embedded in a complex array of repeat sequences (underlined). It differs from the previous one by the insertion of an *AluSc* element (TSD highlighted in green) in the snoRNA sequence. Moreover, the entire downstream intron is included (green letters), up to the splicing acceptor site (bold). The asterisk marks the position of a human-specific *AluYb8* insertion in the parental DKC1 gene that occurred after the retroposition event.

human: chr10:73,609,634-73,615,244

dog: chr4:26,063,375-26,063,491

**4- Rat-specific U17A snoRT in the Grm8 gene**

**mouse CTGCTTGCAGGACTAAACTTAATGATTAGATAAGACCATTCTGGATTTTCAGTATTTAACCTCTTCAACAT**

**rat	CGGCTTGCAGGACTAAATTTAAGGATTAGATAAGACCATTCTGGATTTATAGTATTTAATAGTCTCGTCGA**

*** *************** **** ************************* *********	** ***

tccaacgtggataccccgggaggtcactctccccgggctctgtcctagtggcgtacgggagcatagggtaccacccgatgatgtacagtccctttccacaacgttggagatgaagctgggcttcgagtctgcgcctgcatattcctacgacttctcagagtcctgtggaccgtgactgaggagacaaaccatgcaggaaacagtcttaaatcgcaatctgggggtgggtctgcgtactttaagcgtttaatttactgacggagtgtctaaaaaaaaatagtctcgtcga

**rat	CATCTT TTTGAGTATCCCCCTCCTTGTTTCTCCTAGTGTCCCTAGGAGATGCCCAAGCCTAGTATGCACAAGTCTACACCGAGCTCC**

**mouse CTTCTTGATTTAATAACATCTTCCTT	TCTCCTGGTG	CCCAA	CTGCACAAGGCTGCT GAGCTCC**

*** **** ** * ** * * *****	****** ***	*****	******** ** *	*********

The U17 host gene could not be localized in the rat genome. Green letters in the snoRT align with the intron downstream of U17A in the mouse U17 host gene.

rat: chr4:54,438,401-54,438,847

mouse: chr6:27,638,201-27,638,334

**5- Cow-specific ACA16 snoRT in the TFE3 gene; parental gene: EST CB463184**

**dog CCATGACAATTTCCCAAGGTCTGAGAT	TGAGTTGGAGTGTTCCAAGAATGCTGACTTGG**

**cow CCATGACAATTTCCCAAGGTCTGAGAAGACTGGGTTGGAGTGTTTCAAGAATGCTGAATTGG**

****************************	** *********** ************ ******

tggcccttatcaaagctgcagctgcttccatatagctgctgtggtcaaaaaggagcccaaagtgacagttttccttgacggtcgcagttctgtttgctgtaactgatctgcaacatttcaggaaaacacagttcccattgtacttgccatttgtcaggtacaaccagagacctttattaaaactgacgttttcagaggctaccggaaatgttccattgatggggtcttacatgcaagaataaggcttttgagaagtggcttgcagaggccaagaaccagtatgcagatgcagatatgggtgggttagatacctgtgctggggaaatccaggggcaagtggtgcagattgtggtcattctttgggtctttgccctgaaaagcttaaggcatcagcccaaaggcgagtcaaaaaccttgttctgaaacccagatgaatggaacgagtaggtcattcaggacaaaaaaaaaaaaaaaaaagaatgctgaattgg

**cow AATGTCATGCTAGGAGGGGCT	GAAATGTTGCATTGGAGCTGATAAAGACCTGAGAAGCGTGGTTTTC**

**dog AATGTTGTGCTAGGAGGGGCTAACGGGCTGGAATGTTGTGTCGTAGCTCACAAAGATCTAAGAAGGGTGGTTTTC**

******* **************	* ******* * * **** * ***** ** ***** ***********

A second copy of this snoRT is present on scaffold2565:13,612-14,119 (extragenic). The parental cow ACA16 host gene (EST CB463184) is, as in human, also the host of ACA44, ACA61 and HBII-420, but its cDNA sequence has no similarity with that of the human host gene.

cow: chrX:26,120,309-26,120,928

dog: chrX:42,135,921-42,136,054

**6- Cow-specific ACA14 snoRT in the AM035258 EST (human C8orf38 gene); parental gene TOMM20**

catctctagtaaatcatttcctattgccacatttgaaagagtatctgatttattgcattcttaaaccctcttggtagcttcaacctgattgcttccaggatatgagtgaatgccataggaattgcaggggagtccaaagggctgtgcctctcctgtggctcagtcttatttcgtacctgcgacatctcctaagagagatttgtaagggggaaattgtgtcactgaaaccatggtggtctttgaatcttgcagatatactcattctcagtatacaatgtaagagcaaatttgttgcagcaagtatttttgtaacttatctagtttgctcagtcaaaatagtttctgaaattaaaatttccacttaagtt**attaaa**gcccagaaagtacaaagacatgattctcatatttaatactttgtggt

This snoRT comprises the snoRNA sequence and 196 bp (out of 7kb) of the downstream intron from the parental gene. As observed for the ACA59 snoRT presented in **1**, polyadenylation probably occurred at a cryptic polyA site (bold). The insertion point of this cow-specific snoRT could not be precisely localized due to multiple insertions of repeat sequences in the corresponding intron in other species.

cow: chr14:43,049,296-43,049,716

```
7- Rodent-specific U107 snoRT in the 2410187C16Rik (human C8orf32) gene; parental gene Maged2

cow   TCCTGAATGTCTCTAGGTTGGAAAGACAAATGTCTTCTCAAAACTCTGTCTGAGAACAT
mouse TTCAGAATTCCTGTAGACCAGACCAACACGCGTCTTGTCAGAACTTGGATAAAGATGTT
      * * ****  ** ***    **   ***   ***** *** ****  *    ***   *

mouse
atggggtgtgctcagagcaggtggcctgaagaatggcttctctgtttacaacacacccaagaagaatctgggaccattgtaatgaggggcacaagcagttccctctctgtgaatggatcccctacatgtgtctcccctattcatccacatggcttccaggcaaacccagtgggacaggactcagtaccgggtatgctccagtacttagctggggggtgggggatgtccagaccatagttggttcatgtgtgagtagtccatgggtggtctgaggatgaagactgaacctgagttggttgtttcttcaccacctggatccccaggagtgcacgcaggcctttaccctctgagctatt

cow
ccaggttaaaaaaaattttagctactacatgttatatgtcatattgaaacagttcacatgtta

mouse TTGC CTGCGCTGAATTTTTCTTAAAAGGGTGGATGAG GCCTG
cow   TTACTCTGTGCTGCACATCCCATAGAATTATTAGCGTTCTCCTG
      ** * *** **** *  *  * ** **   *    *    ****
```

This rodent-specific copy of the U107 snoRNA and 3’-most intron from the parental gene has no TSD and polyA-like tail. Compared to the cow genome, its insertion might have been accompanied by a ~ 60-bp deletion.

mouse: chr15:57,976,638-57,977,094

cow: chr14:7,763,731-7,763,896
