# Supplementary material for: Mammalian Small Nucleolar RNAs Are Mobile Genetic Elements
Source: PLoS Genet. 2006 Dec 8;2(12):e205. doi: 10.1371/journal.pgen.0020205 (PMC1687206; doi:10.1371/journal.pgen.0020205)
Supplement: Supplementary file 6 [file pgen.0020205.sg003.doc]

**1- Primate-specific U70 snoRT in the COBLL1 gene; parental gene RPL10**

```
dog    TTGGCAATAATCACAAAA  ATAATCCCTAACACTGAGAC TTTGGGCCCAAGTTACATAATGAAAAGTCACTACC
rhesus TTGGCAAGAATTACAAAATAATTCTCCCGTACACAGAAACATTTGGGACCAA         TG AAAGTCATTACC
       ******* *** ******  **  ****  **** ** ** ****** ****         ** ******* ****

tctgcagccaattaaacctactgagttcctttccttaggggggcccagtgtgcagtggctgcaaacagcagcctccttggtagtgtatgcaacctgtttcttatataggttgctctaagggaccttggtgataggcctttcagatgtatgttcatgtctctgaccttgcactacctcaatgtaagctccaaacaggcataggaggtgtctttggaaagccccagggtactgtggccagggttcacattggccaagttatcatgtccatccgcaccaaactgcagaacaaggagcatgtgattgaggccctgcacaaggccaagttcaagttctctggctgccagaag*atccacttctcaaagaagtgagacttcaccaagttcagtgctgatgaatttgaagacatggtggctgagaagtggctcatcccagatggatgtggggtcaagtacattcccaatcatggacctctggacaagtgccgggccctgcactcatgagggcttccactgtgctgccccctcttaatactcaccagtaaattctacctcctctccaaaaaaaaagaaaaaaaaaaaaaaaaagtcattacc

rhesus TGTC    ATTTGTTGGCTATTCTTGGAAAGAAATCTCACAGGTATACTAAACAG  TGC
dog    TGTCTGCTGTCTGTTGACCAT CTTGGAAAGGAATCCCACAGGAATACCAAACAAAATGC
       ****     * ***** * ** ********* **** ****** **** *****   ***

The snoRT contains the U70 snoRNA sequence, plus the downstream intron and two processed exons from the parental gene. Note the mutation of the ACA motif to ATA.
Rhesus : chr12:28,324,757-28,325,470
Dog: chr36:12,898,531-12,898,662
```

Human: chr2:165,251,905-165,252,610 (not shown)

**2- Primate-specific U70 snoRT in the PHLPPL gene; parental gene RPL10**

**a: sequence of the human snoRT (chr16:70,289,183-70,290,150)**

taggtagcatgattaatcatgggattaagagagcatgtaaatcctctgcagacagttaagccaactgagttcctttcctcagggaggcccagtgtacaatggctgcccacagcagcttccttggtagcgtactcagcctgtttcttgtatgggttgctctaagggaccttagagacaggcctttcagatggatgttcatgtctctgactttgtgctatccccagtctaggctccaaacaggtatgcgaggtgcctttggaaagccgcagggcactgtggccgaggttcacattggccaagttaccatgtccatctgcaccaagctgcataacaaggagcacatgattgaggccctatgtagggccaagttgaag*ttccctggttgccagaagatcccacatctctaagaagtggggctttaccaagttcaatgcagatgaatttgaagacatgggggctgagaagcagctcatcccagatggctgcggggtcagttacatccccagtagtggccctctggacaagtggcgggccctgctcttgtgagggcttccactgtgctgccccctcttaatgctcacc**aataaa**tcctactttctgggccgggcgcggtggctcacacctgtaatcccagcactttgggaggctgtggcaggcggatcacgaggtcaggagattgagaccatcctggctaacacagtgaaaccccgtctctactaaaaatacaaaaaattagctggacgtggtggcgggtgcctgtagtcccagctactctggaggctgaggtgggagaatggtgtgaacccgggaggcagagcttgcagtgagctgagatggtgccactgcactccagcctgggcgacagagcaagactccatctcaaaaaaaaaaaaaaaaatcctacttcctgttcaccttaaaaaaaaaaagagagagagagagagaaagagcaaacctcttatttgcgt

The snoRT (TSD highlighted in yellow) is composed of the snoRNA sequence, the entire downstream intron and the processed last two exons of the parental gene. The *AluY* element inserted in the snoRT in the human and chimpanzee genomes is underlined, with the corresponding TSD highlighted in red.

**b: sequence of the rhesus retrotranposon and determination of the insertion site**

**dog	GTAAAAGCTGTGGAAATAACAT	AATTGACTAAAGATGAGTAGGTAGCATGATCAATCA GAGATTAAGAGAGCACATCAGT**

**rhesus GTAAAAGCTGTGGAAATAACATTTCACTAAAGTCAGGTTGAGTAGGTAGCATGATTAATCATGGGATTAAGAGAACATATAAATCCT**

************************ * * * * * * ***************** ***** * ********** ** ** * ***

ctgcagccaattaagccgactgtgttcctttcctcatgggggctcagtgtgcaatggctgcacacagtagcttccttagtagtgtactcagcctgtttcttgtatgggttgctctaagggaccttggagacaggcctttcagatggatgttcatgtctctgactttgcactatccccagtctagGCTCCAAACAGGTATGCGAGGTGCCTTTGGAAAGCCCCAGGGCACTGTGGCCGGGGTTCACATTGGCCAAATTATCATGTCCATTTGCACCAAGATGCAGAACAAGGAGCTTGTGTTGAGGCCCTATGCAGGGCCAAGTTGAAG*TTCCCTGGCCGCCAGAAGATCCACATCTCTAAGAAGTGGGGCTTTACCAAGTTCAGTGCAGATGAATTTGAAGACATGGTGGCTGAGAAGCAGCTCATCCCAGATGGCTGTGGGGTCAATTACATCCCCAACAGTGGCCCTCTGGACAACTGGCGGCGGGCCCTGCACTCGTGAGGGCTTCTACTGTGCTGCCCCCTCttaatgctcgcc**aatgaa**tcctacttcct[]gttcaccttaaaaaaaaaaaaagagagagagagaaagagcaaaccc

**rhesus	CTT ACTTGTGTTGGAGAAAGTGGCATACAGAGTGTGTGATTTGTTAGGGAAGT**

**dog	GAACTCTTGATTTGTGTATGAAAAAAAGGAGTCCAGAAAGCGTGATTTACTAGGGAAGT**

***** * ****** ** *** ** * **** * ******* ***********

The brackets in the snoRT sequence indicate the site of insertion of an *AluY* element in the human and chimpanzee genomes. A potential TSD is boxed.

rhesus: chr20:70,963,744-70,964,484

dog: chr5:80,487,432-80,487,570

**3- Primate-specific U70 snoRT in the UPS34 gene; parental gene RPL10**

**a: sequence of the human snoRT (chr2:61,497,432-61,498,041)**

tttggctgtaagattacggaggtcctgcagccaattaagctgactgaattcctttccttatgggggtccagtgtgcaatggctgtaaacagcagcttcc[]ttggtagtgtatgcggcctgtttgttgtataggttgctctaagggaccttggagacaggcctttcagatgtatgttcgtgtttctgaccttgcactaccccagtgtagggtccaaacaggcatgtgaggtgcctttggaaagccccagggcactgtggccagggttcacattggccaagttatgtccatccataccaagctgcagaacaaggcgcatgtgattgaggtcctgctcagggccaagttcaag*ttccccggctgccagaagatccacatctggaagaagtggggcttcaccaagttcaatgccgatgaatttgaagatatggtggctgagaagcggctcatcccagatggctgtggggtcaagtatgtcgccattcatggccctctggacaagtggctggccctgcactcatgagggcttccactgtgctgccccctcttaatacc**aaTaca**ttctacttcctgtccccccccccaaaaaaaaaaagattagggaggttaataa

Brackets indicate the position of an *Alu* element in the rhesus monkey genome downstream of the snoRNA H box (ACAGCA).

**b: sequence of the rhesus snoRT and determination of the insertion site**

**cow	GTCATCAT	TTTTAATTGCTTCATCTCAGTAATATAGCTGTCACTTCTTTGTAAGATTT AAG**

**dog	GTCATCATATACCATTTTTAATTGCTCTATAGCAGTTGTATAGCTGCCATTCCTTATTTT ATTTGTAAG**

**rhesus GTCATCAT TTTTAAGTGCTGTTTAACAGTCATATAGCTGTCTCTACTTTGGCT ATAACATGACGGAGGT**

**********	****** ****	* **** ******** * * *****

cctgcagccaattaagccaactgagttcccttccttatgggggccccagtgtgcagtcgctgcaaacagcagcttccggccgggcgcggtggctcaagcctgtaatcctagcactttgggaggctgagacgggcggatcacaaggtcagcagatcgagaccatcctggttaacacggtgaaaccctgtctctactaaaaaatacaaaaaactagccgggcgaggtggcaggcacctgtagtcccagctactcgggaggctgagccaggagaatggcgtaaacccgggaggcggagcttgcagtgagctgagatctggccactgcactccagcctgggcgacagagctagactccgtctcaaaaaaaaaaaaaaaaaaaaa*aa*acggcagcttccttggtagtatatgcagcctgtttgttgtatgggttgctctaagggaccttggagacaggcctttcagatgtatgttcatgtttctgaccttgcgctatcccaatgtaggctccaaacaggcatgcgaggtgcctttgcaaagccccagggcactgtgtccagggttcatgctggccaagttatgtccacctgcactaagctgcagaacaaggagcatgtgattgagaccctgcgcagggccaagttcagg*ttccccagctggaaaatccacatctcaaagaagtggggcttcaccaagttcaatgccgatgaatttgaagacatggtggctgagaagccgctcatcctagatggctgtgggttcaagtacatccccagtcatggccctctggacaagtggctggccctgcactcatgagggcttccactgtgctgcggcctcttaatactcacc**aataca**ttctacttgctgtccccccccccccaaaaaaaaaaagattagggaggt

**rhesus TAATAACACATGTTTGTTTACCAGATTATCATCATTTTGTTAATTT	AATG	ATTGGGAATGGAATAGGAGGAAAACT**

**dog	ATAAGTTACATGTTTGTTTAAAAGGTTATCATTATTTTATTAAT	AGTGCAATTAGAATGGGAGGAAAACT**

**cow	TAGTTATGTATGTTACTTTAACAGATTATCATTATTTTATCACTATTGGAATGGGAAATTGGAATGAAATGGGAAATAAAAT**

******* **** ** ******* ***** * * *	* *	* * *** ***	*** ***

The TSD marking the insertion of an *AluMacYa4* element is highlighted in red.

rhesus: chr13:61,599,497-61,600,543

cow: chr11:39,466,651-39,466,793

dog: chr10:64,620,204-64,620,342

**4- Primate-specific U70 snoRT in the RAB30 gene; parental gene RPL10**

**cow	GAACACATCAGAGTAAC AGTACCCAGATTAAAAAACTGAATATAATCAT GTCTCAATAAGCCTGGTTTTT**

**human GAACACATCAGGGTAACCAGTATCCAGATTAAGAAACAGAACGTAACCAACATCTCAGTAAGACTGATTTTT**

************* ***** **** ********* **** *** *** **	***** **** *** *******

ctgcaaccaattaagccgacctagttcctttcctctttggggcctggtgttcaatagctgcaaacagcagcttccttggtagtgtatgcagcctgtttcttgtatgggttgctctaaaggaccttggagacagccctttctaatatatgtttatgtctctgaccttgtactgtccccagtgtaggctccagacagtggaggtgcctttggaaagcctcaaggagccttgatcagggttcatactggccaagttgtcttgtctatccacatcaagctatagaaccaggagcatgtgattgaggccctccacagggccaagttcaag*ttccctggtcaccagaagatccacatctgaaagaagtggggcgtttcaaagttcgatgcagatgatttttttttttttgagacagagtttcactctgtctcccaggctggagtacagtggtgtgatctcagctcactgcaacctccacctcctgggttcaagcaatactcctgcctcagcctcctgagtagctgggactacaggcacatgcaaccacgcccagctaatttttgtatttttttggtagagatgcggtttcaccatgctggccagggtcgtctcaaactcctggcgtcaagtcatttgcccactttggcctcccaaagtactgggattacaggtgtgagccaccgtgcccagccagtgcagatgaatttgaagacgtggtggctctggtatgagaagtggctcatcccagatggctgtggggtcaaatacatctccagtctgggcaagtggcaggccctgcattcacgaaggcttccactgtgctgctccctcctaatgcccaccaataaatcctacttcctgtccagaaaaagaagattctgattttt

**human AACTGTATAGAACTTACATCCAAATGGTGGTTGTCCTTTTCAATGCCAAGTTAGGAGTGATCCAAGAAAATCAATATTATTTT**

**cow	AACCATATAGAACTGATATCTAAATGATGTTTGTCTTTTTCAATACCAATTTTTGAATGGCTCAGGAAAATCAGTATCATTTT**

***** ********* * *** ***** ** ***** ******** **** ** ** ** ** ******** *** *******

In the human, chimpanzee and rhesus genomes, this U70 snoRT is interrupted by the insertion of an *AluSx* element (TSD highlighted in red) in its portion corresponding to the second exon of the parental gene.

human: chr11:82,429,324-82,430,360

```
cow: scaffold815:77,395-77,547

5- Mouse-specific U70 snoRT in the Snx5 gene; parental gene Rpl10

rat   CTGGAATGGAGCCCTCAACAGGCAAATGT
mouse CTGGGATGGAGCCCTCAGCAGGCAA  GT
      **** ************ *******  **
mouse
gtgcagctaattaagccgaccaagtcccttttccccatagggacttggtgtgcaatggttgcaaacagcagcttccttggtgtggtgtatgtagcctattgtctatataggttgcttcaagggaccttggagacatttctggcagttggtaatcaagcctctgatcacaatgctgtccctgatctaggccccagacagggattcgtggtgcctttgggaagccccagggcacattggccagatcatcatgtccatccgaaccaagttgcagaataaggaacaggtgatggagattgtggctctgcgcagagccaagttcaagttccctggcagccagaag*atccacatctcaaagatgtggggcttcaccaagtttaatgcagatgaatttgaagacatggttgctgagaagcagctcattcctgatggctgtggtgtcaaatatatccccagtcatggtcctctgaacaagttcagagccctgcattcctgaaggcttccacagtgctctcctataccctaccaaatcatgatcaataataaatcttgcatcaagttcaggggggaaaaaagaaagaa

rat
gcagccagtttgcatttgcacatgaggcaaatcactggcgt

mouse GAAAAGGAAGCCAAGGATAATTTCCAGAATTATAGT
rat   GAAAAGGAAACCAAGGACAATTTCCAGAACCATAGT
      ********* ******* ***********  *****
```

This snoRT has no TSD; compared to the rat genome, its insertion might have been accompanied by a 41-bp deletion.

mouse: chr2:144,001,969-144,002,610

rat : chr3:132,694,186-132,694,291

**6 : rat-specific U70 snoRT (extragenic); parental gene: Rpl10**

**mouse CATTATCAGTCTACCATGAGGATAACAAAGTGATCATACTTTTCCCAGAATTTATACTTAGAAGAGTTTGTAAAAAAAAAAAAAAAAA**

**rat	CATTATCATTCTATTTTGAGGAAGACCAAGTCATCATATTTTTCCCAAAAAGTATAGTT AAGAGTTTCTAAAACA**

********** ****	****** ** **** ****** ******** ** **** ** ******** ***** ***

tgtgcagctaattaagccgatcaagtccctttccccatggggactttggtgtgcaatggttgcaaacagcagcctccttggtagcgtatgtagcctattgtctatatgggttacctcaagggaccttggaaacagttttctggcagttgaataatcaagtgtctgatcataattggtgtccctgatctaggctccagacaggtatgcgtggtgcctttgggaagccccagggcacagtagccaggattcacattggccaagtcatcatgtctatccgaaccaagctgcagaataaggaacatgtgattgaggctctgcgcagggccaagttcaagttccctggctgccagaag*atccacatctcaaagaaatggggtttcaccaagttcaatgcagatgaatttgaagacatggttgctgagaagcagctcattcctgatggctgtggtgtcaaatatatccccaatcgtggtcctcttgacaagtggagagccctgcattcctgaaggcttccacagtgctctcctataccctatcaaatcatgttcaat**aataaa**tctcacatcaaatctgcttaaaaaaaagagtttctaaaaca

**rat	GCTAAGATGATGTAGCTGAGAACAGTTTAGGAAATGAAAGAAACTCTTTGAGGAACCCATTTTCTGATGAACAA**

**mouse GCTAAGGTGATGTGACTGAGAACAGTT AGGGGATGAAAGAAACTTTGTGGGCAACCCCTTTTATGATGAAAGA**

******** ****** ************ *** ************ * ** * ***** **** ******* ***

rat: chr3:20,616,514-20,617,260

mouse: chr2:40,382,100-40,382,260

**7- Mouse-specific U65 snoRT in the Lats2 gene (anti-sense); parental gene Rpl12**

```
rat   TAAAAACCAAGGAAATCCACTGGTACTTC          AAAAATAAAAATGTCC
mouse TAAAAGGCAAGGAAAACCACTACTACTTAAAAAAAAAAAAAAAAAAAAAAAGTCC
      *****  ******** *****  *****           ***** ***** ****

agccacccgccactgcacctgaccagtattgttggctggtgcaatccagtggtgagctgacagtgagcccagcttttaggaaacagggctgtcctacatgtggatggctctgtgccgaaagcgtgggaacagcctagcgataggtaaaatggaaccatgatctcagaagacaggggaagctgtaaaaggccctgtgctttctttctgcagTTAAACACAGTGGGAACATTACTTTTGATGAGATTGTCAACATTGCCCGGCAGATGAGACACCGGTCTTTGGCTAGAGAACTTTCTG*GAACTATCAAGGAGATCCTGGGTACTGCACAGTCTGTGGGCTGCAATATGGATGGCCGCCACCCTCATGACATCATAGATGACATCAACAGTGGTGCAGTGGAGTGCCCAGCT*AGTTAAGAAGCAACAAGAAAATATTTCAATAAAAGACTATCTGATAACCAGAAGAAAAAAAAAGTCC

mouse TGTGGAAAACATTTCTAGAATCCATAATCTAGTTAGTATTTCAGTAGTTCAAAGAAAATA
rat   TGTGGAAA CATTCCTAGAATCCTTACTCTAGTTAGTATTTCAGGAGTTCAGAGAAAATG
      ******** **** ********* ** ***************** ****** *******

This snoRT contains the U65 snoRNA sequence, the downstream intronic sequence and the processed last three exons from the parental gene.
mouse : chr14:56,651,859-56,652,450
rat : chr15:36,744,011-36,744,114
```

**8: dog-specific U107c snoRT in the MAML2 gene (sense); parental gene: MAGED4**

```
rhesus TGGCCAACTGGAGAGCACAAAGAGCAATTTGTGTTTCAAACACAATGGGG
dog    TGGCCCACTGGAGGGCAAAG       TTTGTGTTTAAAATATGATGGGGA
       ***** ******* *** *        ********* *** *  ******

aaaaaaataaaataaaatatgatgggagcatggggtgtgctcagagcagggagcctgaagaaatggctcctctgtttataacacacccaagaggaatctgggtttgttgtgacaagaggcacaaaatgtgtggccttcctatgaacagacaccctacacgtgtcccaccttgcatctccacatgtcttctgaataggaccaacagcctaaggctttgtaggcctcaatcctggaggcagaaaccctgggagttgaggcatgggtgaggtccaagacagcccacccctatcccccaccctgtgcaggcactgggactggaaaggggccacctggagccagtggtgcaacaacagatctgcctggctggaagcaaatgtttatcatcctctcacactgagatacctggaataagaagattcccagtagcagcccacccaactatgaattcctctagggcctgtgagccctccacaagaccagcaagatgaacgtgctgagattcatcacccag*aatcaggactgagaccccccagaagtggaaggctcatttattggaggctgtgatgattcattcaaggcaatagatgtggaaatggctgaggcacatgccagggccaagatgagggcccagatgaacattgaggatgaagctctgattggatggttggagctgggatgacatatgggtgtagcttctgacctggtaggatgacagagattttagcagtcgctgggctaggattcccttggctcctgggccacataccatcagtacattttgaatggcaaccatgccaacaggagagctgcttggagagctggtattagcagtggcaccagtggtgtggccaacaccagctccaccctccagaccagaaatgctgccaggacaagaagaacagcaacttctcctggat*ccaacagttatgatccaaaactagatccaaaactcaccataccagagtacctcctcagatcccttcttaccacagtactctgggcagcttctacctgcctatccaggatggcacgcaagatgaaactggctttgcccttcctgtgtttattgtgtgcttttgtcctgttttcatgtttttgctatcagtcttacattaacgttgcaaaatttttaaaaaaggtaaaataaaatattatggga

dog     GCAAAACACACCACATCTATGATCCAGGTACACTTATAGTGTAAG
rhesus  GCCAAACACACCACATCTGTAGTCCAGGTACACGTACAGTGTGAG
        ** *************** *  *********** ** ***** **

Note the triplication of the TSD.
rhesus : chr14:94,551,467-94,551,561
dog : chr21:8,148,912-8,150,159
```

**9- Cow-specific U17C snoRT in the TBCD gene; parental gene: U17HG**

**dog AAATCACCTCCTGGCAGACACCCTGGGCAGCTGTACATCCCTGTTGGTTTGTTACCTTGCACTGTCTGTTGTTCCAGTGTCTGAAAATTC**

**cow AAATTATTTTCTAGTGGACAGCTTGGGCAGCTATAGAGTCCAGTTGGTTGGTTACAAGTCGCTGTCT TCCTAGTACCTGAAAATAC**

****** * * ** * **** * ********* ** * ** ******* *****	* ******	* * *** ******** ***

tgagtt**ggccaaaaagttcatttggtttt**ttcctaagtgggctctagtagtgcttggttgtctttaacttcatttgaaacagttttgttagattatattgtgacagctgtcttaccagcatccattaaaaaaatttttatcaaaattggtgagtatttatatagccatttaaataatgaagatggaagaaaaacaacatttttggcatactgtgctttattatttcaagaaaggtacacggtggaaataaaaaaaaaggtttgggcagtgtatggagaagggactgtgactgatctgtcagaaatggtttgcaaagtcccatgctggagatctcttactggacagtgctccatggttaggtagaccagttgaagttgatagcaatcaaattgagacattaattgagaatagtcaacgttataccatgcaggagatagcctacgtactcaaaatatccaaatcaagtgttgaaaatcatttgcacttattttcttatatcattaaataatctacaatgttaaaaaaaaaaaaatcatttgcaccagcttggttatgttattgctttgtttgggtcccacataagttgtgaaaaatttttttcttgaccatatttgtgcatgtgatgctctactgaaacataatgaaagggttctgtttttaaaaacaaattgtgaccaatgtggatacccggggaggtcactctccccgggctctgtccaggtggcgtacgggagcatagcgttctgcccggtaatgtatagtccctttccacaacgttggagatgaagccgggccttgagtctgcgcctgcatattcctacgtcttctcagagtcctgtggacaatgactgaggaaaaaaaccacgcagaaaacagctctgaagagcaatctgtctggatctgggttgctccgaagtgaattttttttaattgttttaacctgttttgctgtgcagttgggttaaggttttccctcttttgcag**cattttgtttgggattccaaatacaactggatttgag*****cattttgtttgtgattccaaataccactggatttgag***gggagaagaggagcctcaccaacttcgcagttagcaatgctttggagtctttctttgacagggaaattagtaaagtaaaaattaactccaggcaaaactactggagtggaacccattcctttctccatggggatcttcctgacccagggattgaacctagatcctactgcattgcaggtagattctttacattctgaaacacaaagtaaaatgagtaatacattcattgaagataagcctgggaaatttggaagtgtataggtacgatatttaacccctggaactgcaccttaaaacccatgagttctagtgttctgtaatgtataagaaatgccaaagaccatatgggcaggttttagagtatagtgacctggaaggctcaaaagaccttttcaggtttcctatctggtgaaaacttgatgtaatgacactaagatattgacctctttatctatcattcttaaaagtggaaagtaggtgcccatgagaatcagcatgtcttaaagaaatccatcaatttctaatgtaatcgtgaactttaataaagatgactatatgtgtaaaaaaaaaaataaaaacaaattgtgatgggcagtgaagtggatactgcacagtaatgtggaacaaaagagattatggggcaagtgaactgaatgaacgacaccaaaggaggtgatgctgtatttatggtgggattggaaggagtcctctcttaatgagctgcttctggaaaaccagacaattaattctaacaagtactgctcccagttagacctactgaaagcagcattggatgaaaagcatctggaattagtcagcagaaaacacataatcttacatcaggataacacaaggttgcatgtttctttgatgatctggcaaaaaccgttatagcttggctgggatgttctaatgcatccgccatattcaagagacactgcaccttttttttggaaaaaaaatttatttttgacttgtgctgggtctttgttgctgtgcaggcttttctttagttgcggcctgtgggcttctcatttcagtggcttctctcgttgtggggcttgggctctagggcacacgggtttcagtagttgcggatctcgctcaagagcacaggctcagtagttgtggtgtatggacttagctactccatgcattttgggatcttcctggatcagagatcgaacctgtgtttcctgcattggcagtcagattctttactgctgagccaccagggaagcctcaacattgcacctttggatttctgtctatttcaaaggtctttacagaattctcctgatggaaaagatttcagattccctggaaggctgtaaaaggcacctggaacagttctttgctcaaaaagataaaagagttttgggaagatgaaattatgaagttgcctgaaacatggcaaaatgtagtggaacaaaatggtgaatatgttattcaataaaatccttggtgaaaatgaaaatatgtcttttattttgacttg**aaaaccaaaggaactttttggcc**gcc

**cow CCAGTACAATTGTTGGGTTGTCTAATTGTTGGTGGAAAGGTAGTTCCATATCAGTTATTCCTTTATAACTGGAAGTGGAAATCCTTT**

**dog ATCACATACTTGTCTAGTTTTCTAATTTTTGGTGGTGGAACAGTTCTATATAATCTATTCCTTTATAACTAGGAGTGGAAATCCTTT**

*** * ****	*** ******* *******	***** **** * *************** * ****************

This cow-specific retrotransposon is an *Oamar1 Mariner* element (nts 2-1213, highlighted in yellow, terminal inverted repeats in bold characters), in which three different retroposons have inserted: a 44-bp AT-rich sequence of unknown origin (highlighted in green, TSD AAATCATTTGCAC), the U17 Type-3 snoRT (TSD TAAAAACAAATTGTG), and a CHR2A, tRNA-glu family element (highlighted in red, TSD ACACTGCACCTTT). In the snoRT, the U17 sequence is in red letters (100% identical to the parental copy), and the downstream intron and the processed exons from the parental gene (U17 HG) are in black and blue letters, respectively. Asterisks indicate exon-exon junctions. The duplicated exon 2 (see text) is in bold characters.

cow: chr19:44,121,592-44,124,346

dog: chrUn:11,960,209-11,960,385

**10- Rhesus-specific ACA62 snoRT in the NKIRAS1 gene; parental gene: U104/ACA62HG**

**chimp TACCCCCCATAATGATACTATGTTATAGTT	TTTTTTAAACT AGTTA**

**rhesus TATCCCCCCAAGTCATACTATGTTATAGTTGGTTTTTTTTTTTTTAAACTCTAGTTA**

**** ***** * * ****************	*********** *******

cgcgctgtctttgagcccccgccgagcctcctcgtggcgccgggggtcaatctgcagcgctagagcatgtgcttgcgcataactggggccgcttggccccccgcgggcggcccttttaaccgcgagcgacaattagtaacctgactgtccggctgctaggctgggcaattttccagggattttgccacgacaggcctcaacccgtaataccaaggctggaatggcgtgtgagggggacggtgtctgacggggatacgcggactgctgcgtagtgagtgcccctctgtgctcccatgcgttccccagccctccccgagtgcatcgcgcgcgttgtgcctttgccatgagttcgacataagtcccaagctgtcgtgtctgccatcagacgcgcaggtttagttgaaggcagcttggaatggcgtctgcagggctgctgtgtacttgacataaacggattcagtgtggaactgcatgagttaactcctgactaaaagggtaatcgacctgtttacaaacaaccaccatgcaggagtatgtgtgacaagcatgggactaaagcttcgggttaggttaatctgtcactcctaaacagttaaaaattatagggcggttgtgaagatgaattaggttttatatatagttggggacaacatagacacctagttacagtccagattctatggttggtaaaagatcttacacaagggctccagtgaaatgtgcatcagttctgagtacgaggcagcagtggtgggcaaggatgcacgtatgcgctcactacatgggcattcgatgaatgaacgtgtcccgaatgaagataactagacgatgaagggtataaactgcagcccaggagcaccactgccctaccccctcagttatgaactttcactggattctttaaatgtgggctgaagataatggcacataatttgaagggatgtggtgaggatggctttgcagccttaggactgggcagtgatccaaaccagttaagtgaaagctgccattgccaggtgagggccccggtgcttggtactcacttgtgcccctatctgaacaggattcagtagaaatgacttagatgcttatctagtatttgacatgagatatttattttgtgaaacaatgcaagcgattttaaatccccacatactatttgattaagtgcaacatggtacagtcatgtgtaatctctttacacgatagtgcatctagtccttagcctttagttattgcacaacaattataaagaccagtgaccaggacacgcagactgtgacaggcagattggcctacacaatgaaaaatcaacagtacaccaactggaatggtcaaacaatttaagtcaaatgttttaatggtgc**aattaa**aataatcaaggattcaaaaaaaaaaaacaaaacaaaacaaaaccaaaaaaaaaactctagtta

**rhesus CAGTTTTCCTAAACAACCTATATACTGTAGAATGATTTGTTCCAGATGCCTTACATGAAA**

**chimp CAGGTTTCCTAAACAACCTAAATACTGTAGAATGATTTGTTCCAGATGCCTTACATGAAA**

***** **************** *****************************************

This snoRT contains the ACA62 snoRNA sequence (small red letters) and 1257 bp that are 99.8% identical to the sequence located downstream of the parental ACA62 copy on chr16 (green letters). The TSD is boxed. This snoRT allows one to propose a structure for a conserved host gene for both U104 and ACA62 snoRNAs. A potential polyA signal is in bold letters. Note the AAAC repeats in the A-rich tail.

rhesus: chr2:166,351,291-166,352,843

chimp: chr2:24,854,593-24,854,698

**11- Mouse-specific ACA48 snoRT in the Gsr gene (sense); parental gene: Eif4a1**

**rat	AGTAGTTACCATGCTGTGCGTCAGATCTCTTGAAATA**

**mouse AGCAGTTACCATGCTGTGCATTAGATCTCTTGAAATA**

**** **************** * *****************

cacccctggcgatgggcaaagagacaccttatgtgtggtgttgttcttatatcagccaggggcagagcagatggcaggcctttgttcatcctagcttgatgcctagcttggtggccaaagctgtttcatgcccagggcacataaaatcctgacgcacgcctgttgctactattcaagcctagtgactactgtgaagcaagcatctgggcaccgatacacaaaaggtggttgtggcattaggagacagcatgggtgcctctcatcatgcctgtattgggggtaccaatgtgtgtgctgaggtgtagaagctgcaaatggaagctccccatatcatcgtgggcacccctgaccgggtgcttgatgtacttaaccggagatactgt*cccccaaatacatcaagacgttcgtactggatgaagcagttgaaatgttaagccgtagggacaaggatcagatctatgatatattccaaaagctctacagcaacacacag*gtagttttgttactgtctgctacaatggtttctgatgtccttgaggtgaccaagaagttcatgagagaccctattcggattcttgccaagaaagaagagttgaccctggagggtatcagccagttctacatcaacatggaacgagag*gactggaagcttggcacatcgtgtgacttgtatggaacactgcccatcacccaggcagtcatctctatcaacaccagaaggaaggtggactggctcactgagaagatgcatgcccggtatctcacggtttccgccatg*catggagatagggaccaaaaggaacgagatgtgatcatgagggagttcctgtctggctctagcagagtattaccactgacctgttg*gccagaggcattgatgtgcagcaggtctccttagccatcaattacagccttcccaccaacagggaaaactacatccacag*aactggttgaggtggctgatttggccgtaagggtgtggctattaacaaggtgactgaagacaagaggactcttcgagacattgagactgtctacaacacctgcattgaagagatgcccctcaacgttgcagacatcgtttgaggggctgtcctgctacctggccctagaccagggttcaatcctgggggcagaggcctgaggaacagctgggggtgggggaggagccaagggatggacatcttatttttgttttgtttcaggtttttttttctctctctctctttg**aataaa**tgtcactttttgaggcaaaataaataataaataaacaaataaataaataaaaataaatctcttgaaata

**mouse GTTATCCTAACCAAACTTTTATGTCCTTTGACAAGACTTTGGAAA**

**rat	GTTATCCTAACCGAAATCTTACGTCCTTTGACAAGACTTTGAAAA**

************** ** * *** ******************* *****

This snoRT contains the ACA48 snoRNA sequence (red letters), 11 bp of the downstream intron from the parental gene (green letters), followed by 60 bp of unknown origin (black letters), and the processed last 7 exons of the parental gene. Bases deleted in the snoRT from Figure S3-12 are underlined. Note the repeat of the AAAT motif in the polyA-like tail.

mouse : chr8:35,157,699-35,159,085

rat : chr16:62,259,475-62,259,556

A similar mouse-specific ACA48 snoRT in the 3’UTR of the Cenpc1 gene is truncated in both 5’ and 3’, but presents the same rearrangement of the intronic sequence. In this case, a full length L1 element is inserted at the 3’ end of the snoRNA sequence (chr5:87,079,967-87,086,588).

**12- Mouse-specific ACA48 snoRT in the Itfg3 gene, anti-sense; parental gene: Eif4a1**

**rat	GAGTTCCGGAACAGCAGGGCTACTACAGAGAGAAGCCCTGTCTTGAAAAACAAACAAACA**

**mouse GAGTTCCAGAACAGCCAGG GCTACTAAGAGAAACCTTGTCTTGAAAAACAAACAAACA**

********* ******* **	**** ****** ** ************************

cccctggcaatgggcaaagagacaccttatgtgtggtgttgttcttatatcagccaggggcagagcagatggcaggcctttgttcatcctagcttgatgcctagcttggtggccaaagctgtttcatgcctagggcacataaaatcctgacgcacgcctgttgctactattcaagcctagtgactgctgtgaagcaagcatctgggcaccgatacacaaaaggtggttgtggcattaggagacagcatgggtgcctctcgtcatgcctgtattgggggtaccaatgtgcgggctgaggtgcagaagctgcaaatggaagctccccatatcatcatgggcagccctgaccgggagcttgatatacttaaccagagatactgt*ctcccaaatacatcaagacgttcgtactagacgaagcagttgaaatgttaagccgtagggacaaggatcagatctatgatatattccaaaagctctacagcaacacacag*gtagttttgttaccgtctgctacaatggcttctgatgtccttgaggtgaccaagaagttcatgagagaccctattcggattcttgtcaagaaagaagagttgaccctggagggtatccgccagttctacatcaatatgg[*]cacatagtgtgacttgtatggaacactgcccatcacccaggcagtcatctctatcaacacagaaggaaggtggactggctcactgagaagatgcatgcccggtatctcacggtttcctccatg*catggagatagggaccaaaaggaatgtgatcatgagggagttcctgtctggctctagcagagtattaattaccactgacctgttg*gccagaggcattgatgtgcagcaggtctccttagccatcaattacagccttcccaccaactacatccacag*aattggttgaggtagctgatttggccgtaagggtgtggctattaacaaggtgactgaagacaagaggactcttcgagacattgagaccgtctacaacacctgcattgaagagatgcccctcaacgttgcagacctcgtttgaggggctgtcctcctacctagccctagaccagggttcaatcctgggggcggggggcgtaggccagaggaacagctggagggggggcggggaagggagccaagggatggatatcttatttttgttttgtttcaggtttttttttctctctctctctttg**aataaa**tgtcactttttgaggcaaaataaacaataaacaaacaaaca

**mouse AAAAGAAAAACAAACAAATACACAAACGAATCTAGAGTGAAAACAGGTTCTGGGGACAC**

**rat	AACAAACACACACAGAGAGA AATGAAATCTGGAGTGAAGGTAGGTTCTGGGGGCAC**

**** *** *** *** * * * **	***** *******	*********** *****

This snoRT is identical to that presented above, except for a 23-bp deletion encompassing an exon-exon junction (brackets).

mouse: chr17:25,951,046-25,952,417

rat: chr10:15,492,202-15,492,317

**13- Rat-specific U69 snoRT (extragenic); parental gene: Rpl39**

**mouse CTCTGTGCTAGGTC TGTACTTGTGAGGGACTTCTCCTTGTTGTTAAAATTTATACT**

**rat	CTCTGTGCTAGGTCCTGTACTTGTGAGGGACTTCCCCTGGTTGTTAAAATTTATACT**

**************** ******************* *** ********************

gagcaggtagcaattacagtgcttcattttgtgggagtactgccattaatcctgctgaaagaaaagccatgttgatgtttctgatttttgcatttattggagtaaaaatcatgacacttgcatggggacagttcagagactggttggcttagctctgcaatttggagttttggaattaag/gtacaactctaagagaagacactggaggagaacgaagctgggtctgtaaggagtcacactacggccagactgaggatttctactgaatcgccgtcatcgatcagtccgaccagatggatttcaacatttttaacctagagatttacttgtgtctgaactaatttgtccagt**aataaa**atttagaacctttcaaaaaaaatttatact

**rat	GACCAGC GTCCTTGTGATGCAAGCTATTTTC**

**mouse GGCCAGTTATCCTTGTGATACAAGCTCTTTTC**

*** ****	********** ****** *******

This snoRT contains the U69 snoRNA sequence (red) followed by 47 bp of the downstream intron (green) and the last exon (blue) of the parental gene. The slash indicates the position of a 315-bp deletion of intronic sequence from the parental gene. The alignment with the sequence of the parental gene shows that the retroposed RNA species was spliced at the level of a cryptic donor site (ttttggaattaaggtaggatc).

rat: chr20:44,368,796-44,369,270

mouse: chr10:39,886,115-39,886,202

**14- Rhesus-specific U99 snoRT in the human AK126715 mRNA gene (sense orientation); parental gene: U99HG**

**human CTAACTTTGTCAGTTCACCAGAGTTAAAGAAAGATAATTCGCCTTGGGGAAAAGTCTATGTAATTACCTACTTTTAAAAAGGACC T**

**rhesus CTAACTTTGTCAGTTCACCAGAGTTAAAGAAAGATAACTCACCTTGGGGAAAAGTCTACGTAATGACCTACTTTTAAAAAGGACCAT**

*************************************** ** ***************** ***** ******************** ***

cctccttttcttggcggggatcgggcttgtggtaccgctccccgtaatgtgcggaggaagagggaaagggctctggccccctcggcgtcatgtcttcggtgccggcggcttcctatccactggttctatcctcaagcgccgggacaccgggaatctcggaccggcacaaccgagatccagctggctcctccatggggttgctcacatgatctcatttgatttcaggtctgcggacgctgtatcccctcttccacttaccgctgcagcc**aataaa**ggccgttcctaccataaaaaaataaataaataaataaataaataaataaataaataaataaaggacct

**rhesus TTCATAATGCCGCTCCTAGGTGTAAACCCAAGAGAAAGGAAACCTACATCTACACGGAGACTTGTACCTAAATGTTCG**

**human GTCATAATGCA CTCCTAGGTGTAAACCCAAGAGAAATGAAACCTACATCTACACAAAGACTTGTACTTAAATGTTCG**

*********** ************************* ***************** ********** ************

This rhesus-specific Type 3 U99 snoRT contains the snoRNA sequence (red) followed by 141 bp from the parental gene. By alignment with the human BU564879 EST, this tail is composed of the downstream intronic sequence (green) and the last exon (blue) of the presumptive U99 host gene (polyA signal in bold characters). Note the repeat of the AAAT motif in the polyA-like tail.

```
rhesus : chr11:129,938,877-129,939,383
human : chr12:127,703,319-127,703,481

15- Human/chimpanzee-specific U70 snoRT in the ASTN2 gene (sense); parental gene RPL10

rhesus CATGCTATAAAATAAAAGAGAAGAGTGTCTTGTCTTGTCCACTCATTGGCCATAA
human  CATGCTATAAAATAAAATAGAAGAGAGTCTTGTATTGTTCACTCGGTGGCCAT
       ***************** ******* ******* **** *****  *******

ttacaaagtgcacactccatgtacagaatgaagtcactgtgctaagtgctaggttatagagggattaataccttcaacaagtttatggttgtgtgtaagagagttgagtaaacaggcaatttcagcagtatgaaatgtatttgagacatttgccagaacccaatcttagctctgatacttaccagctctttgacctggggcaagttatttgacatctctgggcttcagtttccccatctctgaaatagttatgtgagcattctcagcttatacattgctgtgaagaataattgaaatactccagatcatctgcagccaattaagccaactgggttcctttcctcatgggggcccattgtgcaatggctgcaaacagcagctttcttggtagtgtatgcagcctgtttgttgtatgggttgctttaagggaccatggaaacaggcctttcagatgtatgtttatgtttctgaccttgcgctaccccagtgcaggctccaaacaggcatgcgaggtgcctttggaaagccccagggcactgtgaccagggttcacattggccaagttatcatgtccatccacaccaagctgcagaacaaggagcatgtaattgaggccctgcgcagggccaagttcaagtttcctggccaccagaag*atccgcatctcaaagaagtggggcttcaccaagttcaatgttgatgaatttgaagacatggtggctgagaagcagctcatccctgatggctgtggggtcaagtacatccccagtcgtggccctctggacaagtggcgggccctgcactcatgagggcttccactgtgctgccccctcttaatactcaccaataaattctacttcctgtccaaaaaaaaaaaaaaaaagatactccagatcagttgtttagaatggtgctcggtgcatagtaagcagtcaataaatgattgctgtgagtgtgctgaaatatggatatgaacaaatattccaggatcacagaggaaatggcattcaaatgtgctgacgatgtggatgagaacttccctcaagaggagacatttgagctgaatttgaagataagctcatcagtgaacccaggggcaagggaaaggggatgtgtaaaggtcccaaggcaacagggagagaacattgtaggaactgtgagaagtttcaagtggctggcaacaaaggaacatgaacagaaagagcagactgtagaggaagagatgggtcagatcaccagggtcctgggactttatctgggaggccccagggagccactgaaaacttttaactgagaagtgtcatgcccagatagtcttcctcccagaaagatcatttaacatggtggcacaaacttagatcagaaaaaaggcagagagatcaagaatggaagactactctgtgaatcaaaagagagatgcagagctagcatgctccaagagatgatacagcactgatctggaaatgggcctccctgcattttattcaaagctttgctgcagatctgccatttgaattggatgcccttagatggacctcagttttcccatctatcaaatgagaaccaatgatctcagaagcatcatccatcacctacatcctacatccttgaatagggagggtaatgtctgaggtaaggaaatggcaaaaccaggggattgggggtaaagtaagtccctaaacaccaactttcagcagcctgaaatgacagatctgcagtggggtctgaatgacagatctgcagtggggtctttcagaatcaggcagaaaggccctgcct

human   GTGCCTTTAGGCCCAGCAGTCCTGGAGTACAGCAAGCATAGGAGAGAAACAAGCATAGGAGGGAAGCCCTCATGTC
rhesus  GTGCCTTTAGGCCCAGCAGTCCTGGAGTGCAGCAAGCATAGGAGAAAAACAAGCATAGAAGGGAAGCCCTTATGTC
        **************************** **************** ************ *********** *****
```

This human/chimpanzee-specific insert is composed of the U70 Type 3 snoRT (U70 sequence in red, downstream intron in black, exons of the parental gene in blue) inserted in a MIRb element (highlighted in yellow, nts 62-260), and L2 and MIR3 repeats (underlined) embedded in sequences of unknown origin. Only the U70 snoRT displays a TSD.

**16- Primate-specific ACA63 snoRT in the ATP2B4 gene (sense); parental gene: RANBP1**

**dog	GAAATCAAATGGATCCTCACCACTGGATACCTGCTGGGGGCTTATTTCCCACAGGCCAGACGGTGATGGTGATGGCGGTGGGAAGG**

**rhesus TAGATGAAGTGAATATTCTAAACTGCATACCTAGTAGGAACTTATCTCGTATAGGCAAGACA ACAGATAGAAAAGTGGAAAAG**

*** ** ** ** ** **	**** ****** * ** ***** ** * **** ****	* * *	**** ** ***

aggccgggtgtggtggctcacgcctgtaatcccagcactttgggaggccgaggcaggcagatcatgaggtcaggagttcgagaccagcctggccaacatagtgaaacctcggctctactaaaaatacaaaaatcagctgggcgtggtggtgggcacctgtaatcccagctattcgggaggctgaggcaggagaatcacttgaacccaggaggctgaggttgcggtgagccaagattgtgccattacacaccagcctgggcaacaaggcaagactctgtctcaaaaaaaaaaaaaaaaaagaaaagaaaagaaaaagaaaaatggattgcagactcactctgcacctgactatacttccaggcgggtgctttttctgtctgccagataaacattccagggtgctgtggctgtctcacctatccagggcgatgcagctccctggggacacaggtgcttcctggggaggcctgagcacttctccgtgttgctggcctcacatccaactgggcaggcattggagtccgggcactgcctggagctcaccagaaggtgctttacacagtggtcctcagtttgtctctcagagggcttggtcacctctgccgtgaggctggactgcagctgtggtggcaagtgaccaggtgtcactgagcagcctgaccatggtctaggcggtgcttgaatgcatccacttggcttcctttggtcattgagtttcaagaagacagactccaggaggcgaattggc/CAGGATCAGGCAAAAACAATCATGCCGGAAAAGTGGCGGAAAAGCTAGAAGCTCTCTCAGTGAAGGAGGAGACCAAGGAGGATGCTGAGGAGAAGCAATAAATTGTCTTATTTTATTTCCTTTTCCTCTCTTTCCTTTCTTTTAAAAAAAAAAAAATTACCCTGCCTCTCTTTTTTAGTTTGTTTTTATTCTTTCATTTTTACAAGGGACATTATATAAAGAGCTAAATtttaaaaaaagaaaagaaaaagaaaagtggaaaag

**rhesus	GGCTAGGTAGGGAAACACTAGGGTTCCTTTTCCCTCTCTCGAGTTCCTCCTTCCAGA**

**dog	GAGGGCTAACTCAGGAG CACTGGGGCTCCCTGGCTCTCCTTCGGGTTCCTCCC CTAGA**

******* * *** **** *** *** * * *** *** ******** * *****

This primate-specific snoRT is composed of the ACA63 snoRT (TSD highlighted in yellow) and an *AluSq* element inserted with an overlapping TSD (AGAAAAA/GTGG). The slash in the snoRT sequence indicates the deletion of the last 132 bp from the parental intron. The boundary of this deletion in the parental gene (GGTGGCTTGGC/caggcaggagtct**)** does not correspond to a cryptic splicing donor site.

Rhesus: chr1:166,696,171-166,697,322

Dog: chr38:3,408,191-3,408,334

**17- Dog-specific U65 snoRT (extragenic): parental gene: RPL12**

```
rhesus GGCCAAGCTGATTGATGATGTGGTTCAAGTCTTCTATATACTTACTGATTTTCTGTCTACTTTTCCATCGATTACCAAGAAAGGAGT
cow    GGTCACGTTGATTGCTAATGTTGT        TCTATATCCTTAGTGACTTCCGGTCTACTCTTCTATCAGTTATTTGCCTCTCTCT
dog    GGCCAAGTTGATTAATCATGTTATCCAAGTCCTCTCTATCCTCCTTGAGT  CTGTCTACTCTTTTATCAATTACCAAGAGAAGAGT
       ** ** * *****  * ****  *        *** *** **   *** *  * ******* **  ***  ***

rhesus AT      CTTCAACCAAAATTGGGAGTTGCCTATCTCTCCTTGCAGTTATTAGATCAGTTTTTGTTT
cow    CCTTT---------------------------------------------------------------
dog    GTTGAAGTCTTCAACTACGCCTGGAATTTACCCGGTTCTCCCTTCCGTTTGTTT     TATTTGTTT

gtttacataggctccacaaacacagcatgaagcccattgcagggcttgattctgagatcaagacctgagctaacagccacccgccactgcacctgatcagttttctgttggctggtgcaatccagtggtgagctgatagtaaaccccagcttaggaaacagggttgttcttcatgtggatgactctgtgccgaaagcatgggaacagcctggaaattaagatgaggcagcatattacgtggtctgctctggccttgggttgtgatagaacttaggctgtttttcttttctgcagttaagcacagtggaaatatcacttttgatgagattgtcaatattgcccgacagatgcgacaccgatctttagccagagaactttctg*gaaccattaaagagatcctggggactgcccagtctgtgggctgcaatgttgatggccgtcaccctcatgacatcatagatgacatcaatagtggtgcagtggaatgcccagcgag*ttaactacaaaggaaaatatttcaataaaggatcatttgacaaccaaaaaaaaaaaaaaaaaaaaaaaaaaagacctgagctaagatcacgagtcagacgcttcaccgactgagccagccagatgcccct

dog    CAGTTCCATCTGTCTTTGCTGCATGTATTCGGAAGCTCTGTGAGGTGTAGCAATGTTTAGGTCCGACATGTCCTCTTGAT
cow    GAGTTCCACCAGTTTCTGCTTCATGTATTCTGAAATTCTGTTCAGTGCAGTAATGTTTAGATCAGT GTATCCTCTTGAT
rhesus                      CATGTATTCTGAAGTTCTGTTAGGTGCAGCAATGTTTAGGGTTGGTGTGTCCTCTTGAT
                            ********* ***  *****   *** ** *********    *   * **********
```

This U65 Type-3 snoRT is inserted into a SINEC1D element (underlined) possibly retroposed from chr1 (see alignment below). Integration of this SINE element occurred without TSD, and alignments with orthologous cow and rhesus genomic segments suggest local rearrangements.

dog: chr28:24,854,631-24,855,399

rhesus: chr9:110,223,303-110,223,510

cow: chr26:19,204,636-19,204,798

**Determination of the snoRNA snoRT insertion site in the SINEC1D element**

```
chr28  TTACATAGGCTCCACAAACACAGCATGAAGCCCATTGCAGGGCTTGATT        CTGAGATCAAGA
chr1   TAAAGTAGGCTCCA  TACCCAGCATGTAGCCCAATGCAGGGCTTGAACTCACAACCCTAAGATCAAGA
       * *  *********   ** ******* ****** ************          ** **********
chr28  CCTGAGCTAAGATCACGAGTCAGACGCTTCACCGACTGAGCCAGCCAGATGCCCC
chr1   CCTGAGCTAAGATCAAGAGTCAAATGCTTAAC GACTGAGCCACCCAGGTGCCCC
       *************** ****** * **** ** ********** **** ******
```

The sequence of the dog-specific insertion shown above was deleted from the snoRT sequence and aligned with a SINEC1D element located on chr1:109,804,014-109,804,134. The position of the sequence duplicated upon snoRT insertion is boxed.

**18- Mouse-specific U65 snoRT (extragenic); parental gene Rpl12**

**rat	GCAGCACCATATCCCCACACGTGGAATTAAAACAGAAGCGCATTCTTATTG**

**mouse GCAGCTCCATATCCCTATTCCTCTGATAACAATGAAAGCACATTCTTATTC**

******* ********* * * *	** * **	**** ************

tttttcaaatcagccaaaattccagaattttcagaaatgtcactatacctcagctctgccctctccagtctgggagactctgagttgtctcctaagagtgtagaaagccactgcacctgaccagtattgttggctggtgcaacacagtggtgagctgacaataagcccagcttttaggaaacagggctgtcctacatgtggatggctctgtgccgaaagcatggaaacagcctagagatgggtaaagtggaaccatgatctcagaagaaaggggaagctgtaaaaggcactgtgtttctgcagttaaacacagtgggagcattagttttgatgagtttgtcaacactgtctggcagatgagacaccggtctttggctagagaactttctg*gaactatcaaggagatcctgggtacctcccagtctgtgggctgcaatgtggatggctgccaccctcatgacatcatagatgacatcaacagtggtgcagtggagtgcccagct*agttaagaagcaacaagaaaatatttct**ataaaa**gactatctgattaaaaaaaaaaaagggagtgtagaaagcacttaaggtaggtagacctcctcagtcttcactgcctgcctctggtacagcatagcacttgtttggtttgtgcctgggtgaatgtgcacaggggctggggcaggtggtggtggtggtggtggtggtgtatgcatggtatgcaggtatgctcacccaggaaggtgactgtggagac

**mouse CAGAGGCTGACATCAGAAAGGTCTTCCTCAATTGCTCCTCCTCATAATTTTT TAATCCATTACAT TTTTTAGTT**

**rat	CATTGACTGACATCAGGA GGTCTTCCTCAATTCCTCCTCCTCACAACTTT[]CTA TCCATTTCGTATTTTTAGTT**

**** * ********** * ************** ********** ** ***	** ****** * * ***********

Additional sequences inserted upstream and downstream of the snoRT are of unknown origin. Brackets in the lower alignment indicate the position of a rat-specific ID element that has been deleted from the sequence shown. The corresponding TSD is underlined.

mouse: chr11:20,747,163-20,748,038

rat: chr14:101,664,786-101,665,036

**19- Mouse-specific U65 snoRT (extragenic); parental gene Rpl12**

**rat	ACCCATTGCGTTTCACTTCCCAAGAAGATGGCAAGACCCAATCCAGAGTAACTTTATTTTGGTCTTATTTTTCGAAAAA	GATTGA**

**mouse ACCCATTGCGTTTCACTTAC AAGAAGATGGCAAGACCCAGTCCAGAGTAATTTTCTTTTATTTTTGTTTTTCAAAAAAAAAGATTGA**

******************** * ******************* ********** *** **** * ** ****** *****	********

atgggggctggagagatggctcagtggttaagaccactgatagctagctcttccggaggttctgagttcaattcccagcaaccacacggtggcccacaaccatctgtaatgggttctgatgccctcttctggtgtgtgtctgaagatagtgacagtgtactcacaaatgtaaaataaataagtcttaaaaaaataaataaccagccacccgccactgcacctgaccagtattgttggctggtgcaatccagtggtgagctgacagtaagccccagcttttaggaaacagggctgtcctacatgtggatggctctgtgccgaaagcatgggaacaacctagcgataggtaaaatggaaccatgatctcagaagacaggggaagctgtaaaaggccctgtgctttctttctgcagttaaacacagtgggaacattacttttgatgagattgccaacattgcccggcagatgagacaccggtctttggctagagaactttctg*aaactatcaaggagatcctgggtactgcacagtctgtgggctgcaatgtggatggccgccaccctcatgacatcatagatgacatcaacagtggtgcagtggagtgcccagct*agttaagaagcaacaagaaaatatttc**aataaa**agactatctgataaaaaaaataaataaataaataaataaataaaaagaaagtccagtgatagtcccaaagtgggatccagctcaaggggaggtcccaaggcctaacactattactgaggctatgaagcactcacaaaaagggacctatcatgactgccctctgaaatatccaacaagcagctgagtcagatgaagatatttgcacccaaccaatggacagaagcagctgaccctgttgttgaattagggaaggctgaaagaagttgaggagagggcaatcctgtaggagggccaaaagtcttaattaatctggaacccccaagatctctcaaacactagaccaccaaacaggcagcaaacatcagctgatatgaggcccccaacacacacacagtagaggatttcctgttcattcagagatgatgcacctaaccctcaagggactgcagacccctgggagtttagaggtcaggtagagtggggagtggggacatccacgtggagacagaggagtggggaggaggtatgggatgtggaaaagtcggagggtggatggggtggggggataacatatgaaatgcaaaaaaataaattaattaaaaaaaaagaaaaagaaggattga

**mouse	TTGATTGATTGATGCATCTGTGTGAGTGTGCAGTATGTGTGC**

**rat	TTGATTGATTGATATATCTGTGTGAGTGAA	TGTGC**

*************** *************	*******

This mouse-specific, 1271-bp retroposon (TSD highlighted in yellow) is composed of a complete B2 element, the Type-3 snoRT (TSD boxed) and the 3’ part of an Lx3 (L1 family) element in the same orientation as the B2 element. Most probably, the B2-Lx3 element was retroposed first by a template switching mechanism, followed by the insertion of the U63 snoRT.

mouse: chr12:31,844,014-31,845,411

rat: chr6:49,112,995-49,113,087

**20- Primate-specific ACA3-2 snoRT (extragenic); parental gene RPL27A**

**dog	AATCTGGAGATTGAATATGGAGAAATGAGGAACAAGGGTAATACAAAATTAAATT	GTTCTCATATACTAGATTCAACCTTTAAAA**

**human AATCTGGAAACTGAATATGGAGAAAGGAAAATAAAGAATGTTATGGAATTAAATTTGTGCTATTATATGCCAGAATAAACACTCAAAA**

********** * ************** ** * *** * **	*********	* * * **** * *** * *** * ******

**human**

gctaagttcgagctgacactagagtaacatcctgacacagctcttgtcctggtgtgctagagttctcgatgaatctactggtcttgattcactggcaggagcagtgactgtgttagtatgcgtatctgaaacatggcctacctagatcccagaaggtgtgggttgggaggcatctttcctcatactcatcagggagttggtacctgctatggtgtattataagccatttctctgtttttctaggcctactaaaatgttctgcctggggaaggaaaagttcccaaagcagcctatcatcatgaaagccaaattcttcagcagaagagctgagaagattaaaagcgtttatgagtatggggctagtcttggttacttaaagtcacatggggggaggttt**attaaa**tgttaacaaatgctttttaa

**dog**

ttaatttaaaaaataaataaataaaaaaaataaaattaattttaaaaagattattattggggatccctgggtggctctgtggtttggtgcctgcctttggctcagggtgtgatcctggagacccagaatcgagtctcacatcgggctccctgcaaggagcctgcttctccctctgcctgtgtctctgcctctctctctctctgtgtctctaatgaataaataaataaaatctttaagaaacaataaaaaataaaa

**human AAATAGCTGATGTAGAAAATTCTGTCACTTACATTATCAA AAAATAATGAAGTACCAATCTTTTAAAGATGTATAGATCTTTAAAA**

**dog	AAATA	AAAATATTATTATTTACAGTGGCAGCAAAACAGCAAAAGTCCAATCTATTAGTCATACGTAGGACTTCAAAA**

*******	***** * * * ***** * ** **** *	**	******* ***	**	*** *** ******

This primate-specific ACA3-2 Type 3 snoRT has no TSD. A SINE element is inserted at the corresponding site in the dog genome (underlined, potential TSD boxed). Note that a similar snoRT, possibly human-specific, is located on chr16:2,786,093-2,786,545 and corresponds to the s72770 candidate snoRNA described by Washietl *et al.* (2005) Nature Biotech. 23:1383-1390.

human: chr12:83,100,861-83,101,457

dog: chr15:28,873,809-28,874,225

#### 21-Primate-specific U70 Type 3 snoRT (extragenic); parental gene: RPL10

```
Dog   TACCCCCAAAAGATAATATACACACATGCTTGTATATGAAAAGTCAATGGCTAGTAGGATACATATAAAAATATTAAGAGG
Human TACCATAAAAAGGTAATATACACATATGGTTGTATATGAAGAAGAAACAGATAGTAGGCTACATGTAAAAATGATAAAGGG
      ****   ***** *********** *** *********** *   **  * ******* ***** *******  ***  **

```

#### ctgcagccaattgagctgatggagttcctttcctcctggggcccagtgtgcaaaagctgcaaacagcagcttccttggtagtgtacgcagcctgtttcttatatgggttgctctaagggaccttggagacagtcatatggttgttcatgtctctgaccttgcactaccctcagtgctctgaataggtattcgaggtgcctttgaaaagccccagggtgctgtggccagggttcacattggccaagtcatcatgtccatctgcaccaagctgcagaacaaggagcatgtgattgaggccctatgcaaggccaactttaagttccctggccgccagaatatccacttctcagagaagtgggactttaccaagttcagtgtggatgaatttgaagacatgatggctgagaagcagcttatcccaataactgtggggtcaagtatacccccaatcgtgaccctccggacaagcggtgggccctgcactcatgagggcttctactgtgctgccccctcctaatgcccaccaataaatcctacttcctgtccaccttaaaaaaatgacaaagtgt

#### human AGTCACCTCTTGAGAGTAAGAATAG GGCTGGTGTGGAAGT GAGTTTTTACTTCTCACTGGACATATTCCATGTTGGAAAA dog GGTTACCTCTGAACAGTGGGAATGGAATTGGGGACTGGTGTGAAAGTAGGGTTTTTACTTCTCACTTTATATATTTCATGTTTGGAAA ** ****** * *** **** * * ******** **** * **************** * ***** ****** * ***

#### The 3’ end of this snoRT constitutes the 5’ end of the spliced EST DR731413 (bold characters). See Figure S5A.

#### human : chr8:8,856,414-8,857,143

#### dog : chr16:38,483,327-38,483,495

#### 22- Human- and chimp-specific U70 Type 3 snoRT in EST BG260387 (sense); parental gene RPL10.

**dog	TATATATCTGTCAAACTTCCTCTTCAGAAAGCAGAGGTCAAAATAATAAGAATCCTACCAGAAAGGAGCCTGTGTCAAGAAAAT
human TATATCTACGTCAAACTTCCATTTCAGAAAGCCGAGGTCAAAATAATGAG TCCTGGGGAATTCAAACTTGTGTTAAGAAAAT
 ***** * *********** ********** ************** ** ****	*	* * ***** **********

**human**

**gtgtcccagtgtgcaatggctgcaaacagcagcttccttggtagtgtatgcagcctgtttgttgtacgggttgctctaaagggccttggagacagtcctttcagatggatgtacatgtttttgaccttgcactaccccagtgtaggctccaaacaggcatgcgaggtgcctttggaaagccccggggcactgtggccagttggccaagttatcatctccatccgcaccaagctgcagaacaaggagcacgtgattgaggccctgggcagggccaagttcaagtttcctggctgccagaagatccacatctcaaagaagtggggcttcaccaagttcaatgccaatgaatttgaagacatggtggctgagaagcggctcatcccagatggctgtggggtcaagtaccttcccagttgtggccctctagagaagtggtgggccctgcactcatgagggctttcactgtgcttccccctcttaatactcacc*aataaa*ttctacttcctgtccacctaaaaaaaaaaaaaaagaaaaagaatgctttaacttcaa**ttacccctctctcttatgtacgtttagtctgttattttagttttatcctgattccctcgcataaacattattattatattaaaaaaaagaaaagaaaagaaaatgtgtagagccgggcacggtggctcatgcctgtaatcctagcactttgggaggctgaggtaggcggatcacaaggtcaagagat*ccagacca*tcctggccaatatggtgaaaccccgtctctactaaaaatacaaaaattagccggacgtggtggtgcatgcctgtaatcccagctactcaggaggctgaggcaggagaattgcttgaacctgggagacggagatgcagtgagccaagatcatgccactacactccagcctgggcaacagagtgagactccacctcaaaaacaacaacaacaatgacaacaaccccaaaaacaaacatgaataaatatcattctccaataaaatcattcatgccagttagggaaccagttaggaggttgttactggaaaccaggtgaa

**dog**

acgcagagaacttagagctgaaaaagaaaactattgtggggtttagggaaaatatttcaaagcagtggcctaggctggggtaaggactgggacaggagggggctggtgttatggaatgaaagtgatgtctcag*ccagacca*acccccagcatgacatctgcaggaaccagagggctcacgggattcaaagaagcatgtagttctggaatagctctccatttgattggttgtatcttaacattttcaatagggaaaaaaaaaaacaggaggtaaataatgtaaaatcaaagtatggaattgaggccataaaactatgtcctgtgagttcaattgtttgtgcagttcttgttaggaccttcttatctctcacctgaattacagtaacaacctcctaactggtccactagtgg

**human AGAGATTTTATTGGAGAATGATATTTATTCATGTTTTCCCTAATGAACCCTTTAACTTTACTCCAGTTACCCTTAAGATTGAGT
dog	AGGGATTTTTCTGAAATTCGCATTTCTGTGCATCTTCCCCAATTGAGTTCTTTCATTGCACTCTGGCTGCCCCTAAGATGGAGT
 ** ****** ** *	*	**	*	** *** * ***	**** * * **** * * *** ****** ******

This hominoid-specific insert comprises a U70 Type 3 snoRT and repetitive sequences (underlined). The part of the human insert included in the CA442867 EST is in bold characters. The corresponding site in the dog genome is occupied by an unrelated sequence.

human : chr12:74,368,140-74,369,368

dog: chr15:21,846,739-21,847,316

**23- Primate-specific U70 Type 3 snoRT (extragenic) ; parental gene : RPL10.**

```
Cow   CTAAACACTGTCTTTCTGTTCAAT CTGTCACTTTGATTTCCTCTGTCATCACCAATTCTGTGACCTGACAGCTTCTCTTTAAAT
Human ATGAACACTGCCTTTCTCTAGAGTTCTGTCACTCTGATTACCACTGTCACCCCGAATTCTGGAACTCAGCAGA  CTTTTAAAAT
       * ******* ****** *  * * ******** ***** ** ****** * * *******  **    ***   ** ** ****

tattcatctgcagtcaattaagccagctgtactcctttcctcacaggggctcggtgtaatggctgcaaacaacagcctcctttgttgtatacacagcttgtttcttgtatgggttgctctaaagaacataggagacagccgtttccaatgtatgtttatgcctctgacctttgaccttgcactgttcccattgtaggctccagacaggtgtgcagggtatctctagaaagccccagggtaccatggccagaatttacattggtaagtcatcatgtccatccataccaaggtgcagaacaaggagcatgtgactgagtccctacacagggccaagttcaagttgcttggccacatcacaaagaaatggggctctatcaagtttaatgcagatgaatctgaagacatggtagttgagaagtgtcttatctcagatggctgtggggtcaaatgcatccccagtcatggctccgcaacaagtgtcaggccttgcactcaggaaggcttccactgttctgctgtctcttaggctcaccaatactttctgacaaagaaaaaaaaa

Human TGTTAGCATATGTTTAACCAAACCCTTTCTTTCCTTCATCCTTCTTATCAAAGTAGAGGCATGTAGAATTTTCCAAATTA
Cow   TGTTGGCCTGCGTATAACTACACCCTTTCTTTCCTTCATTCTTCTGGTACAA TAGAGACATGTAGCTTTTTCAAAATTA
      **** ** *  ** **** * ****************** *****  *  ** ***** *******  ***** ******

Part of this snoRT (bold characters) constitutes the second exon of EST CF130057, in the reverse orientation. See Figure S5B.
human : chr6:82,530,370-82,531,091
cow : scaffold69:874,034-874,196
```

**24- Primate-specific U70 Type 3 snoRT in the BC030122 mRNA gene (sense) ; parental gene RPL10**

**dog	GAACTATCACG ATGAGAGATCACTTGAAGGCGATATTTTGCAGATATCACCCAAGGTTGATAAAATCCAGAATTCTC**

**human GAATGATCACTCATGAAAGATTACTAAGAAGTGATATTTTGCAGACCTTATTCAAGGTTGATAAAATCCA AATTCTT**

***** ***** **** **** ***	* * ************* * * ****************** ********

gccttttgggtctctgaacagcaccatggtggctggcaagaataagcgttttatgaaaggtggctaaaaggaagccaagaagaaagg*ggttgatccattttctaagaaagactggtatcacgtgaaagcacctgctatggtcagtataaggagtattggaaagacactaggattcaaggaacaagaattgcacct[*]gatgaccttaagggtcatgtgtttgaagtgggtcttgctgatctgcagaataatgaagttgcatttagaataaactagctgattactgaagatgttcagggcaaaaactgcctgactaacttccatggcatggatcttacccatgacaaaatgtgttccatggtcaaaaaatggtag*accatgactgaagctcatgtcaatgtcaagactaccgatggttatttgcttggtcagttctgtattgattttatagaaaatgcaacaatcagatacagaagacctcttatgctcagcaccaacaggtctgtcaaattcagaaggagatgatggaaatcataaacacagaagtgcagacaaacgacttcaaaaaagtgttcaataaatt*gattccagacaacattggaaaagacatagaaaatgcttgccaatctatttatcctcttcatgatgtattcattagaaaagtaaaaatgctgaagaaacccaagtttgaat*tgggaaaactaatagagtttcatgatgaagttagttgttctggaaaagctactggggacaagacaggtgctaacgttgaatgagctgatggatatgagccacaagtccaagaatctgtttaaagtttgattttaatagtggca**aataaa**aaagcctatctgtctgcagccaattgagttgaccgagctcttttcctcctgggggcttggtgtgcaatggctgcaaacagcagattccttggtagtgtatgcagcctgcttcttgtatgggttgcctctaagggaccttgaaggcagccctttccagtgtatgttcatgtctctgaccttatgctgtcccagtgtaggctgtagagaggtgtgcaagctgcctttggaaagtttcagagtatcatagcctgggttcatattggccaagtcatcaggtccatctgcaccaagcagaacaaggagcatgtgattgaggccctacacagtgccaagttcaag*ttcctggctgccagaagatccacatctcaaagaagtggtgctttatcaagttcaatttgaatgaagttggagacatggtggctgagaagtagctcatcccagatgactgtggagtcaaatacatccccaattgtgactctctggacaaatgacaagctctgtactcatgaagtcttccactgtgtggccatctcctaatgccaacc**aataaa**tcctatccataaaaaaaagagttctatttgtggggaaaaaaaatccaaattctt

**human TAATAAGGTTTCTAACTTCACAGATTCCTGAGTCAATGAATTTGACACCAAGATCCTTATGCAATAAGAAAGCAATG TT**

**dog	TACTAAAGTTTATCAATCCACAGGTTCGTGGGTCAA GAAATTGCTACCAAGATCCTTGGGCAAAAAGGAGACAGAGCTT**

**** *** **** * * * ***** *** ** ***** *** *** ************ **** *** * ** * ****

This primate-specific insertion contains a full-length RPS3A processed pseudogene (magenta, exon-exon junctions marked with stars) followed by a U70 Type 3 snoRT. The overall insert has a single TSD (boxed) and probably results from a RT template switch.

human : chr5:87,713,416-87,715,015

dog : chr3:23,139,003-23,139,158

**25- Human- and chimp-specific U70 Type 3 snoRT (extragenic) ; parental gene RPL10**

**dog	TAAAGTAGTATGCTAATTGTGCAAGAATAAACAGACAAATCAATGAAACAGA	AAAACCCCTAACAAATAAAAGTTTGTATAAG**

**human TAAAATAGTGTGCTAAATGTGCAAAAGCAAACAGGCAAATCAATGAAATAGAGTAGAAATCCCATAACAAATAAAAGTTTGTAGAAG**

****** **** ****** ******* * ****** ************* *** *** *** ******************* *****

ccaattaagccaactgagtttctttcctcacagggacctggtgtgccatggctgcaaacagcagcttccttgtcagggtacgcagactgtttcttggatgggttgctctaagggaccttggagacaggcctttcaaatgtatgttcatgtctctgaccttgcactacccccagtgtaggctccaaacagattccttgggaaagccccagggcactgtggccagggttcacataggccaagtcatcatgtccatgcgcaccaagctgcagaacaagcagcttgtgattgaggacctatgcagggccaagttcaagttacttggctgccagaagatccgcatctcaaagaagtggtgctttaccaaattcaaggtggattaatttttttttttgagatggagctcgctctgttgcccaggctggagtgcagtggcgcaatctcagctcactgcaacctccacctcccaggttcaagcaattctcctgcctcagcctcccaaatagctaggactacaggtgtatgccgccacccccagctaattttttttgtattttagtagagacgggttttcaccatattgcccagtctggtctcgaacttctgagctcagggaatccgtccgtcttggcctcccaaagtgctgggattacaggtgtgagccaccacgctgggcccaaggtggatgaatttgaagatacggtggctgagaagtggctcctcctacatggctgtggggccaagtacatccccagtcgtggtcctctggacaagtgtccggccctgctctagtgagggcttccactgtgctgccccctcctgatgcccaccagtaaatcctacttcctgtccacctatttttaaaaaagtatatataag

**human T ATATTTCATGACAGTGACATTTTCTAATCAATTATGAAATGACCAGTTATTCAGAAAATGCTAAGACAAATTGGTTAAAGATCT**

**dog	TAATAGTCAATGAAAGTGGCATTTTCTGATCAATTAGGAAGGGTGGA TCATTCAGAAAATGCTGGGACAGATTGATTAAAAATGT**

*** *** * **** **** ******** ******** *** * * * ************** **** **** **** ** ***

An AluSx element is inserted is this snoRT at almost the same position as for that presented on Figure S3-4.

human : chr17:23,372,651-23,373,698

dog : chr9:45,709,480-45,709,647
