# Supplementary material for: Mammalian Small Nucleolar RNAs Are Mobile Genetic Elements
Source: PLoS Genet. 2006 Dec 8;2(12):e205. doi: 10.1371/journal.pgen.0020205 (PMC1687206; doi:10.1371/journal.pgen.0020205)
Supplement: Supplementary file 7 [file pgen.0020205.st001.doc]

| **Figure** | **TSD** | **Length** |
| --- | --- | --- |
| Type 1 snoRTs |  |  |
| S1-1 | gcttAAAATAGCAAGCATTTA | 17 |
| S1-2 | acagAAATCTCCATGCTGG | 15 |
| S1-3 | ttagGCTGATAAATA | 11 |
| S1-4 | agttGAAAAATA | 8 |
| S1-5 | aaaaTCTACACAGCG | 11 |
| S1-6 | atagAAAGTACTGTTCTT | 14 |
| S1-7 | gctaCAAATGTTTGTTTCC | 15 |
| S1-8 | ccagAAAAAGTGAAAAGAC | 15 |
| S1-9 | aaagTTAGATTGTGGGGC | 14 |
| S1-10 | ttaaAACTTTTAAGATGCTT | 16 |
| S1-11 | ttatAAGGAAAATCT | 11 |
| S1-12 | aagtGAAAACTTAATTTGGA | 16 |
| S1-13 | aaaaGCAAGGAGAA | 10 |
| S1-14 | ccttTAAAGACACACTTTTC | 16 |
| S1-15 | ttgtAAAAGAAAAAAAAAA | 15 |
| S1-17 | acttCAAAGATATCTTCCCC | 16 |
| S1-18 | cttaAAAGGAGGACTGTG | 14 |
| S1-19 | attgAAAACTTCAGTCAA | 14 |
| S1-20 | tgatAAAGTCTCATTTATTCTCC | 19 |
| S1-21 | acaaGGGCCTCCCT | 10 |
| S1-22 | aaaaCTGCCATCA | 9 |
| S1-23 | ggatAAGAAAATAAATATG | 15 |
| S1-25 | ataaAAATGAATATATTCT | 15 |
| S1-27 | acttAAGACCTGAACCCCC | 15 |
| S1-28 | tcagAGACCCTCTTTTTC | 14 |
| S1-29 | ccatAGAAACCTTTCTGT | 14 |
| S1-30 | ccctAAGTTTTGTTCATCTTAC | 18 |
| S1-32 | aatgAAAAAAACAGGCA | 13 |
| S1-33 | ttttAAAAAGTAATTAA | 13 |
| S1-35 | gggtTGATAAAT | 8 |
| S1-36 | aaaaAAGATTTCATTGTG | 14 |
| S1-37 | tgtgAAAGGGTCTCTTG | 13 |
| S1-39 | catcAAGAGCCAAGGAA | 13 |
| S1-40 | tgtcAAGAAATC | 8 |
| S1-42 | aataAAAAGTTATGAAAGGA | 16 |
| S1-43 | cataAAATCCTGTGCTTTT | 15 |
| NS | gattAAGAAGATCAAC | 12 |
| NS | aaacTAGCACATTTGAT | 13 |
| NS | ggagAAAGAACAGGGAGCTT | 16 |
| NS | acttAAGACCTGAACCCCC | 15 |
| NS | gtttGAAAACCACAC | 11 |
| Type 2 snoRTs |  |  |
| S2-1 | ctttAAGATTCATAAAAGTG | 16 |
| S2-2 | ttaaAGAAATAAGACTCTTC | 16 |
| S2-3 | atctAAATAGAAGCTGA | 14 |
| S2-4 | atttAATAGTCTCGTCGA | 14 |
| S2-5 | tttcAAGAATGCTGAATTGG | 16 |
| NS | tattAAGAAAC | 7 |
| NS | atatAAGAATCCACTGTCTT | 16 |
| NS | taacACATATGGCAA | 11 |
| Type 3 snoRTs |  |  |
| S3-1 | aatgAAAGTCATTACC | 12 |
| S3-2 | attaAGAGAACATATAAATCCT | 18 |
| S3-3 | ctgtAAGATTACGGAGGT | 14 |
| S3-4 | aagaCTGATTTTT | 9 |
| S3-6 | agttAAGAGTTTCTAAAACA | 16 |
| S3-7 | aaaaAAAAAAAAAGTCC | 13 |
| S3-8 | tgttTAAAATATGATGGGGA | 16 |
| S3-9 | ttttTAAAAACAAATTGTG | 15 |
| S3-10 | ttttAAACTCTAGTTA | 12 |
| S3-11 | ttagATCTCTTGAAATA | 13 |
| S3-12 | tgaaAAACAAACAAACA | 13 |
| S3-13 | tgttAAAATTTATACT | 12 |
| S3-14 | ttaaAAAGGACCT | 9 |
| S3-15 | tgaaATACTCCAGATCA | 13 |
| S3-16 | agatAGAAAAGTGGAAAAG | 15 |
| S3-17 | gatcAAGACCTGAGCTAA | 14 |
| S3-18 | ctaaGAGTGTAGAAAGC | 13 |
| S3-19 | tcttAAAAAAATAAATAA | 14 |
| NS | gattATAAACTTTAACAA | 14 |
| NS | gatcAAAAAAGAAACCAC | 14 |
| NS | agatAAACATAAAAGAATGG | 16 |
| NS | aataAGATATAAAAGTTTA | 15 |
| NS | ttttGAAAAATAGATGTG | 14 |
